# Akt2 causes TGFβ-induced deptor downregulation facilitating mTOR to drive podocyte hypertrophy and matrix protein expression

**DOI:** 10.1371/journal.pone.0207285

**Published:** 2018-11-16

**Authors:** Falguni Das, Nandini Ghosh-Choudhury, Doug Yoon Lee, Yves Gorin, Balakuntalam S. Kasinath, Goutam Ghosh Choudhury

**Affiliations:** 1 Department of Medicine, UT Health San Antonio, San Antonio, Texas, United States of America; 2 Department of Pathology, UT Health San Antonio, San Antonio, Texas, United States of America; 3 VA Research, South Texas Veterans Health Care System, San Antonio, Texas, United States of America; 4 Geriatric Research, Education and Clinical Center, South Texas Veterans Health Care System, San Antonio, Texas, United States of America; Hopital Tenon, FRANCE

## Abstract

TGFβ promotes podocyte hypertrophy and expression of matrix proteins in fibrotic kidney diseases such as diabetic nephropathy. Both mTORC1 and mTORC2 are hyperactive in response to TGFβ in various renal diseases. Deptor is a component of mTOR complexes and a constitutive inhibitor of their activities. We identified that deptor downregulation by TGFβ maintains hyperactive mTOR in podocytes. To unravel the mechanism, we found that TGFβ -initiated noncanonical signaling controls deptor inhibition. Pharmacological inhibitor of PI 3 kinase, Ly 294002 and pan Akt kinase inhibitor MK 2206 prevented the TGFβ induced downregulation of deptor, resulting in suppression of both mTORC1 and mTORC2 activities. However, specific isoform of Akt involved in this process is not known. We identified Akt2 as predominant isoform expressed in kidney cortex, glomeruli and podocytes. TGFβ time-dependently increased the activating phosphorylation of Akt2. Expression of dominant negative PI 3 kinase and its signaling inhibitor PTEN blocked Akt2 phosphorylation by TGFβ. Inhibition of Akt2 using a phospho-deficient mutant that inactivates its kinase activity, as well as siRNA against the kinase markedly diminished TGFβ -mediated deptor suppression, its association with mTOR and activation of mTORC1 and mTORC2. Importantly, inhibition of Akt2 blocked TGFβ -induced podocyte hypertrophy and expression of the matrix protein fibronectin. This inhibition was reversed by the downregulation of deptor. Interestingly, we detected increased phosphorylation of Akt2 concomitant with TGFβ expression in the kidneys of diabetic rats. Thus, our data identify previously unrecognized Akt2 kinase as a driver of TGFβ induced deptor downregulation and sustained mTORC1 and mTORC2 activation. Furthermore, we provide the first evidence that deptor downstream of Akt2 contributes to podocyte hypertrophy and matrix protein expression found in glomerulosclerosis in different renal diseases.

## Introduction

Kidney glomerular filtration barrier consists of fenestrated endothelial cells, glomerular basement membrane and glomerular epithelial cells called podocytes. Podocytes are terminally differentiated cells, which lack proliferative potential [1]. Multiple mutations found in nephrotic syndrome occur in proteins present in the podocytes [2]. Upon injury, podocytes undergo hypertrophy, which results in albuminuria and glomerulosclerosis seen in many glomerular diseases such as focal segmental glomerulosclerosis, IgA nephropathy, lupus nephritis and diabetic nephropathy [1–3]. During progressive kidney injury, TGFβ contributes significantly to the pathophysiology of glomerulosclerosis and urinary albumin excretion especially in diabetic nephropathy [4]. Overexpression of TGFβ causes proteinuria and progressive kidney disease [5]. On the other hand, anti- TGFβ antibody prevented glomerulosclerosis, glomerular hypertrophy and renal dysfunction in rodent models of diabetic nephropathy [6, 7]. More recently, Niranjan et al has shown a significant role of Notch in regulating podocyte damage in glomerulosclerosis by TGFβ [8]. Finally, a hypomorphic Akita diabetic mouse model where the expression of TGFβ was reduced to 10% of the wild type showed significant amelioration of glomerular filtration rate and albuminuria. In contrast, 300% increase in expression of TGFβ caused glomerulosclerosis and albuminuria [9].

Binding of TGFβ to its type II receptor initiates canonical signaling via type I receptor serine threonine kinase, which binds and phosphorylates the receptor specific Smads, Smad 2 and Smad 3 at their C-termini. Phosphorylated Smads then dissociate from the type I receptor, heterodimerize with common Smad, Smad 4 and translocate to the nucleus to activate or repress the target genes [10]. Apart from this canonical signaling, we and others have shown that TGFβ uses a noncanonical pathway to activate mTOR (mechanistic target of rapamycin) kinase to induce cell hypertrophy and matrix protein expansion [11–15]. mTOR forms two complexes C1 and C2 that contain common and distinct proteins and exhibit unique substrate specificities. For example, mTORC2 phosphorylates the AGC kinases including Akt while mTORC1 phosphorylates diverse substrates including the mRNA translation inhibitors and activators [16]. Recently, the protein deptor has been identified as a component of both the complexes [17]. Deptor is an endogenous inhibitor of both mTOR complexes. We have shown recently that TGFβ inhibits expression of deptor [18]. Here we report the signal transduction mechanism, which regulate TGFβ induced deptor suppression. We show that deptor downregulation requires the Akt isoform Akt2, leading to activation of mTORC1 and mTORC2 necessary for podocyte hypertrophy and matrix protein expression.

## Materials and methods

Materials: Tissue culture materials and OPTIMEM medium were purchased from Thermo Fisher. TGFβ 1 was obtained from R & D Systems, Minneapolis, MN. Protease inhibitor cocktail, phenylmethylsulphonylfluoride, NP-40, Na_3_VO_4_ and and β-actin and fibronectin antibodies were purchased from Sigma, St Louis, MO. Following antibodies were obtained from Cell Signaling, Danvers, MA: phospho-S6 kinase (Thr-389), phospho-4EBP-1 (Thr-37/46), phospho-Akt2 (Ser-474), phospho-SGK1 (Ser-422), phospho-rps6, phospho-NDRG1 (Thr-346), S6 kinase, 4EBP-1, Akt1, Akt2, Akt3, SGK1, rps6, mTOR and NDRG1 (N-Myc downstream-regulated gene 1 protein). Antibodies for deptor, mTOR, p85, PTEN, and siRNAs for Akt1 and Akt2 were purchased from Santa Cruz, Dallas, TX. TGFβ antibody was purchased from Abcam, Cambridge, MA). Anti-HA antibody was obtained from Covance, Princeton, NJ. The PI 3 kinase inhibitor Ly 294002 (Ly) and pan Akt kinase inhibitor MK 2206 (MK) were purchased from CalBiochem, San Diego, CA and Selleck Chemicals, Houston, TX, respectively. The PVDF membrane for transferring proteins was obtained from Perkin Elmer, Shelton, CT. The transfection reagent FuGENE HD was purchased from Promega Inc, Madison, WI. The inter-SH2 domain deleted p85 regulatory subunit of PI 3 kinase, which confers dominant negative function to the enzyme and HA tagged PTEN were described previously [19, 20]. The phosphorylation deficient mutant Akt2 expression vector (pHA AKT2 T309A/S474A) was obtained from Addgene (plasmid #60128).

Cell culture and treatment: Rat visceral glomerular epithelial cells (podocytes) were cultured in RPMI in the presence of antibiotic and 10% fetal bovine serum as described previously [21]. In these cells, we confirmed the expression of nephrin and synaptopodin, two specific markers synthesized by the renal glomerular podocytes *in vivo;* rat mesangial cells did not express these proteins (S1 Fig). Also, these cells express highly specific podocyte marker podocin [22]. The cells were starved in serum free medium for 24 hours prior to incubation with TGFβ (2 ng/ml) for indicated periods of time. We routinely employ these cells between passages 9 and 14.

Animals and preparation of the glomeruli: Male Sprague-Dawley rats (200–250 gm) were used. Streptozotocin (STZ, 55 mg/kg body weight) in sodium citrate buffer (0.01 M, pH 4.5) was injected via the tail vein. Control rats received the same amount of sodium citrate buffer [23]. Blood glucose concentration was determined with LifeScan One Touch glucometer at 24 hours post-STZ injection and monitored periodically. Animals were kept at the UT Health San Antonio animal facility and they had free access to food and water. The animal protocol was approved by the UT Health San Antonio Animal Care and use Committee. At 3 months, rats were euthanized and both kidneys were removed. The renal capsule and the medullary tissue were removed from the cortex. A piece of kidney cortex was flash-frozen in liquid nitrogen. The dissected cortex was sliced in PBS with a razor blade and passed through a 305 micrometer stainless steel sieve using a spatula. The resulting suspension was passed several times through 312 and 150 micrometer nylon sieves. The glomeruli were retained by the 150 micrometer sieve. They were washed several times with PBS and then centrifuged at 600 x g to obtain a pellet [24]. The pellet was frozen at -70°C for future use.

Lysis of cells and tissues, immunoblotting and immunoprecipitation: At the end of the incubation, the cell monolayers were washed twice with PBS. RIPA buffer (20 mM Tris-HCl, pH 7.5, 150 mM NaCl, 1% NP-40, 5 mM EDTA, 1 mM PMSF and 0.1% protease inhibitor cocktail) was added to the cell monolayer and incubated at 4°C for 30 minutes. The cells were scraped off the dishes using cell plastic scraper and transferred to the centrifuge tubes. Similarly, renal cortex and glomeruli were lysed in the RIPA buffer. The extracts were spun at 10,000 x g for 20 minutes at 4°C. The pellets were discarded and the supernatant was collected in fresh tube. Protein concentration was determined in the supernatant using BioRad protein estimation reagent as per vendor’s instruction. BSA was used as standard. 20 μg of protein was separated by SDS polyacrylamide gel electrophoresis. The proteins were transferred to PVDF membrane at 4°C for 4–6 hours in an electroblotter using the transfer buffer (20 mM Tris, 190 mM glycine and 20% methanol). Prior to transfer, the PVDF membrane was activated for 1 minute in methanol. After the transfer, the membrane was blocked in 4% dry milk (blotting grade Blocker, BioRad) prepared in PBST (0.2% Tween 20 in PBS) for 1 hour. The membrane was washed three times with PBST (pH 7.4). Primary antibody (1:1000 dilution) was incubated with the membrane in PBST for 6 hours on a rocking platform at 4°C. After the incubation, the membrane was washed three times with PBST. HRP-conjugated secondary antibody (1:10000 dilution) in PBST was added for 1 hour with shaking at room temperature. The membrane was then washed three times with PBST. The excess reagent was removed and the membrane was covered with a transparent plastic wrap. The protein signal on the membrane was developed using Super Signal West Pico Plus Chemiluminiscent reagent (Thermo Scientific). The protein band was visualized by exposing to X-ray film in dark room using a Kodak X-ray machine [25, 26]. For immunoprecipitation, washed cell monolayer was lysed in IP buffer (40 mM HEPES, 0.3% CHAPS, pH 7.5, 1 mM EDTA, 120 mM NaCl, 1.5 mM Na_3_VO_4_, 10 mM pyrophosphate, 10 mM glycerophosphate, 50 mM NaF and 0.1% EDTA free protease inhibitor cocktail) and the extracts were cleared and protein was estimated as described above. One hundred microgram of protein was immunoprecipitated with the indicated antibodies as described [13, 27]. Briefly, the cell lysates were incubated with the antibody at 4°C for half an hour. Then protein G-sepharose beads were added to the reaction and rotated overnight at cold room to immunoprecipitate the protein. The immunebeads were washed three times in IP buffer and resuspended in SDS sample buffer. The proteins were separated by SDS-polyacrylamide gel electrophoresis and immunoblotted as described above.

Immunofluorescence: Frozen cortical tissue embedded in OCT compound medium cryomold were cut at 10 micrometer thick cryosections, mounted on adhesion slides and stored at -80°C. The cryosections were air dried at room temperature for 10 minute, fixed in ice cold acetone for 10 minutes and air dried. After rehydration with 0.1% BSA in PBS, the cryosection was blocked with donkey normal serum for 20 minutes. Rabbit phospho-Akt2 (Ser-474) antibody (Abcam ab38513, 1:20 dilution) was incubated with the cryosection for one hour in the wet chamber. The section was washed 3 times for 5 minutes each with 0.1% BSA in PBS followed by incubation with Alexa Fluor 555 donkey anti-rabbit IgG (Invitrogen; 1:200 dilution) for 30 minutes in wet chamber. The slide was then rinsed in 0.1% BSA/PBS three times for 5 minutes each. The stained slide was mounted with coverslip (No1, 25 mm round) using ProLong Diamond Antifade mounting medium (InVitrogen). The fluorescence was visualized at excitation/emission wavelengths 555 nm/565 nm using an Olympus FV-1000 confocal laser scanning microscope at the UT Health at San Antonio institutional core facility.

Transfection: Cells were used at 80–90% confluency. The culture medium was removed and the cell monolayer was washed once with PBS. OPTIMEM medium was added to the cells and kept in the cell culture hood. In a sterile tube, the expression plasmid or control vector (500 ng) was mixed with FuGENE in OPTIMEM according to vendor’s instruction and incubated at room temperature for 15 minutes before addition to the cells. The cells were then incubated at 37°C for 6 hours after which complete medium was added. At 24 hours the cells were serum-starved and treated with 2 ng/ml TGFβ as described above [28, 29].

Measurement of Hypertrophy: After incubation, the cell monolayer was washed with PBS, trypsinized and resuspended in the medium. A drop of cell suspension was used to count the cell number in a hemocytometer. The cell suspension was centrifuged at 4000 x g at 4°C. The cell pellet was washed once with PBS and lysed in RIPA buffer as described above. Total protein content was measured and ratio of total cellular protein to the cell number was determined. The increase in this ratio was considered as cell hypertrophy as described previously [28, 30].

Statistics: Mean of indicated measurements is shown. The significance of the data was determined using the GraphPad Prism software. ANOVA followed by Students-Newman-Keuls analysis was used to determine the significance. A p value < 0.05 was considered significant change.

## Results

**Fig 1:**
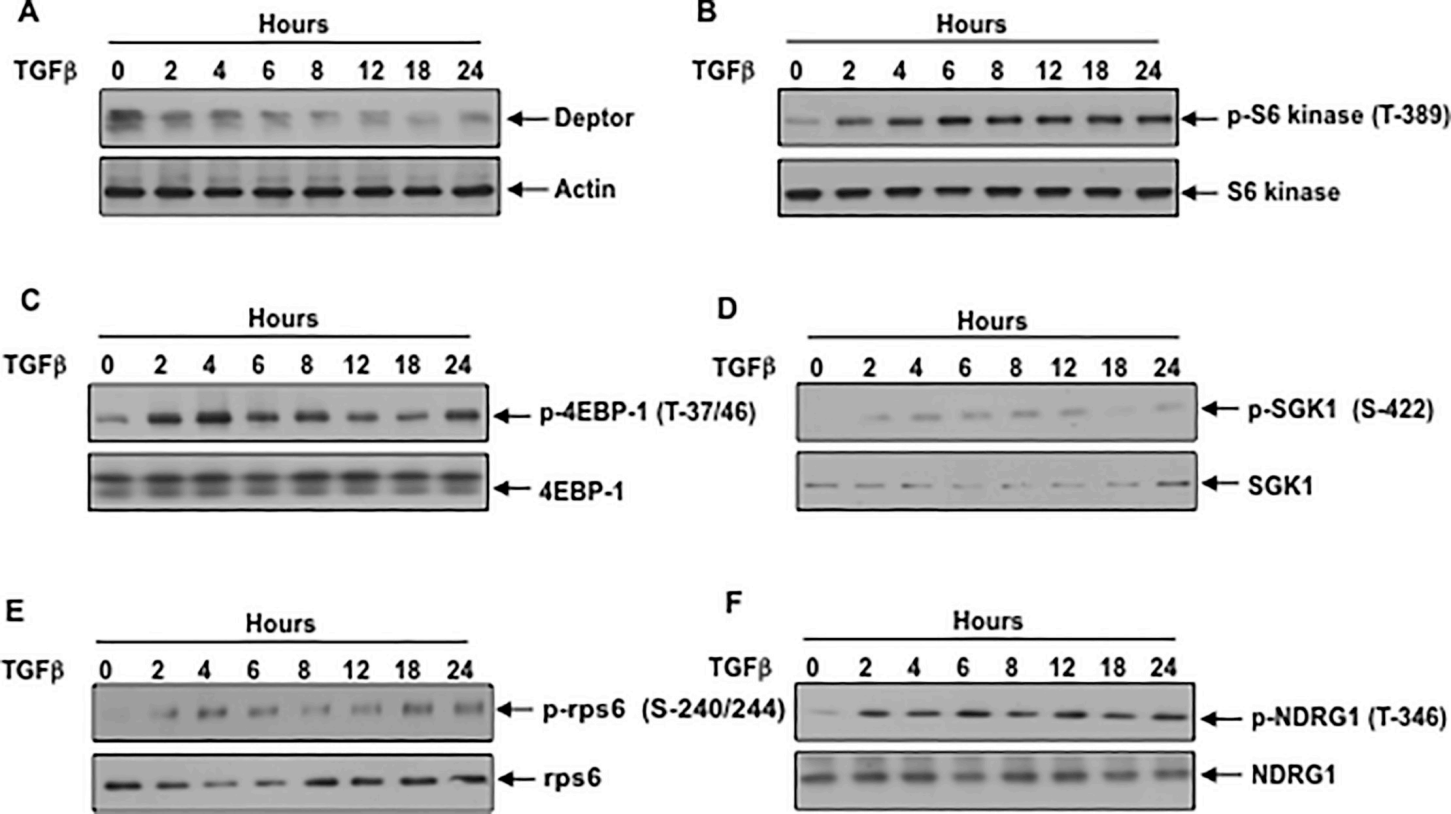
TGFβ downregulates deptor in association with sustained activation of mTORC1 and mTORC2. Serum-starved podocytes were incubated with 2 ng/ml TGFβ for the indicated periods of time. Cleared cell lysates were immunoblotted with deptor, actin (panel A); phospho-S6 kinase (Thr-389), S6 kinase (panel B); phospho-4EBP-1 (Thr-37/46), 4EBP-1 (panel C); phospho-SGK1 (Ser-422), SGK1 (panel D); phospho-rps6 (Ser-240/244), rps6 (panel E); phospho-NDRG1 (Ser-346), NDRG1(panel F). Representative blots from three independent experiments are shown in each panel. The quantification is shown in the S2 Fig.

PI 3 kinase regulates *TGFβ* induced suppression of deptor: Podocyte dysfunction is a critical event in glomerular diseases. Sustained activation of mTORC1 in podocyte is associated with glomerulosclerosis [31]. A role of mTORC2 has also been established in the pathology of renal glomerular disease [32]. TGFβ acts on different kidney cells including podocytes to induce renal injury [4, 33, 34]. We have recently shown that TGFβ elicits prolonged activation of both mTORC1 and mTORC2, which are necessary for renal cell pathology [13, 35, 36]. Because deptor is a component of both mTORC1 and mTORC2, and an endogenous inhibitor of their activities, we investigated its expression in response to TGFβ Incubation of podocytes with TGFβ decreased the expression of deptor in a time-dependent manner (Fig 1A and S2A Fig). Downregulation of deptor was associated with increased mTORC1 activity as judged by the phosphorylation of its two substrates S6 kinase and 4EBP-1 (Fig 1B and 1C and S2B and S2C Fig). To explore the activation of mTORC2, we examined the phosphorylation of SGK-1, which was identified as the substrate of mTORC2 [37]. Similar to activation of mTORC1, TGFβ increased the phosphorylation of SGK-1 (Fig 1D and S2D Fig). Since phosphorylation of S6 kinase and SGK-1 by mTORC1 and mTORC2 respectively increases their kinase activities, we tested the phosphorylation of their substrates rps6 and NDRG1, respectively. TGFβ enhanced phosphorylation of rps6 and NDRG1 at the same kinetics as S6 kinase and SGK-1 phosphorylation (Fig 1E and 1F and S2E and S2F Fig). These results demonstrate that deptor downregulation by TGFβ is associated with increased activation of mTORC1 and mTORC2.

**Fig 2:**
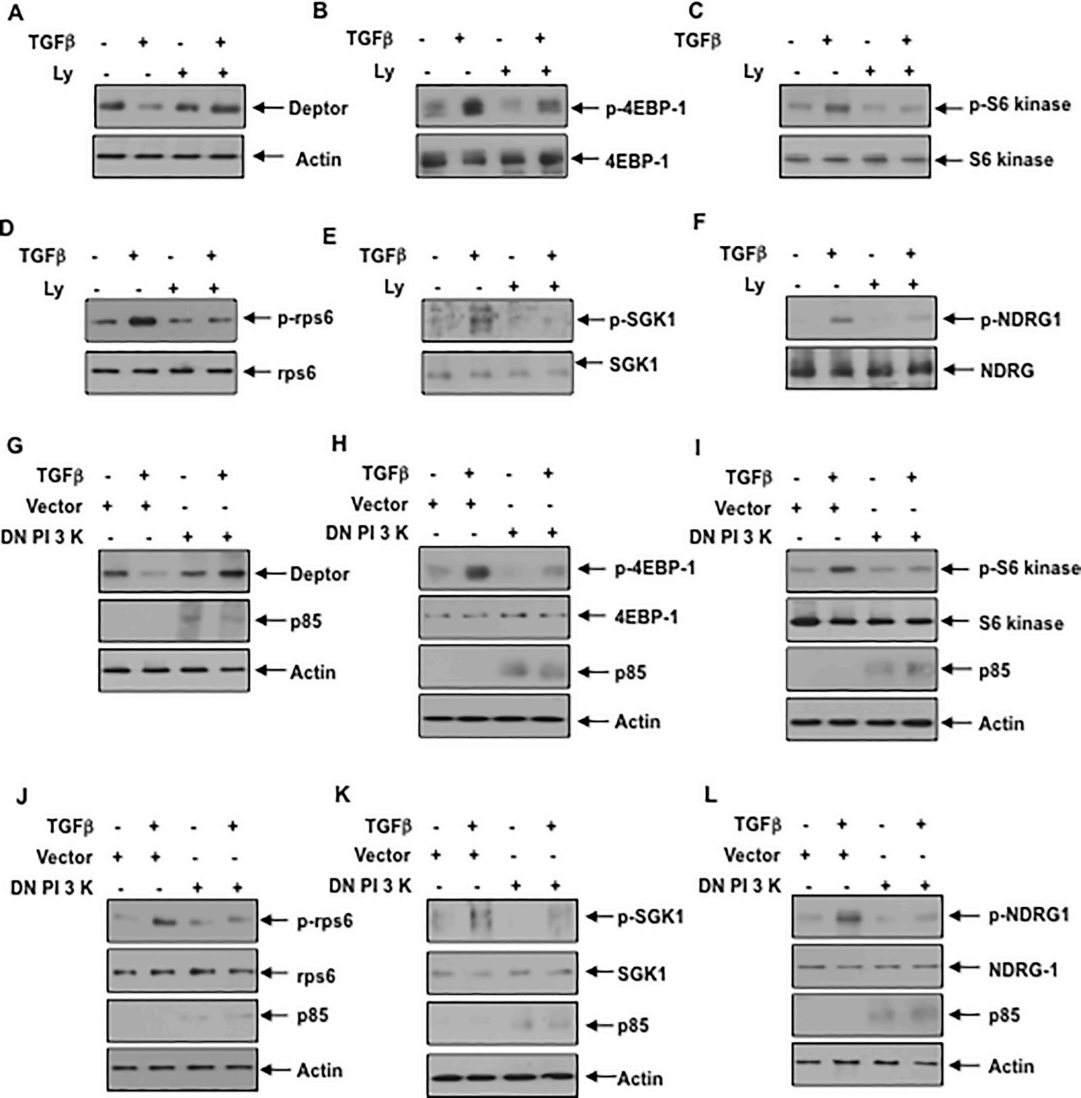
Inhibition of PI 3 kinase blocks TGFβ induced deptor suppression and activation of mTORC1 and mTORC2. (A–F) Serum-starved podocytes were treated with 25 micromolar Ly for none hour prior to incubation with 2 ng/ml TGFβ for 24 hours. The cell lysates were immunoblotted with the indicated antibodies. (G–L) Podocytes were transfected with the mutant p85 PI 3 kinase subunit as described in the Materials and Methods. The transfected cells were serum starved and incubated with TGFβ as described above. The cell lysates were immunoblotted with the indicated antibodies. Representative blots from four independent experiments are shown in each panel. The quantification is shown in the S3 Fig.

The mechanism of deptor downregulation is poorly understood. It was previously reported that PI 3 kinase activity is necessary for activation of both mTORC1 and mTORC2 [38, 39]. Therefore, we examined the involvement of PI 3 kinase using its inhibitor Ly 294002 (Ly). We have routinely used Ly at a concentration of 25 micromolar to block PI 3 kinase activity in renal cells [20, 40, 41]. TGFβ induced inhibition of deptor was reversed by Ly (Fig 2A and S3A Fig). Consequently, the prolonged activation of mTORC1 and mTORC2 was inhibited by Ly as judged by phosphorylation of 4EBP-1/S6 kinase/rps6 (mTORC1 activation) and SGK-1/NDRG-1 (mTORC2 activation) (Fig 2B–2F and S3B–S3F Fig). Similarly, dominant negative PI 3 kinase blocked the downregulation of deptor induced by TGFβ Fig 2G and S3G Fig). Accordingly, dominant negative PI 3 kinase suppressed the TGFβ stimulated activation of mTORC1 and mTORC2 (Fig 2H–2L and S3H–S3L Fig).

**Fig 3:**
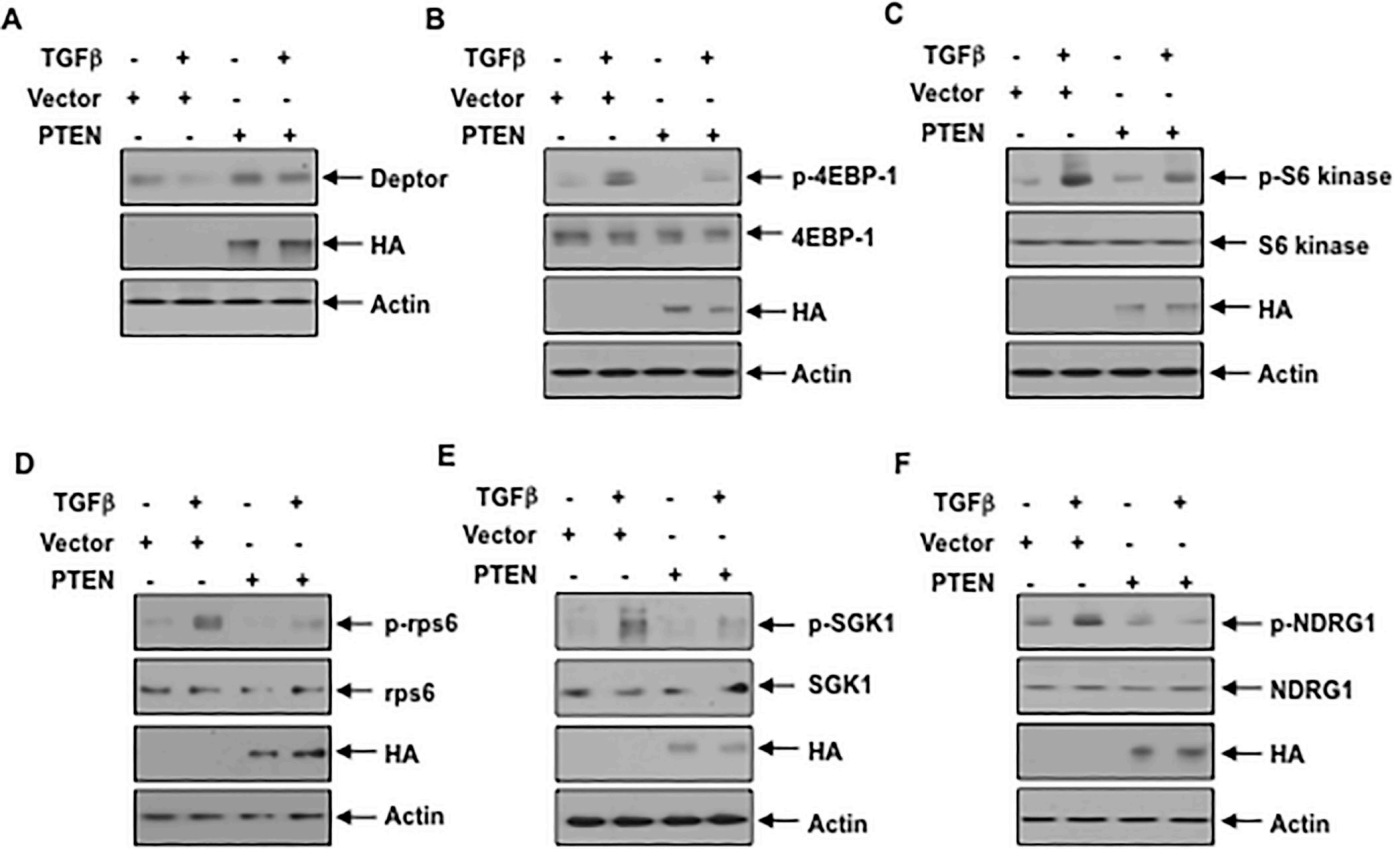
Expression of PTEN prevents TGFβ mediated downregulation of deptor and mTORC1 and mTORC2 activities. Podocytes were transfected with the HA-tagged PTEN expression plasmid. The transfected cells were serum starved and incubated with TGFβ The cell lysates were immunoblotted with the indicated antibodies. Representative blots from four independent experiments are shown in each panel. The quantification is shown in the S4 Fig.

We and others have shown previously that TGFβ inhibits the expression of the tumor suppressor protein PTEN in renal cells [36, 42, 43]. PTEN acts as a negative regulator of the PI 3 kinase signaling [44]. Therefore, to confirm the role of PI 3 kinase shown above, we overexpressed PTEN in TGFβ stimulated podocytes. Expression of PTEN reversed the TGFβ induced inhibition of deptor expression (Fig 3A and S4A Fig). Furthermore, similar to PI 3 kinase inhibition, expression of PTEN blocked the prolonged activation of mTORC1 and mTORC2 induced by TGFβ (Fig 3B–3F and S4B–S4F Fig). These data conclusively demonstrate that TGFβ stimulated PI 3 kinase mediates inhibition of deptor and prolonged activation of both mTORC1 and mTORC2.

**Fig 4:**
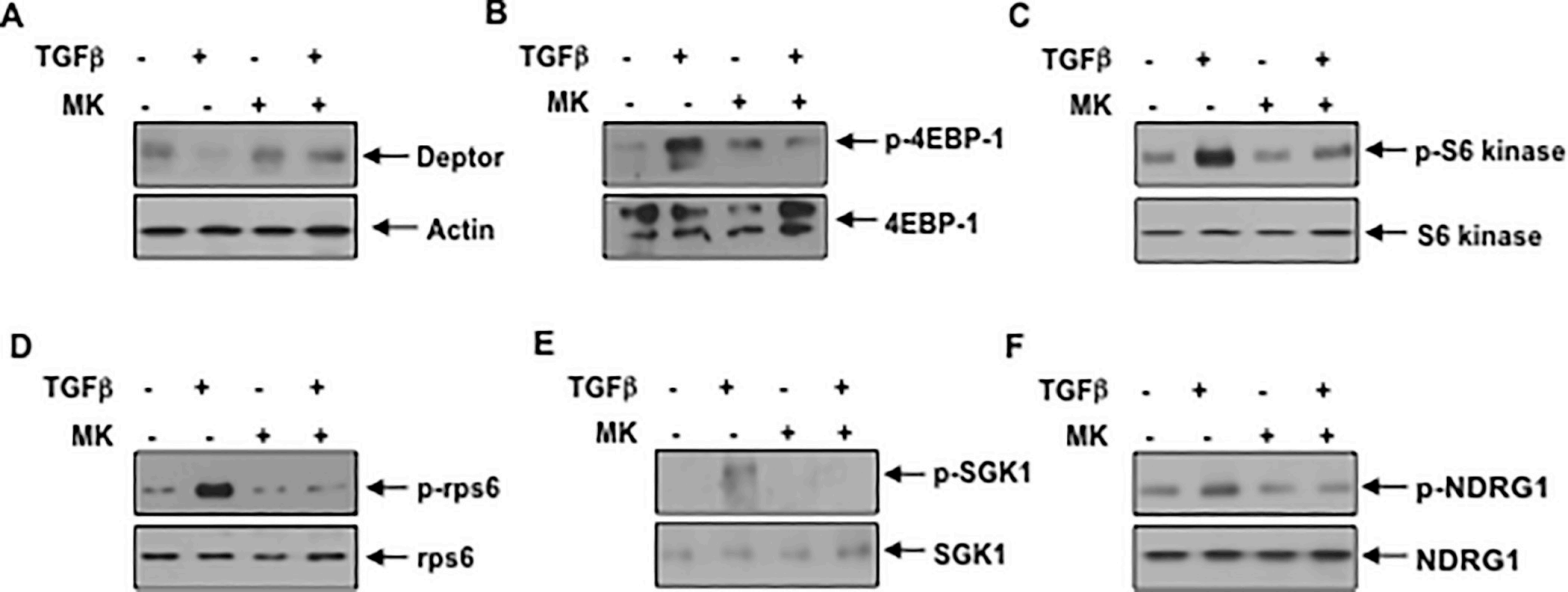
Pan Akt kinase inhibitor MK restores TGFβ induced deptor downregulation and inhibits mTORC1 and mTORC2. Serum starved podocytes were treated with 1 micromolar MK for one hour prior to incubation with TGFβ. The cell lysates were immunoblotted with the indicated antibodies. Representative blots from four independent experiments are shown in each panel. The quantification is shown in the S5 Fig.

Akt kinase is required for deptor suppression: Many biological functions of PI 3 kinase are mediated by its direct downstream kinase Akt. To elucidate its role on deptor expression, we used a specific allosteric inhibitor of Akt, MK2206 (MK) [45]. We and others have used this inhibitor at concentrations between 1 and 5 micromolar [46–48]. Incubation of podocytes with 1 micromolar MK reversed the inhibition of deptor expression by TGFβ (Fig 4A and S5A Fig). Furthermore, MK blocked TGFβ stimulated activation of mTORC1 and mTORC2 (Fig 4B–4F and S5B–S5F Fig). These results show the requirement of Akt kinase in deptor downregulation and mTOR activation.

**Fig 5:**
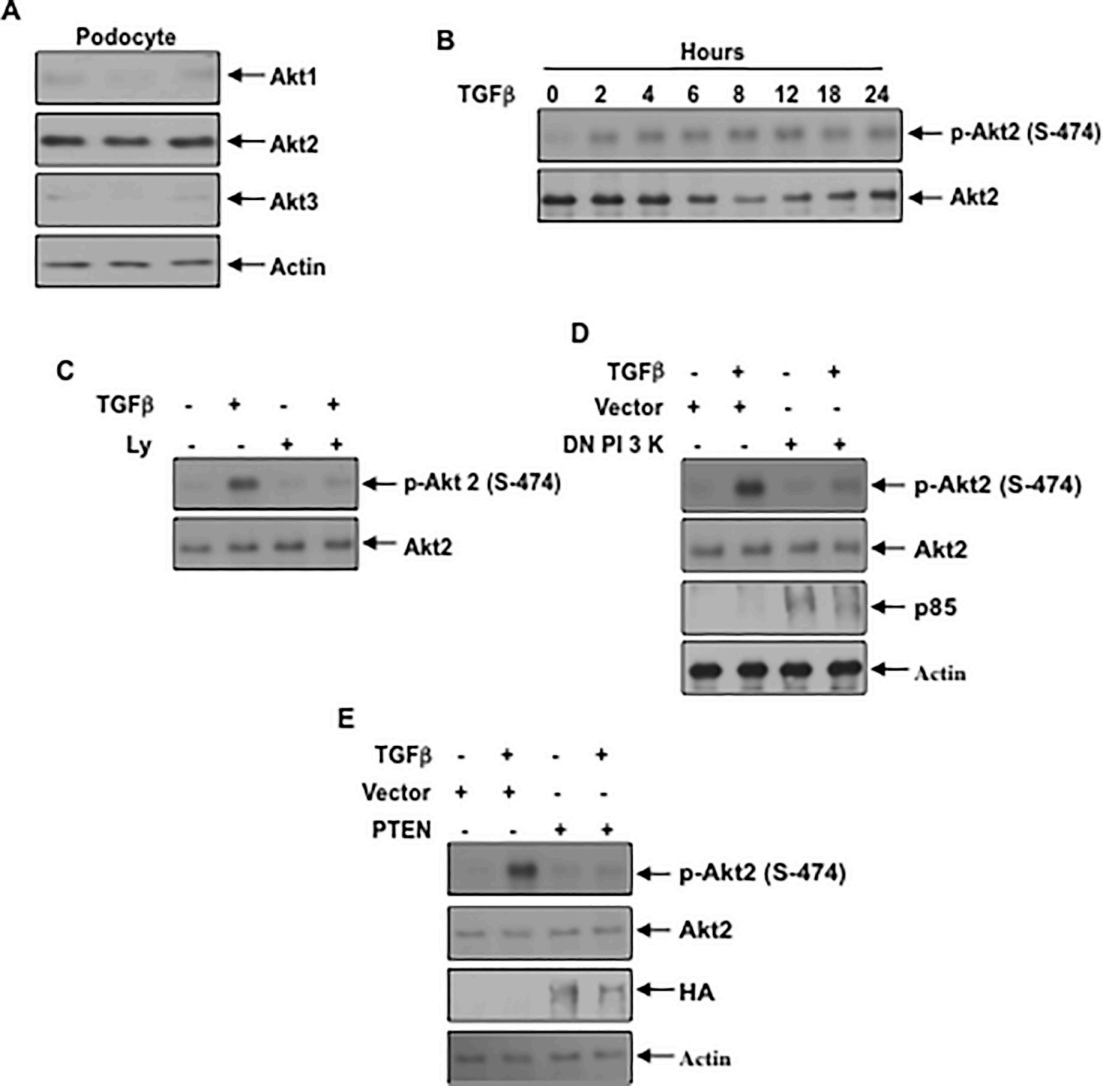
Akt2 is abundantly expressed in podocytes and PI 3 kinase regulates its activation by TGFβ (A) Podocyte lysates from independent dishes were immunoblotted with isoform-specific Akt1, Akt2, Akt3 and actin antibodies. (B) TGFβ increases phosphorylation of Akt2 in a time-dependent manner. Serum-starved podocytes were incubated with 2 ng/ml TGFβ for the indicated periods of time. The cell lysates were immunoblotted with the phospho-Akt (Ser-474) specific antibody. As a control Akt2 specific antibody was used. (C) PI 3 kinase inhibition blocks TGFβ stimulated phosphorylation of Akt2. Serum-starved podocytes were incubated with 25 micromolar Ly before treatment with TGFβ. The cell lysates were immunoblotted with the indicated antibodies. (D and E) Podocytes were transfected with dominant negative p85 subunit of PI 3 kinase (panel D) or PTEN (panel E). The transfected cells were serum starved and incubated with TGFβ The cell lysates were immunoblotted with the indicated antibodies. Representative of 3 and 4 independent experiments is shown for panels B and C, D, E. The quantification is shown in the S7 Fig.

Podocytes predominantly express Akt2 isoform: Three isoforms of Akt kinase exist in the mammals with differential distribution. Akt1 shows ubiquitous expression in many tissues; Akt2 is highly expressed in the liver, skeletal muscle and adipose tissues while Akt3 is predominantly expressed in the brain [49]. To determine the expression of Akt isoforms in the kidney, we used rat renal cortical lysates. Using isotype specific antibodies, we show that Akt2 is the predominant isoform expressed in the kidney cortex, which contains mostly the tubules (S6A Fig). However, expression of Akt1 and Akt3 was also detected, the latter at a very low abundance (S6A Fig). Similarly, rat renal glomeruli express both Akt1 and Akt2, the latter showing more abundant expression (S6B Fig). Next, we determined the levels of Akt isoforms in the podocytes. Similar to the renal glomeruli, podocytes expressed Akt2 more abundantly compared to Akt1 (Fig 5A and S7A Fig). Akt3 was detectable at a low level. Since Akt2 was the predominant isoform, we examined its activation in response to TGFβ. TGFβ time-dependently increased Akt2 phosphorylation at Ser-474 (Fig 5B and S7B Fig) [50]. Treatment of the podocytes with the PI 3 kinase inhibitor Ly blocked the TGFβ induced phosphorylation of Akt2 (Fig 5C and S7C Fig). Similarly, expression of dominant negative PI 3 kinase or PTEN, the inhibitor of PI 3 kinase signaling, blocked this phosphorylation (Fig 5D and 5E and S7D and S7E Fig). These results demonstrate that PI 3 kinase controls the activation of Akt2 in the podocytes.

**Fig 6:**
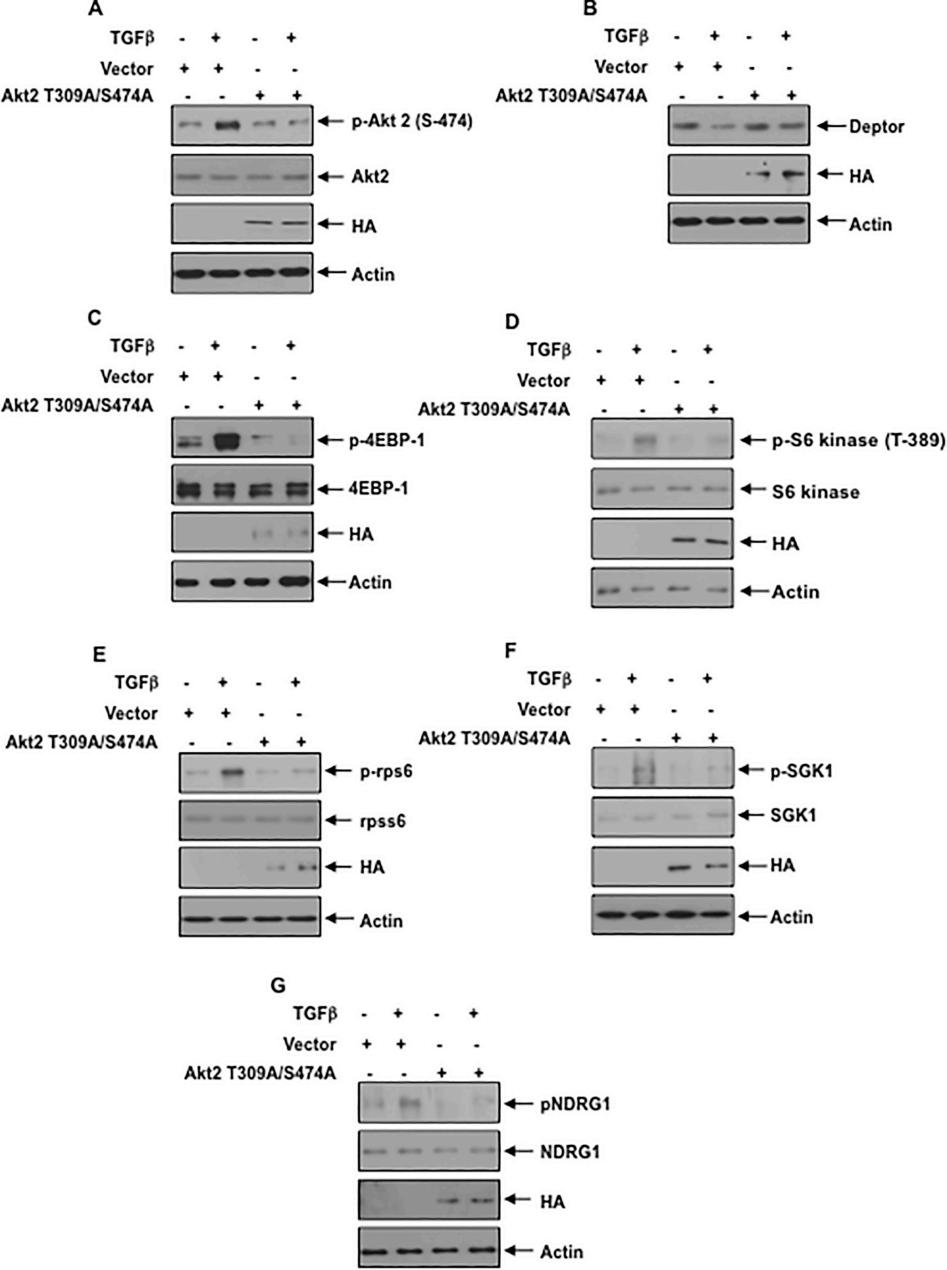
Phospho-deficient Akt2 T309A/S474A prevents TGFβ induced decrease in deptor and activation of mTORC1 and mTORC2. (A–G) Podocytes were transfected with Akt2 T309A/S474A mutant plasmid. The transfected cells were starved and incubated with TGFβ The cell lysates were immunoblotted with indicated antibodies. Representative of 4 independent experiments is shown. The quantification is shown in the S9 Fig.

**Fig 7:**
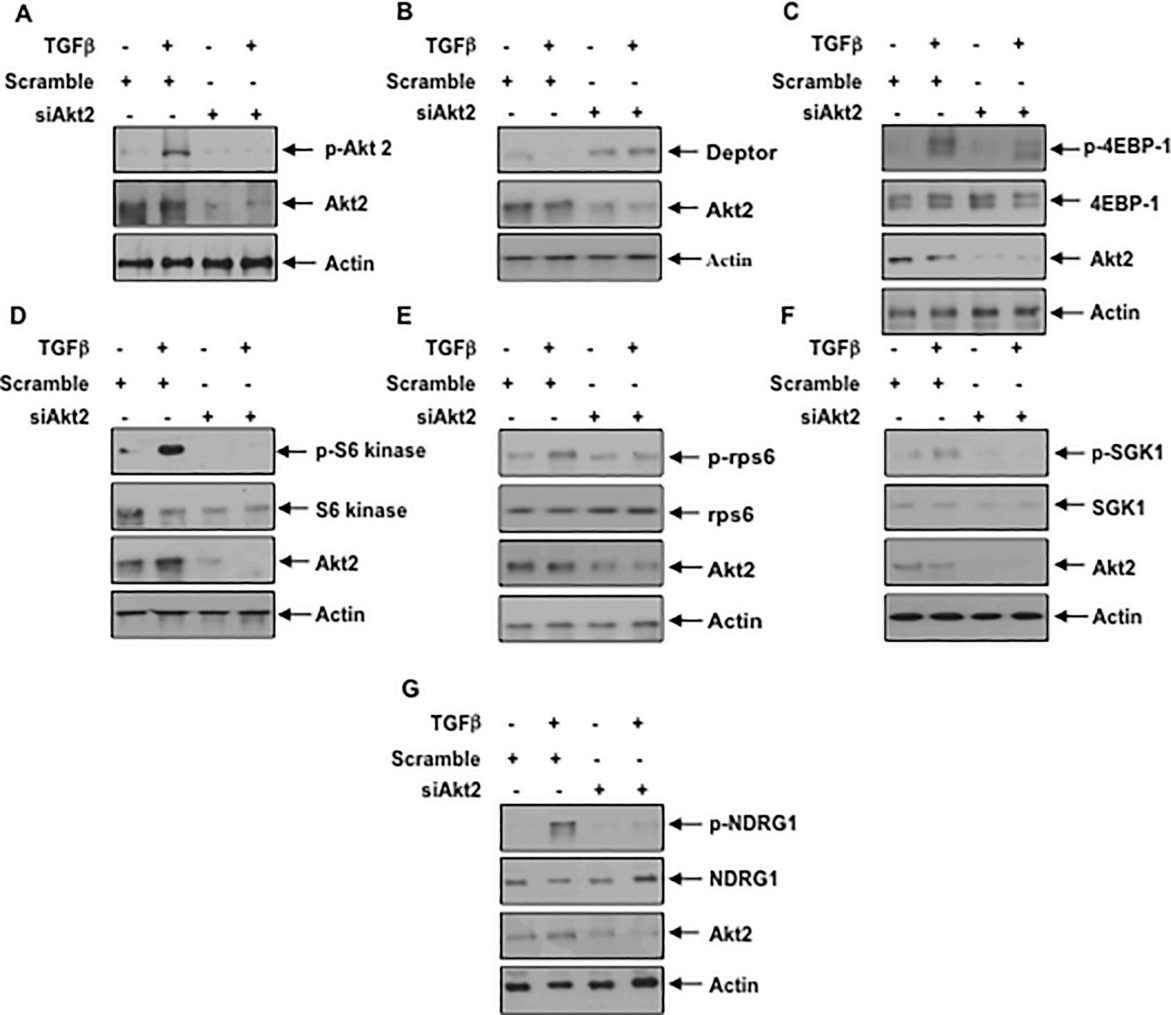
siRNA against Akt2 blocks TGFβ induced suppression of deptor and activation of mTORC1 and mTORC2. (A–G) Podocytes were transfected with scramble RNA and siRNA against Akt2. The transfected cells were starved and treated with TGFβ The cell lysates were immunoblotted with indicated antibodies. Representative of 4 independent experiments is shown. The quantification is shown in the S10 Fig.

Akt2 controls deptor expression: We have shown above that inhibition of PI 3 kinase and Akt blocks the TGFβ induced suppression of deptor (Figs 2A, 3A and 4A). Since Akt2 is the predominant isoform present in the podocytes, we tested its involvement in the regulation of deptor expression. In fact, MK which prevented deptor downregulation (Fig 4A), inhibited Akt2 phosphorylation by TGFβ S8 Fig). We used a phospho-deficient mutant of Akt2, which acts as the dominant negative kinase [51]. Expression of Akt2 T309A/S474A mutant abrogated the phosphorylation of Akt2 in response to TGFβ resulting in inhibition of downregulation of deptor (Fig 6A and 6B and S9A and S9B Fig). In fact, phospho-deficient mutant of Akt2 suppressed the TGFβ induced activation of mTORC1 (phosphorylation of 4EBP-1/S6 kinase/rps6) and mTORC2 (phosphorylation of SGK1 and NDRG1) (Fig 6C–6G and S9C–S9G Fig). To confirm the role of Akt2, we employed siRNA against this kinase. siRNA against Akt2 showed reduced phosphorylation of this kinase, which reversed the downregulation of deptor induced by TGFβ (Fig 7A and 7B and S10A and S10B Fig). Consequently, siAkt2 inhibited the TGFβ stimulated mTORC1 and mTORC2 activities (Fig 7C–7G and S10C–S10G Fig). These results conclusively demonstrate a significant role of Akt2 in TGFβ induced deptor inhibition and mTOR activation.

**Fig 8:**
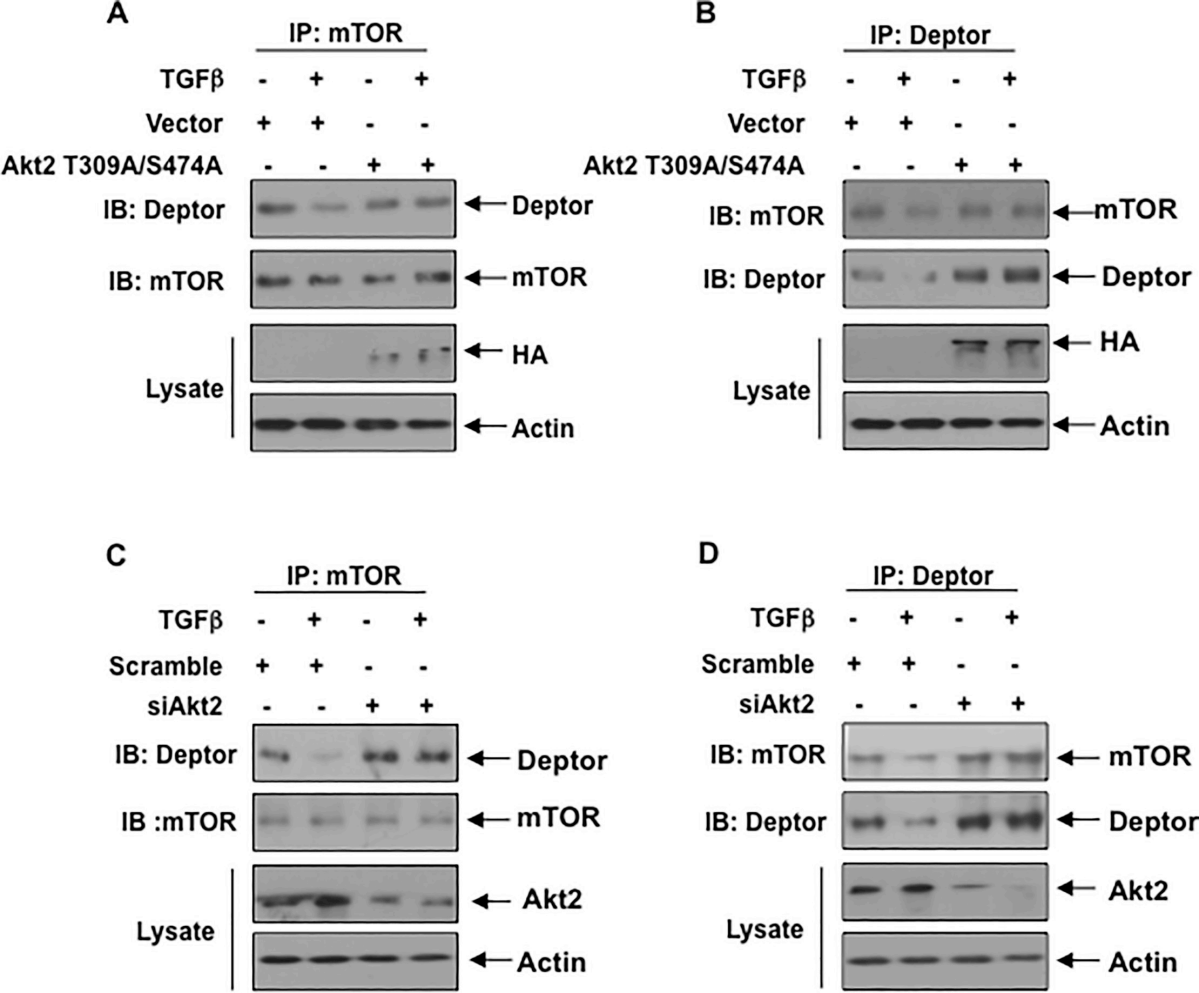
Phospho-deficient mutant of Akt2 and siRNA against Akt2 prevent dissociation of deptor from mTOR by TGFβ (A and B) Podocytes were transfected with Akt2 T309A/S474A mutant plasmid. The transfected cells were starved and incubated with TGFβ The cell lysates were immunoprecipitated with mTOR (panel A) and deptor (panel B). The immunoprecipitates were immunoblotted with deptor and mTOR antibodies as indicated. Also, the total cell lysates were immunoblotted with HA and actin antibodies (bottom two panels). (C and D) The cells were transfected with scramble RNA or siRNA against Akt2. The transfected cells were immunoprecipitated with mTOR (panel C) and deptor (panel D). The immunoprecipitates were immunoblotted with deptor and mTOR antibodies as indicated. Also, the total cell lysates were immunoblotted with Akt2 and actin antibodies (bottom two panels). Representative of 3 independent experiments is shown. The quantification is shown in the S11 Fig.

Akt2 regulates association of deptor with mTOR: Deptor is an mTOR interacting protein and is present in both mTORC1 and mTORC2. Deptor inhibits the activity of both these complexes [17]. We determined the association of deptor with mTOR. Coimmunoprecipitation and reciprocal coimmunoprecipitation experiments showed a significant decrease in association of deptor and mTOR in response to TGFβ (Fig 8). Next, we examined the role of Akt2 in the complex formation between deptor and mTOR. First, we used the phospho-deficient mutant of Akt2. Expression of this mutant reversed the TGFβ mediated inhibition of association of deptor with the mTOR (Fig 8A and 8B and S11A and S11B Fig). Similarly, siRNAs against Akt2 inhibited the dissociation of deptor from mTOR by TGFβ (Fig 8C and 8D and S11C and S11D Fig). These data demonstrate that downregulation of deptor by TGFβ contributes to reduced association of these two proteins. Furthermore, our results for the first time show a significant role of Akt2 in not only inhibiting the deptor levels, but affecting the consequent association between mTOR and deptor.

**Fig 9:**
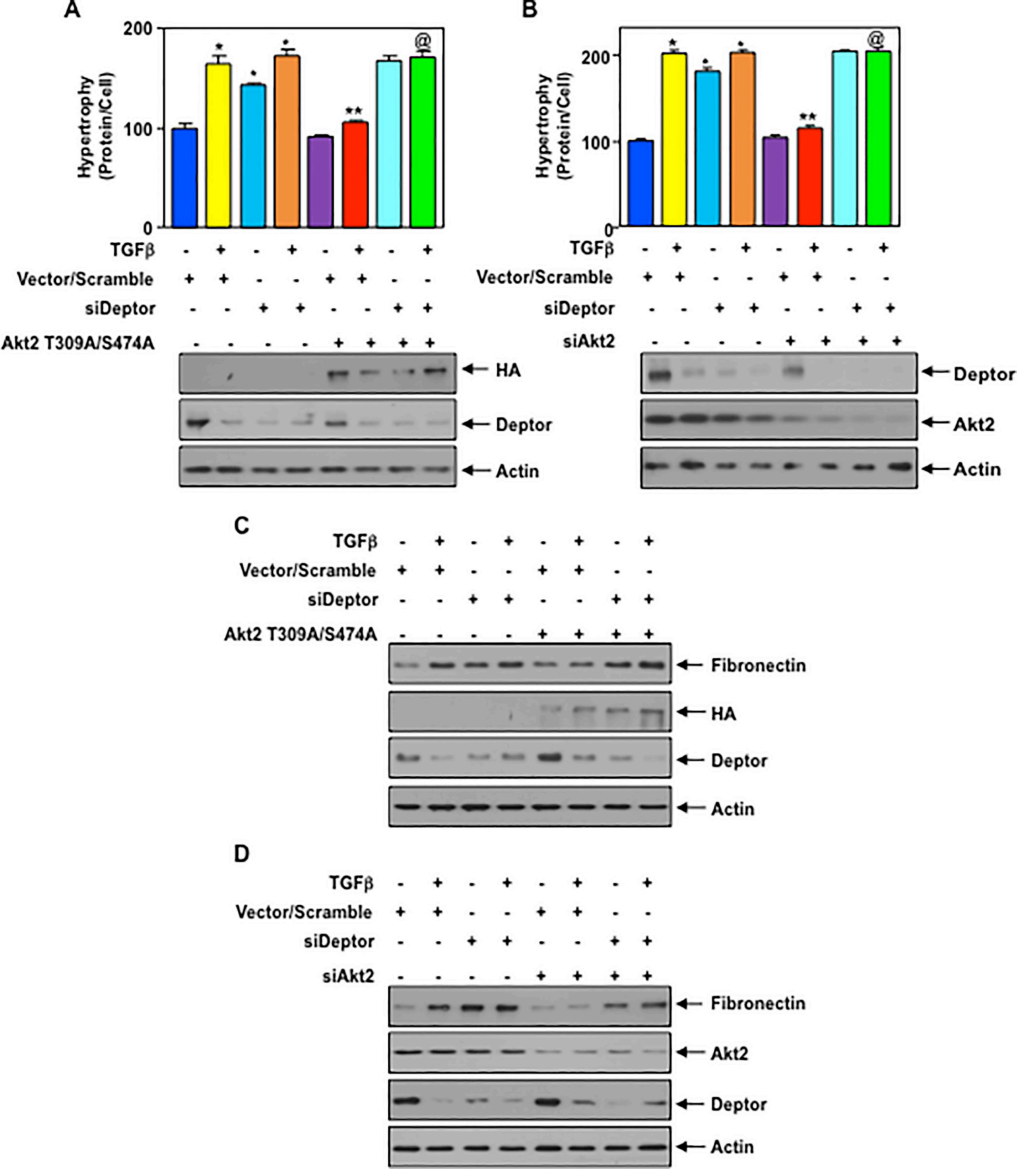
Phospho-deficient mutant of Akt2 and siRNA against Akt2 block TGFβ induced podocyte hypertrophy and expression of fibronectin by deptor. (A—D) Podocytes were transfected with Akt2 T309A/S474A mutant plasmid (panels A and C) or siRNA against Akt2 (panels B and D) along with siRNA for deptor. The transfected cells were starved and incubated with TGFβ In panels A and B, the hypertrophy of the cells was determined as described in the Materials and Methods. Mean ± SE of triplicate measurements is shown. *, vs control; **, vs TGFβ @, vs TGFβ plus Akt2T309A/S474A. p < 0.001. Bottom panels show the expression of Akt2 mutant and deptor along with actin. (C and D) The cell lysates were immunoblotted with indicated antibodies. Representative of 3 independent experiments is shown. The quantification of fibronectin expression in Fig 9C and 9D is shown in the S12 Fig.

Akt2 controls podocyte hypertrophy and matrix protein expression via deptor: Podocyte hypertrophy and matrix protein synthesis contribute to glomerulosclerosis in various kidney diseases [1, 3]. First, we examined the role of Akt2 in TGFβ induced podocyte hypertrophy. Expression of phospho-deficient mutant of Akt2 significantly inhibited the hypertrophy of podocytes induced by TGFβ. In contrast, siRNAs against deptor alone or in conjunction with TGFβ induced hypertrophy (Fig 9A).Interestingly, siRNAs against deptor reversed the phospho-deficient Akt2-mediated inhibition of hypertrophy in the presence of TGFβ (Fig 9A). Similarly, siRNAs against Akt2 blocked TGFβ induced hypertrophy and, co-expression of siRNA for deptor prevented this inhibition (Fig 9B). Next, we determined the effect of Akt2 inhibition on matrix protein fibronectin expression in the podocytes. Both phospho-deficient mutant of Akt2 and siRNAs against Akt2 significantly inhibited TGFβ stimulated expression of fibronectin (Fig 9C and 9D and S12A and S12B Fig). siRNAs against deptor was sufficient to increase the expression of this matrix protein similar to that observed with TGFβ treatment alone (Fig 9C and 9D and S12A and S12B Fig). Furthermore, siDeptor reversed the phospho-deficient Akt2 mutant- and siAkt2-mediated inhibition of fibronectin expression in the presence of TGFβ (Fig 9C and 9D and S12A and S12B Fig). These results demonstrate that deptor downstream of Akt2 regulates the podocyte hypertrophy and matrix protein expression in response to TGFβ.

**Fig 10:**
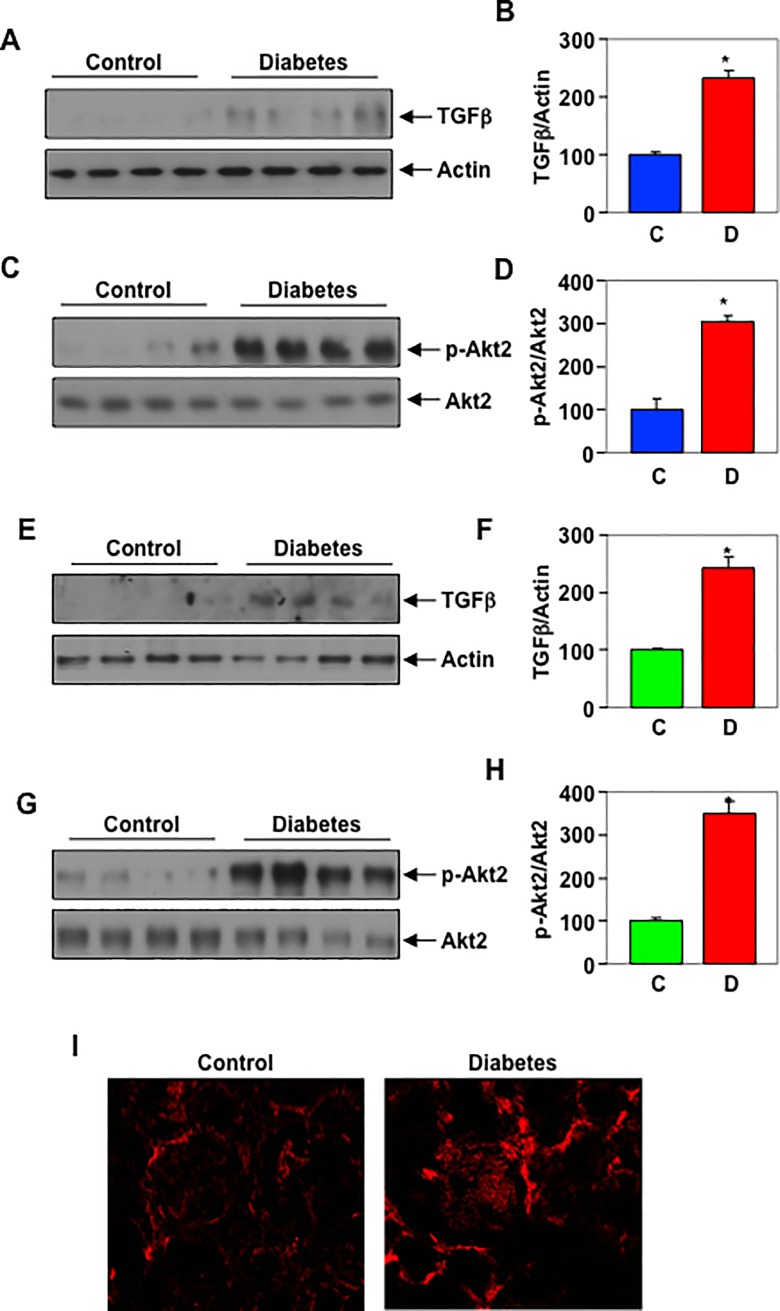
Phosphorylation of Akt2 in the kidneys of STZ-induced diabetic rats. (A and C) Cortical lysates from STZ-induced diabetic rats were immunoblotted with the TGFβ actin, phospho-Akt2 and Akt2 as indicated. Panels B and D show quantification of the TGFβ and phospho-Akt levels. Mean ± SE of 4 animals is shown. *p = 0.003 (panel B) or 0.004 (panel D) vs control. (E and G) Glomerular lysates of diabetic rats were immunoblotted with indicated antibodies. Panels F and H show quantification. Mean ± SE of 4 animals is shown. *p = 0.015 (panel F) or 0.004 (panel H) vs control. I, Immunofluorescence analysis of expression of phospho-Akt2 in the renal glomerulus.

Phosphorylation of Akt2 in the kidneys of STZ-induced diabetic rats: Increased expression of TGFβ with augmented signaling has been shown in the kidneys of patients with diabetic nephropathy [52, 53]. Our results described above show activation of Akt2 in response to TGFβ Also, we showed activation of Akt2 is necessary for the matrix protein fibronectin expression. To assess the *in vivo* relevance of our findings, we determined the phosphorylation of Akt2 in the renal cortex of diabetic rats [54]. The results show a significant increase in the expression of TGFβ in the renal cortex of STZ-induced diabetic rats (Fig 10A and 10B). Importantly, this increase in TGFβ was associated with enhanced phosphorylation of Akt2 in the renal cortex of diabetic rats (Fig 10C and 10D). Similarly, in the glomeruli of diabetic animals, the expression of TGFβ was associated with increased levels of phospho-Akt2 (Fig 10E–10G). Immunofluorescence staining showed increased Akt2 phosphorylation in the glomeruli of diabetic rat as compared to control (Fig 10I, white arrow). These results conclusively demonstrate increased Akt2 signaling in the kidneys of diabetic animals.

## Discussion

**Fig 11:**
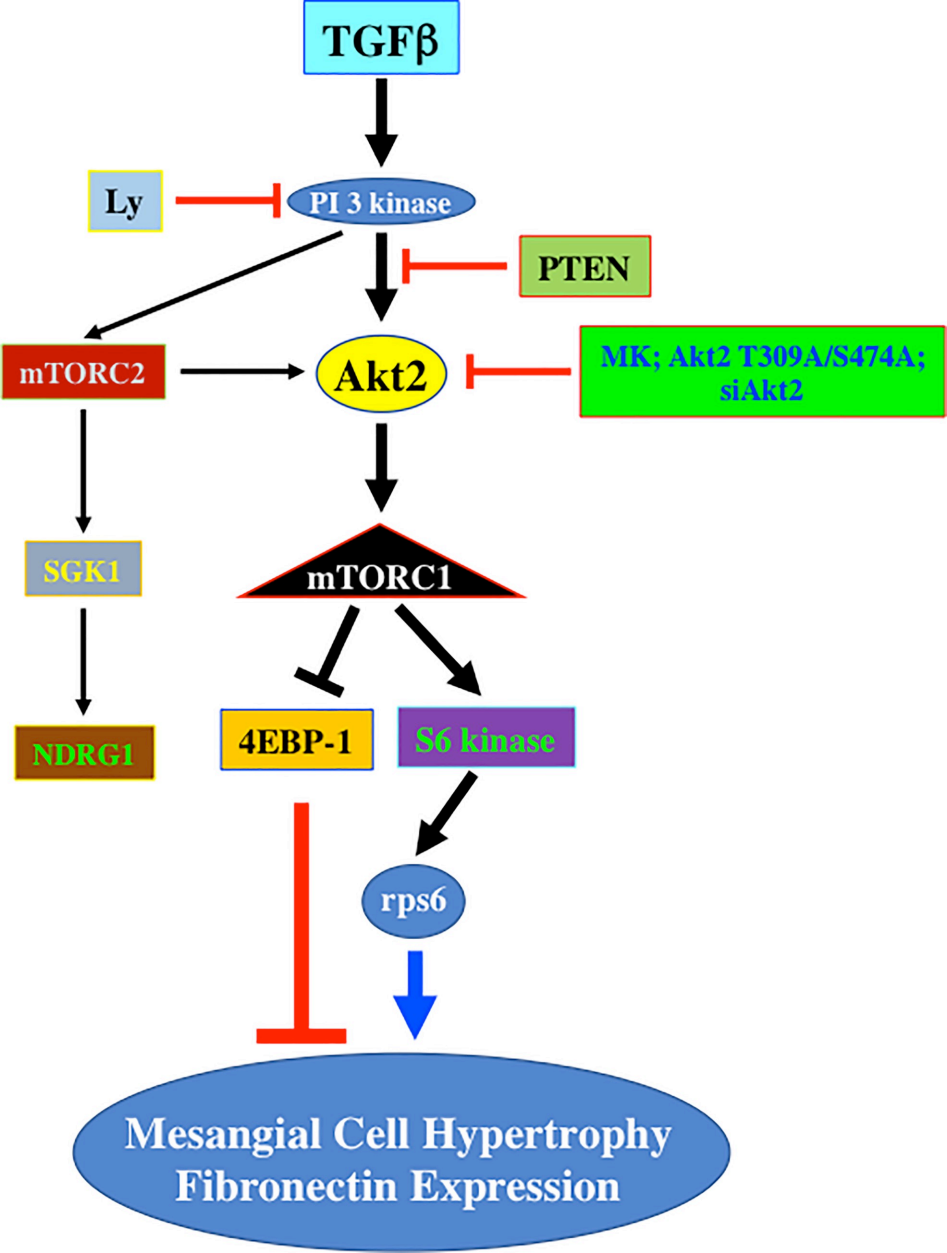
Schematic summary of results presented in this study.

We show for the first time that PI 3 kinase-driven activation of Akt2 isoform controls deptor downregulation by TGFβ, leading to mTORC1/mTORC2 activation, and podocyte hypertrophy and matrix protein accumulation (Fig 11). Furthermore, we demonstrate in the kidneys of diabetic rat that increased Akt2 phosphorylation is associated with expression of TGFβ.

Increased expression of TGFβ contributes to the development of glomerulosclerosis, which results from accumulation of extracellular matrix synthesized by constituent cells including podocytes [9]. For both these pathologies the role of mTORC1 and mTORC2 has been established [32, 55, 56]. In mice with diabetic nephropathy where TGFβ contributes to glomerular hypertrophy and matrix protein expression, we have shown recently that rapamycin ameliorated both these pathologies [57, 58]. However, rapamycin treatment in humans is often associated with proteinuria including in patients with chronic allograft nephropathy and after receiving renal transplantation [59–61]. Also, in animal models of renal insufficiency such as remnant kidney and puromycin amionucleoside-induced nephrotoxicity, rapamycin showed deleterious effect [62, 63]. Mice deficient in mTORC1 in podocyte show pathologic features of human focal and segmental glomerulosclerosis [32]. Thus loss of mTOR activity is detrimental to the normal kidney function. Therefore, an alternative strategy to block hyperactive mTOR in disease conditions is necessary. One possible mechanistic outlook may be to investigate the signal transduction mechanism that can be targeted to increase the expression of the endogenous inhibitor of mTOR, deptor, to block hyperactive mTOR in disease states.

Deptor is expressed differentially in various cancers with opposite functions and can act as oncogene or tumor suppressor [64]. For example, in many cancer cells, deptor level is significantly reduced and consequently both mTORC1 and mTORC2 activities are increased [17, 64, 65]. On the other hand, in multiple myelomas, hepatocellular carcinoma, thyroid carcinoma and osteosarcomas, increased deptor expression suppresses mTORC1 activity, which increases the phosphorylation of Akt by relieving the negative feed-back loop on PI 3 kinase/IRS-1 [17, 66–68]. However, the role of deptor in podocytes does not align with its function found in these cancers. Rather, we show that TGFβ decreased the expression of deptor in podocytes suggesting deptor reduction occurs in the context of podocyte injury induced by the cytokine.

In cancer cells, deptor abundance is regulated by transcriptional and proteasomal degradation pathways [17, 69]. The signal transduction mechanisms that may contribute to the reduced deptor abundance are not yet understood. We previously reported that TGFβ activates PI 3 kinase to increase mTOR activity and matrix protein expression in renal cells [12]. In fact we showed that PI 3 kinase is necessary for the TGFβ induced mTOR activation [13]. Since downregulation of deptor is necessary for mTOR activity, we now demonstrate a significant role of PI 3 kinase in deptor downregulation and its associated prolonged mTORC1 and mTORC2 activation.

TGFβ stimulated PI 3 kinase-produced D3-phosphorylated inositides bind to the PH-domain of Akt kinase to induce its translocation to the cell membranes, where it undergoes activation by phosphorylation at the catalytic loop threonine and hydrophobic motif serine [50, 70]. In mammals, Akt exists in three isotypes with highly conserved structure. Akt1 is ubiquitously expressed in the heart, lung and brain, while Akt2 is restricted to the insulin-sensitive tissues in the skeletal muscle, liver and adipose tissues. In contrast Akt3 is expressed mostly in the brain and testis, mammary glands, pancreas fat and lung [71–73].

Akt1 knock out mice show reduced overall size with severe neonatal mortality [74]. Also, Akt1 deficiency showed its significant role in regulation of cardiovascular function as Akt1 regulates the physiological cardiac hypertrophy such as in exercise-induced cardiac hypertrophy [75]. Interestingly, inducible long-term Akt1 activation in mice displayed pathological hypertrophy [76]. Akt1 regulates platelet aggregation and spreading, which may also contribute to cardiovascular disease [77]. Thus Akt1 plays both positive and negative regulatory roles in cardiovascular disease. In contrast to the Akt1, which controls the size of all organs, Akt3 does not regulate organismal size; rather it contributes to the brain size due to its influence on cell size. [74, 78, 79]. In brain, Akt1 is also abundantly expressed. Similar to Akt3 deficient mice, Akt1 deficiency also exhibits smaller brain size [78]; however, only Akt3 contributes to the activation of mTORC1 [78]. Interestingly, inhibition of expression of Akt1 in the podocyte did not have any effect on TGFβ stimulated mTORC1 and mTORC2 activities (S13A and S13B Fig).

Akt 2 deficient mice display type 2 diabetic syndrome with impaired glucose utilization in muscle and adipose tissues [80, 81]. In fact a dominant negative Akt2 mutation was identified in a family to associate with severe insulin resistance and diabetes suggesting its role in metabolic regulation [82]. A recent study in the kidney showed abundant expression of Akt1 in the tubular compartment of the kidney while Akt2 was localized to the glomeruli [83]. However, in the rat renal cortex which contains more than 90% tubules and in renal glomeruli, which contains podocyte as one of the three cell types, we detected significantly high levels of Akt2 although low expression of both Akt1 and Akt3 was also seen. Similar expression profile of Akt isoforms was observed in the podocytes. In fact, using an allosteric inhibitor MK2206, which blocks the kinase activity of all isoforms of Akt prevented the TGFβ induced downregulation of deptor. However, TGFβ stimulated PI 3 kinase significantly increased the phosphorylation of Akt2, the predominant isoform in the podocytes. Furthermore, we provide evidence that Akt2 contributes to the TGFβ induced downregulation of deptor. To our knowledge, this is the first demonstration of an Akt isoform, which regulates the expression of deptor.

A role for PI 3 kinase/Akt signaling in organismal hypertrophy has been reported. First indication came from the observation that transgenic expression of the catalytic subunit of PI 3 kinase in eye and wing increased the organ size in *Drosophila* [84]. Similarly, deletion of PTEN, which increases PI 3 kinase/Akt kinase signaling, in *Drosophila* eye and wing discs increased the cell size that lead to enlarged organs [85, 86]. Similar results were obtained when constitutively active Akt was expressed in the imaginal discs of the fruit fly [87]. In the mouse heart, overexpression of constitutively active catalytic subunit of PI 3 kinase or Akt or deletion of PTEN yielded increase in heart size [88–90]. In line with these results, we showed the involvement of PI 3 kinase/Akt signaling in renal hypertrophy in rodent models of diabetic kidney disease where TGFβ contributes to the pathology. We also demonstrated a role of Akt kinase in matrix protein expression by TGFβ [12, 36, 42]. In renal fibrosis such as in diabetic nephropathy, increased levels of TGFβ has been shown to play a significant role by activating Akt [42, 43, 91, 92]. Interestingly, we observed increased phosphorylation of Akt2 in the glomeruli of rats with type 1 diabetes.

Our results indicate a role of PI 3 kinase in deptor downregulation to induce podocyte hypertrophy and fibronectin expression. In fact, using dominant negative and siRNA methods, we show for the first time that activation of specific isoform of Akt, Akt2, by TGFβ contributes to the activation of both mTORC1 and mTORC2 via deptor downregulation. In summary, our results provide the first mechanistic view for involvement of Akt2 in TGFβ stimulated podocyte hypertrophy and matrix protein accumulation seen in glomerulosclerosis.

## Supporting information

S1 Fig Expression of podocyte-specific markers. Rat podocytes were lysed in RIPA buffer. Equal amounts of protein were immunoblotted with synaptopodin (panel A), nephrin (panel B) and actin antibodies. Glomerular mesangial cell lysates were used as negative control to show the specificity of the expression of these proteins in podocyte only.
(TIF)

S2 Fig Quantification of the results shown in Fig 1A–1F. (A) Ratio of deptor to actin. Mean ± SE of 3 independent experiments is shown. *p < 0.001 vs 0 hour. (B) Ratio of phospho-S6 kinase to S6 kinase. Mean ± SE of 3 independent experiments is shown. *p < 0.001 vs 0 hour. (C) Ratio of phospho-4EBP-1 to 4EBP-1. Mean ± SE of 3 independent experiments is shown. *p < 0.001 vs 0 hour. (D) Ratio of phospho-SGK1 to SGK1. Mean ± SE of 3 independent experiments is shown. *p < 0.001 vs 0 hour. (E) Ratio of phospho-rps6 to rps6. Mean ± SE of 3 independent experiments is shown. *p < 0.05 vs 0 hour. (F) Ratio of phospho-NDRG1 to NDRG1. Mean ± SE of 3 independent experiments is shown. *p < 0.001 vs 0 hour.
(TIF)

S3 Fig Quantification of the results shown in Fig 2A–2L. (A) Ratio of deptor to actin. Mean ± SE of 4 independent experiments is shown. *p < 0.001 vs control; **p < 0.001 vs TGFβ alone. (B) Ratio of phospho-4EBP-1 to 4EBP-1. Mean ± SE of 4 independent experiments is shown. *p < 0.001 vs control; **p < 0.001 vs TGFβ alone. (C) Ratio of phospho-S6 kinase to S6 kinase. Mean ± SE of 4 independent experiments is shown. *p < 0.001 vs control; **p < 0.001 vs TGFβ alone. (D) Ratio of phospho-rps6 to rps6. Mean ± SE of 4 independent experiments is shown. *p < 0.001 vs control; **p < 0.001 vs TGFβ alone. (E) Ratio of phospho-SGK1 to SGK1. Mean ± SE of 4 independent experiments is shown. *p < 0.001 vs control; **p < 0.001 vs TGFβ alone. (F) Ratio of phospho-NDRG1 to NDRG1. Mean ± SE of 4 independent experiments is shown. *p < 0.01 vs control; **p < 0.01 vs TGFβ alone. (G) Ratio of deptor to actin. Mean ± SE of 4 independent experiments is shown. *p < 0.001 vs control; **p < 0.001 vs TGFβ alone. (H) Ratio of phospho-4EBP-1 to 4EBP-1. Mean ± SE of 4 independent experiments is shown. *p < 0.001 vs control; **p < 0.001 TGFβ alone. (I) Ratio of phospho-S6 kinase to S6 kinase. Mean ± SE of 4 independent experiments is shown. *p < 0.001 vs control; **p < 0.001 vs TGFβ alone. (J) Ratio of phospho-rps6 to rps6. Mean ± SE of 4 independent experiments is shown. *p < 0.001 vs control; **p < 0.001 vs TGFβ alone. (K) Ratio of phospho-SGK1 to SGK1. Mean ± SE of 4 independent experiments is shown. *p < 0.01 vs control; **p < 0.001 vs TGFβ alone. (L) Ratio of phospho-NDRG1 to NDRG1. Mean ± SE of 4 independent experiments is shown. *p < 0.01 vs control; **p < 0.001 vs TGFβ alone.
(TIF)

S4 Fig Quantification of the results shown in Fig 3A–3F. (A) Ratio of deptor to actin. Mean ± SE of 4 independent experiments is shown. *p < 0.001 vs control; **p < 0.001 vs TGFβ alone. (B) Ratio of phospho-4EBP-1 to 4EBP-1. Mean ± SE of 4 independent experiments is shown. *p < 0.001 vs control; **p < 0.001 vs TGFβ alone. (C) Ratio of phospho-S6 kinase to S6 kinase. Mean ± SE of 4 independent experiments is shown. *p < 0.001 vs control; **p < 0.001 vs TGFβ alone. (D) Ratio of phospho-rps6 to rps6. Mean ± SE of 4 independent experiments is shown. *p < 0.001 vs control; **p < 0.001 vs TGFβ alone. (E) Ratio of phospho-SGK1 to SGK1. Mean ± SE of 4 independent experiments is shown. *p < 0.001 vs control; **p < 0.001 vs TGFβ alone. (F) Ratio of phospho-NDRG1 to NDRG1. Mean ± SE of 4 independent experiments is shown. *p < 0.001 vs control; **p < 0.001 vs TGFβ alone.
(TIF)

S5 Fig Quantification of the results shown in Fig 4A–4F. (A) Ratio of deptor to actin. Mean ± SE of 4 independent experiments is shown. *p < 0.001 vs control; **p < 0.001 vs TGFβ alone. (B) Ratio of phospho-4EBP-1 to 4EBP-1. Mean ± SE of 4 independent experiments is shown. *p < 0.001 vs control; **p < 0.001 vs TGFβ alone. (C) Ratio of phospho-S6 kinase to S6 kinase. Mean ± SE of 4 independent experiments is shown. *p < 0.001 vs control; **p < 0.001 vs TGFβ alone. (D) Ratio of phospho-rps6 to rps6. Mean ± SE of 4 independent experiments is shown. *p < 0.001 vs control; **p < 0.001 vs TGFβ alone. (E) Ratio of phospho-SGK1 to SGK1. Mean ± SE of 4 independent experiments is shown. *p < 0.001 vs control; **p < 0.001 vs TGFβ alone. (F) Ratio of phospho-NDRG1 to NDRG1. Mean ± SE of 4 independent experiments is shown. *p < 0.001 vs control; **p < 0.001 vs TGFβ alone.
(TIF)

S6 Fig Expression of Akt isoforms in the rat kidney. Renal cortex (panel A) and glomeruli (panel B) were lysed in RIPA buffer. The extracts from three independent animals were immunoblotted with isotype-specific antibodies against Akt1, Ak2 and Akt3. Level of actin was assessed as loading control. Right part of each panel shows quantification of isoform expression. *p < 0.0001 vs Akt1 or Akt3.
(TIF)

S7 Fig Quantification of the results shown in Fig 5A–5E. (A) Quantification of Akt isoform levels. Mean ± SE of 3 measurements is shown. *p < 0.0001 vs Akt1 or Akt3. (B) Ratio of phospho-Akt2 to Akt2. Mean ± SE of 3 experiments is shown. *p < 0.001 vs 0 hour. (C—E) Ratio of phospho-Akt2 to Akt2. Mean ± SE of 4 experiments is shown. *p < 0.001 vs control; **p < 0.001 vs TGFβ.
(TIF)

S8 Fig MK blocks TGFβ -induced Akt2 phosphorylation. Starved podocytes were treated with 1 mcromolar MK prior to incubation with 2 ng/ml TGFβ for 24 hours. The cell lysates were immunoblotted with phospho-Akt2 (Ser-474) and Akt2 antibodies. Bottom part shows quantification. Mean ± SE of 3 independent experiments is shown. *p < 0.05 vs control; **p < 0.05 vs TGFβ alone.
(TIF)

S9 Fig Quantification of the results shown in Fig 6A–6G. (A) Ratio of phospho-Akt2 to Akt2. Mean ± SE of 4 independent experiments is shown. *p < 0.001 vs control; **p < TGFβ alone. (B) Ratio of deptor to actin. Mean ± SE of 4 independent experiments is shown. *p < 0.001 vs control; **p < TGFβ alone. (C) Ratio of 4EBP-1 to 4EBP-1. Mean ± SE of 4 independent experiments is shown. *p < 0.001 vs control; **p < TGFβ alone. (D) Ratio of phospho-S6 kinase to S6 kinase. Mean ± SE of 4 independent experiments is shown. *p < 0.001 vs control; **p < TGFβ alone. (E) Ratio of phospho-rps6 to rps6. Mean ± SE of 4 independent experiments is shown. *p < 0.001 vs control; **p < TGFβ alone. (F) Ratio of phospho-SGK1 to SGK1. Mean ± SE of 4 independent experiments is shown. *p < 0.001 vs control; **p < TGFβ alone. (G) Ratio of phospho-NDRG1 to NDRG1. Mean ± SE of 4 independent experiments is shown. *p < 0.001 vs control; **p < TGFβ alone.
(TIF)

S10 Fig Quantification of the results shown in Fig 7A–7G. (A) Ratio of phospho-Akt2 to actin. Mean ± SE of 4 independent experiments is shown. *p < 0.001 vs control; **p < TGFβ alone. (B) Ratio of deptor to actin. Mean ± SE of 4 independent experiments is shown. *p < 0.001 vs control; **p < TGFβ alone. (C) Ratio of 4EBP-1 to 4EBP-1. Mean ± SE of 4 independent experiments is shown. *p < 0.001 vs control; **p < TGFβ alone. (D) Ratio of phospho-S6 kinase to S6 kinase. Mean ± SE of 4 independent experiments is shown. *p < 0.001 vs control; **p < TGFβ alone. (E) Ratio of phospho-rps6 to rps6. Mean ± SE of 4 independent experiments is shown. *p < 0.001 vs control; **p < 0.01 vs TGFβ alone. (E) Ratio of phospho-rps6 to rps6. Mean ± SE of 4 independent experiments is shown. *p < 0.001 vs control; **p vs TGFβ alone. (F) Ratio of phospho-SGK1 to SGK1. Mean ± SE of 4 independent experiments is shown. *p < 0.001 vs control; **p vs TGFβ alone. (G) Ratio of phospho-NDRG1 to NDRG1. Mean ± SE of 4 independent experiments is shown. *p < 0.001 vs control; **p < TGFβ alone.
(TIF)

S11 Fig Quantification of the results shown in Fig 8A–8D. (A and C) Ratio of Deptor to mTOR. (B and D) Ratio of mTOR to actin. Mean ± SE of 3 independent experiments is shown. *p < 0.001 vs control; **p < 0.001 vs TGFβ.
(TIF)

S12 Fig (A and B) Quantification of fibronectin expression shown in Fig 9C and 9D respectively. Ratio of fibronectin to actin. Mean ± SE of 3 independent experiments is shown. In panel A, *p < 0.05 vs control; **p < 0.05 vs TGFβ alone; @p < 0.05 vs TGFβ plus Akt2 T309A/S474A. In panel B, *p < 0.001 vs control; **p < 0.001 vs TGFβ alone; @p < 0.001 vs TGFβ plus siAkt2.
(TIF)

S13 Fig Akt1 does not regulate TGFβ -induced activation of mTORC1 and mTORC2. Podocytes were transfected with siRNA against Akt1. The transfected cells were incubated with 2 ng /ml TGFβ for 24 hours. The cell lysates were immunoblotted with phospho-S6 kinase (Thr-389) and phospho-rps6 (Ser-240/244) antibodies to detect mTORC1 activity (panel A). In panel B, the cell lysates were immunoblotted with phospho-Akt2 (Ser-474) antibody to determine mTORC2 activity [50]. For control, S6 kinase, rps6, Akt1, Akt2 and actin antibodies were used as indicated. The right part of each panel shows quantification of the data. Mean ± SE of 3 independent experiments is shown. There was no significant difference between TGFβ and TGFβ plus siAkt1.
(TIF)

S1 Data Raw data for all the Western blots included in the paper. (PDF)
