# Supplementary figures and images for: Akt2 causes TGFβ-induced deptor downregulation facilitating mTOR to drive podocyte hypertrophy and matrix protein expression
Source: PLoS One. 2018 Nov 16;13(11):e0207285. doi: 10.1371/journal.pone.0207285 (PMC6239304; doi:10.1371/journal.pone.0207285)

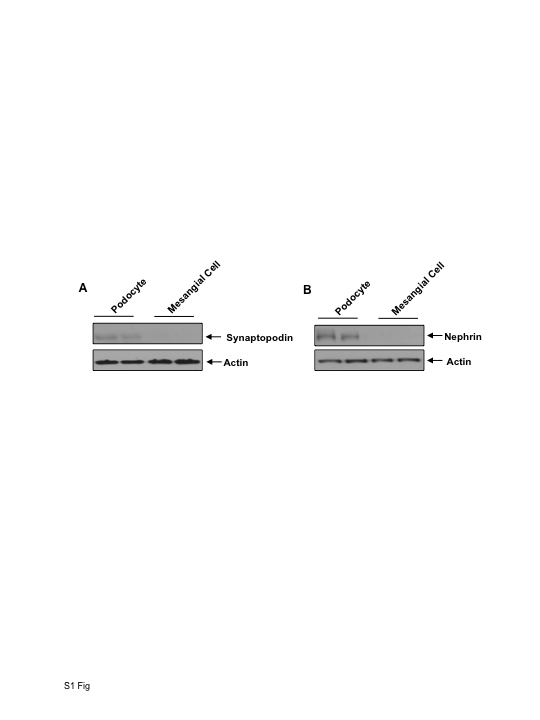

Supplement: S1 Fig — Rat podocytes were lysed in RIPA buffer. Equal amounts of protein were immunoblotted with synaptopodin (panel A), nephrin (panel B) and actin antibodies. Glomerular mesangial cell lysates were used as negative control to show the specificity of the expression of these proteins in podocyte only. (TIF) [file pone.0207285.s001.tif]

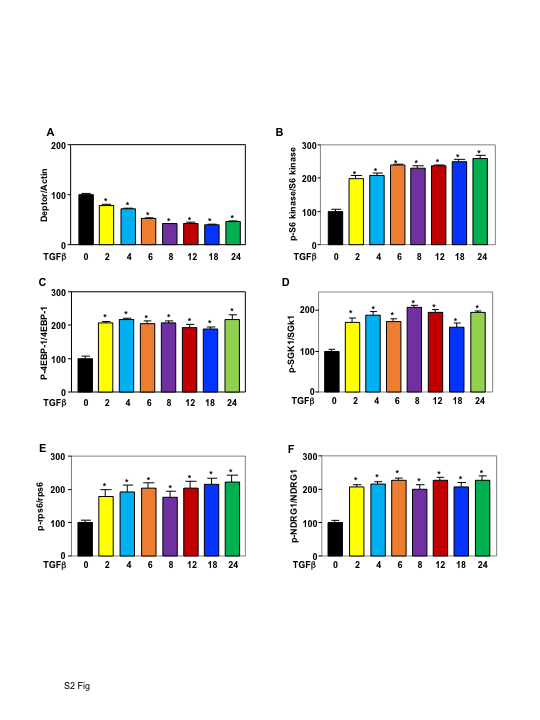

Supplement: S2 Fig — (A) Ratio of deptor to actin. Mean ± SE of 3 independent experiments is shown. *p < 0.001 vs 0 hour. (B) Ratio of phospho-S6 kinase to S6 kinase. Mean ± SE of 3 independent experiments is shown. *p < 0.001 vs 0 hour. (C) Ratio of phospho-4EBP-1 to 4EBP-1. Mean ± SE of 3 independent experiments is shown. *p < 0.001 vs 0 hour. (D) Ratio of phospho-SGK1 to SGK1. Mean ± SE of 3 independent experiments is shown. *p < 0.001 vs 0 hour. (E) Ratio of phospho-rps6 to rps6. Mean ± SE of 3 independent experiments is shown. *p < 0.05 vs 0 hour. (F) Ratio of phospho-NDRG1 to NDRG1. Mean ± SE of 3 independent experiments is shown. *p < 0.001 vs 0 hour. (TIF) [file pone.0207285.s002.tif]

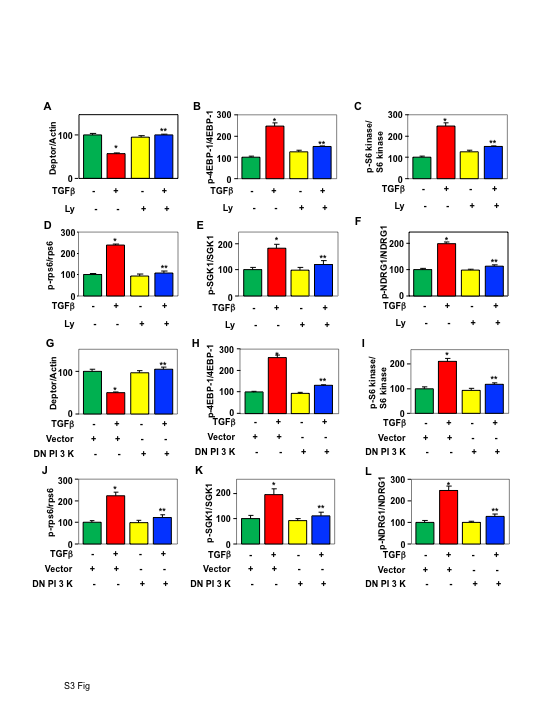

Supplement: S3 Fig — (A) Ratio of deptor to actin. Mean ± SE of 4 independent experiments is shown. *p < 0.001 vs control; **p < 0.001 vs TGFβ alone. (B) Ratio of phospho-4EBP-1 to 4EBP-1. Mean ± SE of 4 independent experiments is shown. *p < 0.001 vs control; **p < 0.001 vs TGFβ alone. (C) Ratio of phospho-S6 kinase to S6 kinase. Mean ± SE of 4 independent experiments is shown. *p < 0.001 vs control; **p < 0.001 vs TGFβ alone. (D) Ratio of phospho-rps6 to rps6. Mean ± SE of 4 independent experiments is shown. *p < 0.001 vs control; **p < 0.001 vs TGFβ alone. (E) Ratio of phospho-SGK1 to SGK1. Mean ± SE of 4 independent experiments is shown. *p < 0.001 vs control; **p < 0.001 vs TGFβ alone. (F) Ratio of phospho-NDRG1 to NDRG1. Mean ± SE of 4 independent experiments is shown. *p < 0.01 vs control; **p < 0.01 vs TGFβ alone. (G) Ratio of deptor to actin. Mean ± SE of 4 independent experiments is shown. *p < 0.001 vs control; **p < 0.001 vs TGFβ alone. (H) Ratio of phospho-4EBP-1 to 4EBP-1. Mean ± SE of 4 independent experiments is shown. *p < 0.001 vs control; **p < 0.001 TGFβ alone. (I) Ratio of phospho-S6 kinase to S6 kinase. Mean ± SE of 4 independent experiments is shown. *p < 0.001 vs control; **p < 0.001 vs TGFβ alone. (J) Ratio of phospho-rps6 to rps6. Mean ± SE of 4 independent experiments is shown. *p < 0.001 vs control; **p < 0.001 vs TGFβ alone. (K) Ratio of phospho-SGK1 to SGK1. Mean ± SE of 4 independent experiments is shown. *p < 0.01 vs control; **p < 0.001 vs TGFβ alone. (L) Ratio of phospho-NDRG1 to NDRG1. Mean ± SE of 4 independent experiments is shown. *p < 0.01 vs control; **p < 0.001 vs TGFβ alone. (TIF) [file pone.0207285.s003.tif]

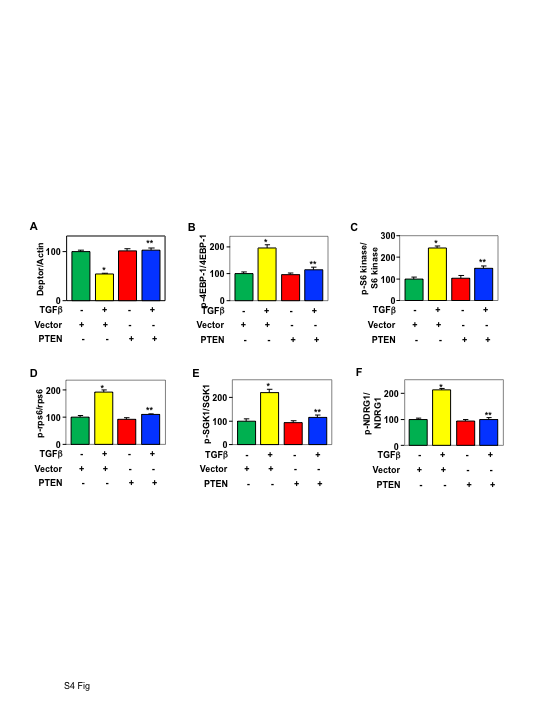

Supplement: S4 Fig — (A) Ratio of deptor to actin. Mean ± SE of 4 independent experiments is shown. *p < 0.001 vs control; **p < 0.001 vs TGFβ alone. (B) Ratio of phospho-4EBP-1 to 4EBP-1. Mean ± SE of 4 independent experiments is shown. *p < 0.001 vs control; **p < 0.001 vs TGFβ alone. (C) Ratio of phospho-S6 kinase to S6 kinase. Mean ± SE of 4 independent experiments is shown. *p < 0.001 vs control; **p < 0.001 vs TGFβ alone. (D) Ratio of phospho-rps6 to rps6. Mean ± SE of 4 independent experiments is shown. *p < 0.001 vs control; **p < 0.001 vs TGFβ alone. (E) Ratio of phospho-SGK1 to SGK1. Mean ± SE of 4 independent experiments is shown. *p < 0.001 vs control; **p < 0.001 vs TGFβ alone. (F) Ratio of phospho-NDRG1 to NDRG1. Mean ± SE of 4 independent experiments is shown. *p < 0.001 vs control; **p < 0.001 vs TGFβ alone. (TIF) [file pone.0207285.s004.tif]

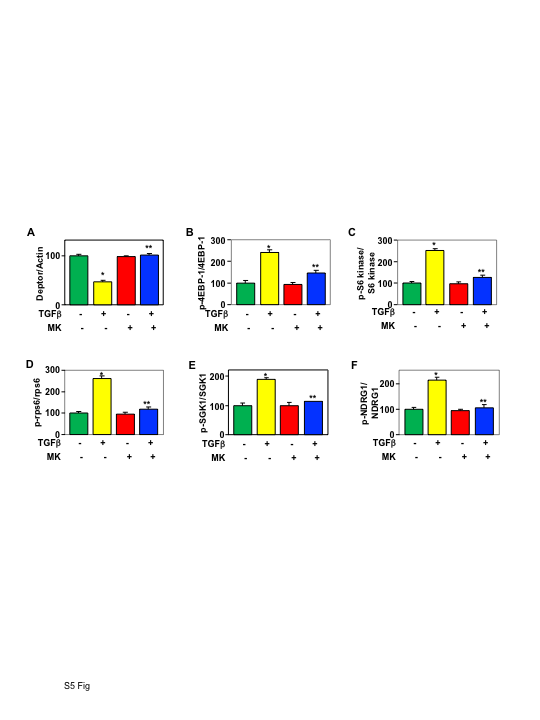

Supplement: S5 Fig — (A) Ratio of deptor to actin. Mean ± SE of 4 independent experiments is shown. *p < 0.001 vs control; **p < 0.001 vs TGFβ alone. (B) Ratio of phospho-4EBP-1 to 4EBP-1. Mean ± SE of 4 independent experiments is shown. *p < 0.001 vs control; **p < 0.001 vs TGFβ alone. (C) Ratio of phospho-S6 kinase to S6 kinase. Mean ± SE of 4 independent experiments is shown. *p < 0.001 vs control; **p < 0.001 vs TGFβ alone. (D) Ratio of phospho-rps6 to rps6. Mean ± SE of 4 independent experiments is shown. *p < 0.001 vs control; **p < 0.001 vs TGFβ alone. (E) Ratio of phospho-SGK1 to SGK1. Mean ± SE of 4 independent experiments is shown. *p < 0.001 vs control; **p < 0.001 vs TGFβ alone. (F) Ratio of phospho-NDRG1 to NDRG1. Mean ± SE of 4 independent experiments is shown. *p < 0.001 vs control; **p < 0.001 vs TGFβ alone. (TIF) [file pone.0207285.s005.tif]

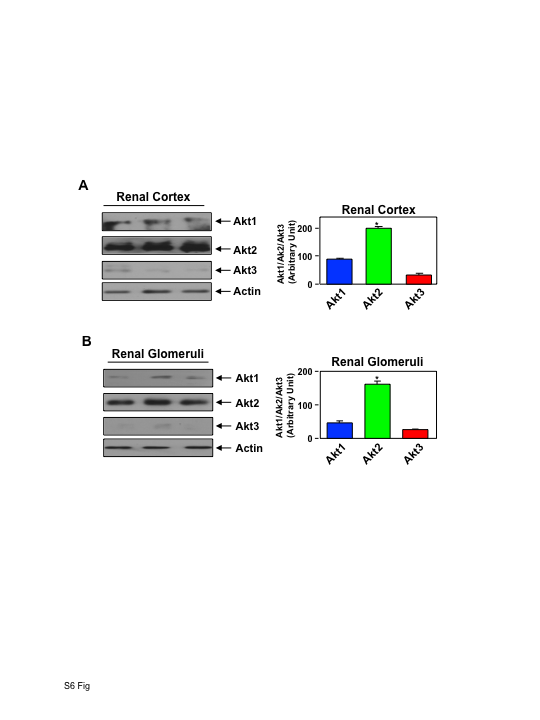

Supplement: S6 Fig — Renal cortex (panel A) and glomeruli (panel B) were lysed in RIPA buffer. The extracts from three independent animals were immunoblotted with isotype-specific antibodies against Akt1, Ak2 and Akt3. Level of actin was assessed as loading control. Right part of each panel shows quantification of isoform expression. *p < 0.0001 vs Akt1 or Akt3. (TIF) [file pone.0207285.s006.tif]

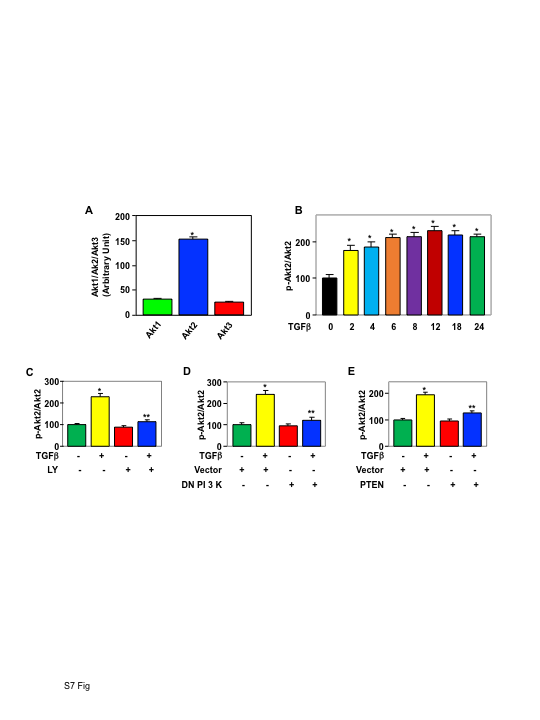

Supplement: S7 Fig — (A) Quantification of Akt isoform levels. Mean ± SE of 3 measurements is shown. *p < 0.0001 vs Akt1 or Akt3. (B) Ratio of phospho-Akt2 to Akt2. Mean ± SE of 3 experiments is shown. *p < 0.001 vs 0 hour. (C—E) Ratio of phospho-Akt2 to Akt2. Mean ± SE of 4 experiments is shown. *p < 0.001 vs control; **p < 0.001 vs TGFβ. (TIF) [file pone.0207285.s007.tif]

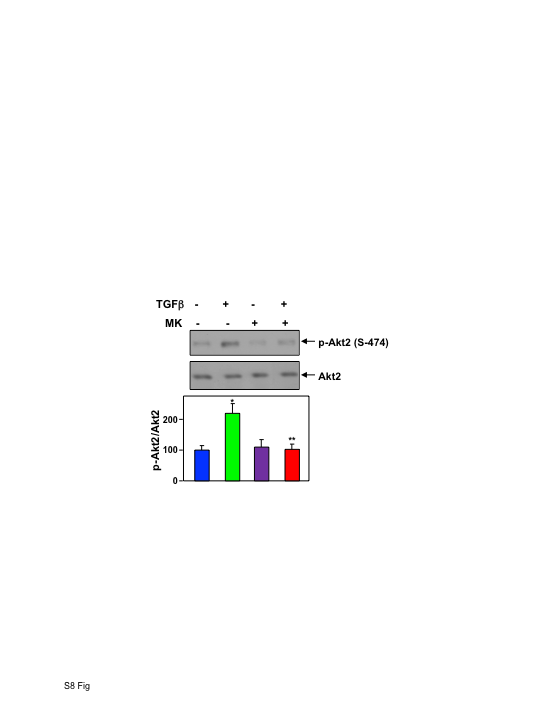

Supplement: S8 Fig — Starved podocytes were treated with 1 mcromolar MK prior to incubation with 2 ng/ml TGFβ for 24 hours. The cell lysates were immunoblotted with phospho-Akt2 (Ser-474) and Akt2 antibodies. Bottom part shows quantification. Mean ± SE of 3 independent experiments is shown. *p < 0.05 vs control; **p < 0.05 vs TGFβ alone. (TIF) [file pone.0207285.s008.tif]

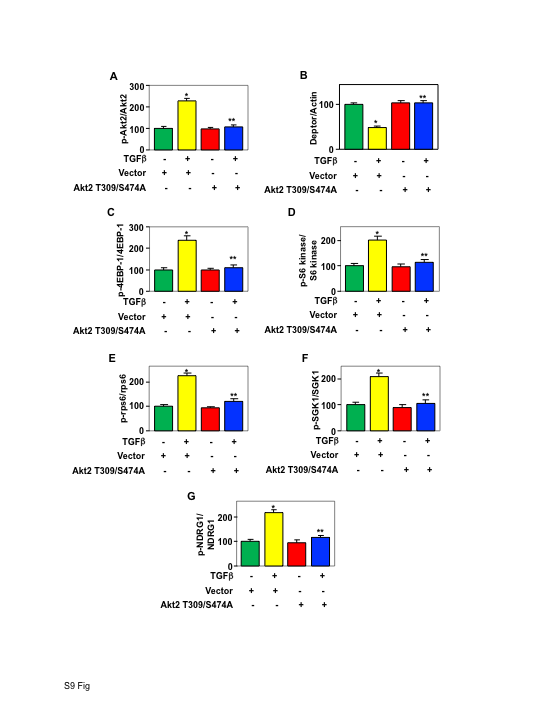

Supplement: S9 Fig — (A) Ratio of phospho-Akt2 to Akt2. Mean ± SE of 4 independent experiments is shown. *p < 0.001 vs control; **p < TGFβ alone. (B) Ratio of deptor to actin. Mean ± SE of 4 independent experiments is shown. *p < 0.001 vs control; **p < TGFβ alone. (C) Ratio of 4EBP-1 to 4EBP-1. Mean ± SE of 4 independent experiments is shown. *p < 0.001 vs control; **p < TGFβ alone. (D) Ratio of phospho-S6 kinase to S6 kinase. Mean ± SE of 4 independent experiments is shown. *p < 0.001 vs control; **p < TGFβ alone. (E) Ratio of phospho-rps6 to rps6. Mean ± SE of 4 independent experiments is shown. *p < 0.001 vs control; **p < TGFβ alone. (F) Ratio of phospho-SGK1 to SGK1. Mean ± SE of 4 independent experiments is shown. *p < 0.001 vs control; **p < TGFβ alone. (G) Ratio of phospho-NDRG1 to NDRG1. Mean ± SE of 4 independent experiments is shown. *p < 0.001 vs control; **p < TGFβ alone. (TIF) [file pone.0207285.s009.tif]

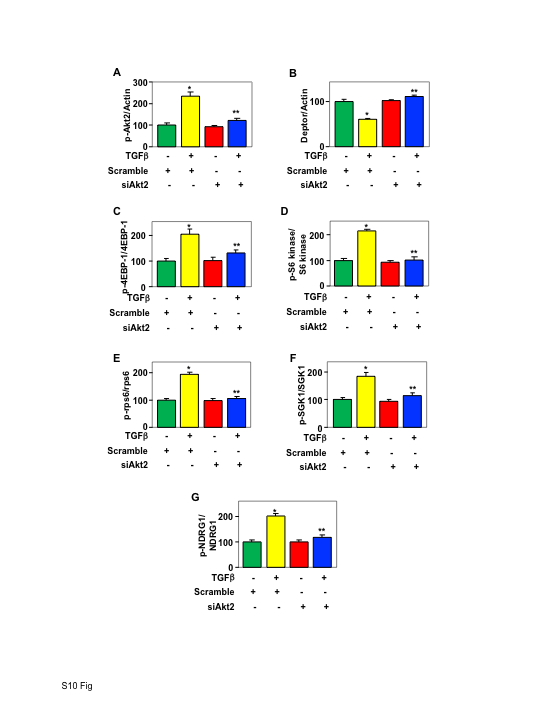

Supplement: S10 Fig — (A) Ratio of phospho-Akt2 to actin. Mean ± SE of 4 independent experiments is shown. *p < 0.001 vs control; **p < TGFβ alone. (B) Ratio of deptor to actin. Mean ± SE of 4 independent experiments is shown. *p < 0.001 vs control; **p < TGFβ alone. (C) Ratio of 4EBP-1 to 4EBP-1. Mean ± SE of 4 independent experiments is shown. *p < 0.001 vs control; **p < TGFβ alone. (D) Ratio of phospho-S6 kinase to S6 kinase. Mean ± SE of 4 independent experiments is shown. *p < 0.001 vs control; **p < TGFβ alone. (E) Ratio of phospho-rps6 to rps6. Mean ± SE of 4 independent experiments is shown. *p < 0.001 vs control; **p < 0.01 vs TGFβ alone. (E) Ratio of phospho-rps6 to rps6. Mean ± SE of 4 independent experiments is shown. *p < 0.001 vs control; **p vs TGFβ alone. (F) Ratio of phospho-SGK1 to SGK1. Mean ± SE of 4 independent experiments is shown. *p < 0.001 vs control; **p vs TGFβ alone. (G) Ratio of phospho-NDRG1 to NDRG1. Mean ± SE of 4 independent experiments is shown. *p < 0.001 vs control; **p < TGFβ alone. (TIF) [file pone.0207285.s010.tif]

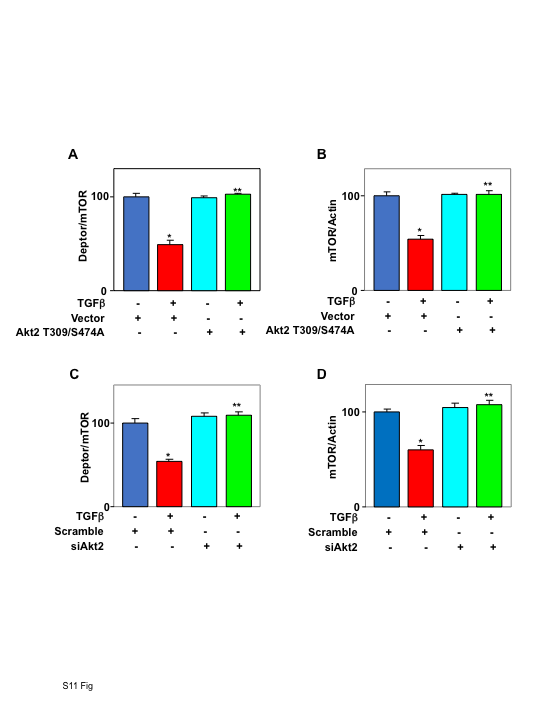

Supplement: S11 Fig — (A and C) Ratio of Deptor to mTOR. (B and D) Ratio of mTOR to actin. Mean ± SE of 3 independent experiments is shown. *p < 0.001 vs control; **p < 0.001 vs TGFβ. (TIF) [file pone.0207285.s011.tif]

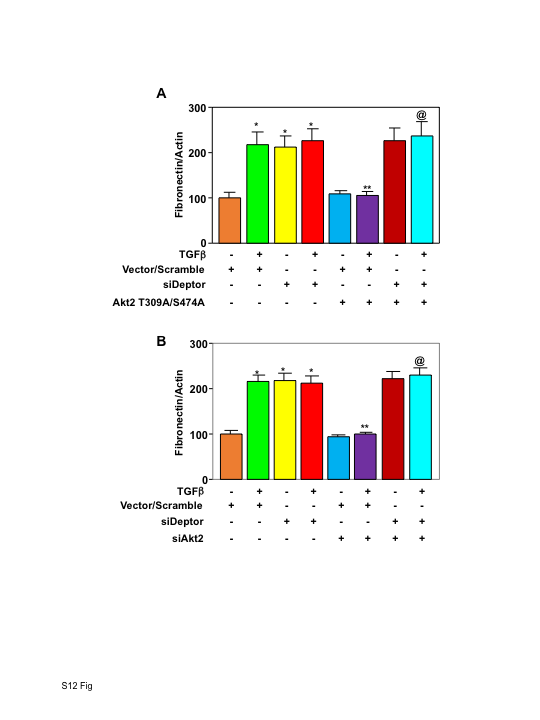

Supplement: S12 Fig — (A and B) Quantification of fibronectin expression shown in Fig 9C and 9D respectively. Ratio of fibronectin to actin. Mean ± SE of 3 independent experiments is shown. In panel A, *p < 0.05 vs control; **p < 0.05 vs TGFβ alone; @p < 0.05 vs TGFβ plus Akt2 T309A/S474A. In panel B, *p < 0.001 vs control; **p < 0.001 vs TGFβ alone; @p < 0.001 vs TGFβ plus siAkt2. (TIF) [file pone.0207285.s012.tif]

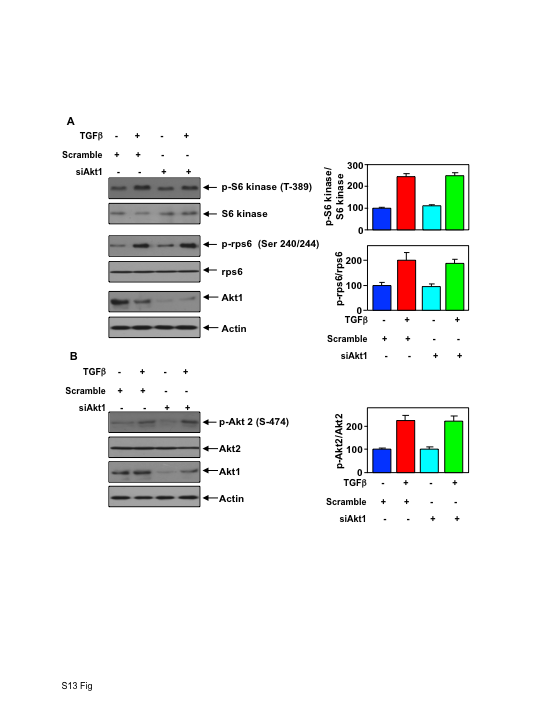

Supplement: S13 Fig — Podocytes were transfected with siRNA against Akt1. The transfected cells were incubated with 2 ng /ml TGFβ for 24 hours. The cell lysates were immunoblotted with phospho-S6 kinase (Thr-389) and phospho-rps6 (Ser-240/244) antibodies to detect mTORC1 activity (panel A). In panel B, the cell lysates were immunoblotted with phospho-Akt2 (Ser-474) antibody to determine mTORC2 activity [50]. For control, S6 kinase, rps6, Akt1, Akt2 and actin antibodies were used as indicated. The right part of each panel shows quantification of the data. Mean ± SE of 3 independent experiments is shown. There was no significant difference between TGFβ and TGFβ plus siAkt1. (TIF) [file pone.0207285.s013.tif]
