# Supplementary material for: Akt2 causes TGFβ-induced deptor downregulation facilitating mTOR to drive podocyte hypertrophy and matrix protein expression
Source: PLoS One. 2018 Nov 16;13(11):e0207285. doi: 10.1371/journal.pone.0207285 (PMC6239304; doi:10.1371/journal.pone.0207285)

Supporting Raw Data

Fig. 1A

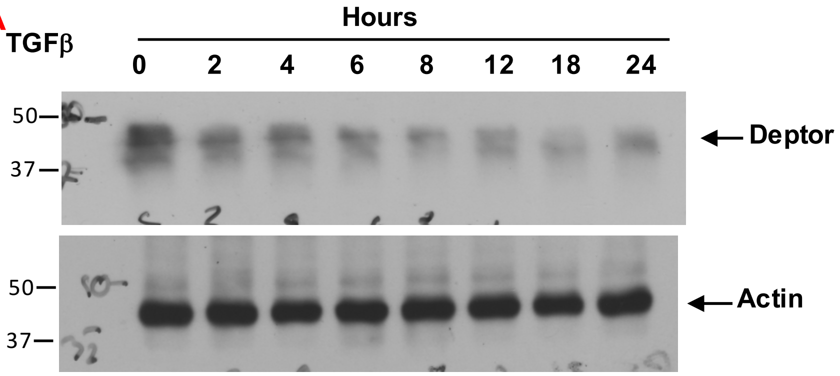

Fig. 1B

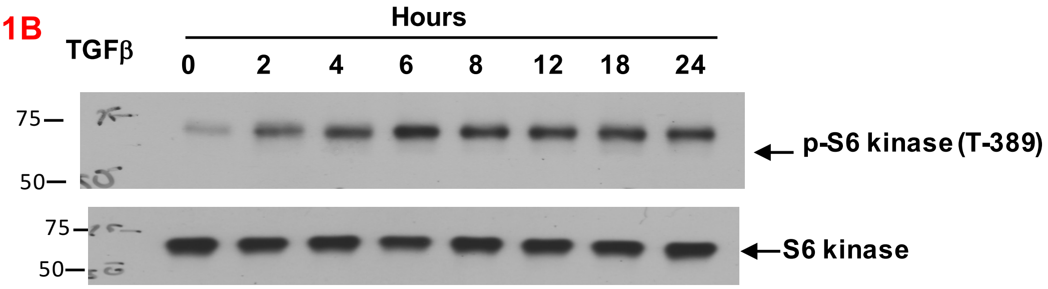

Fig. 1C

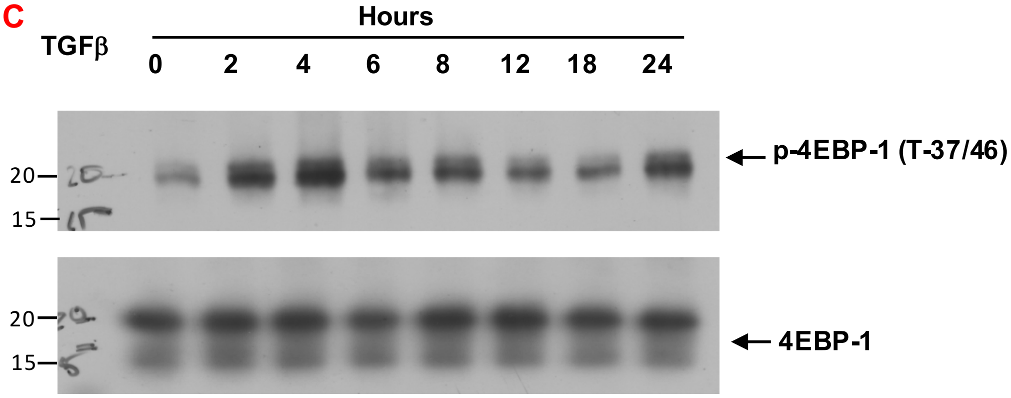

Fig. 1D

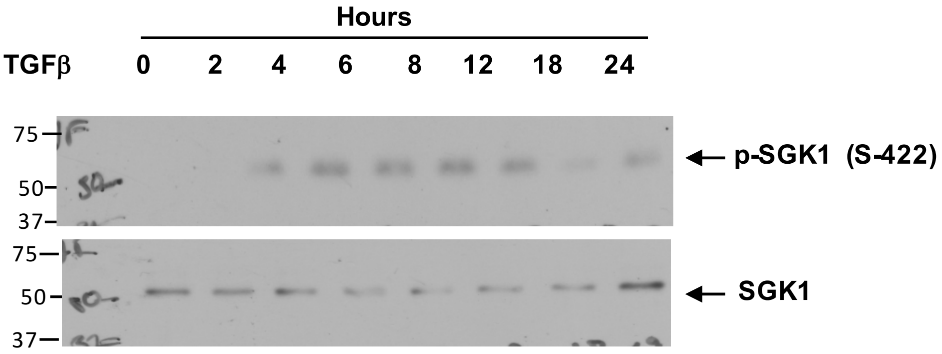

Fig. 1E

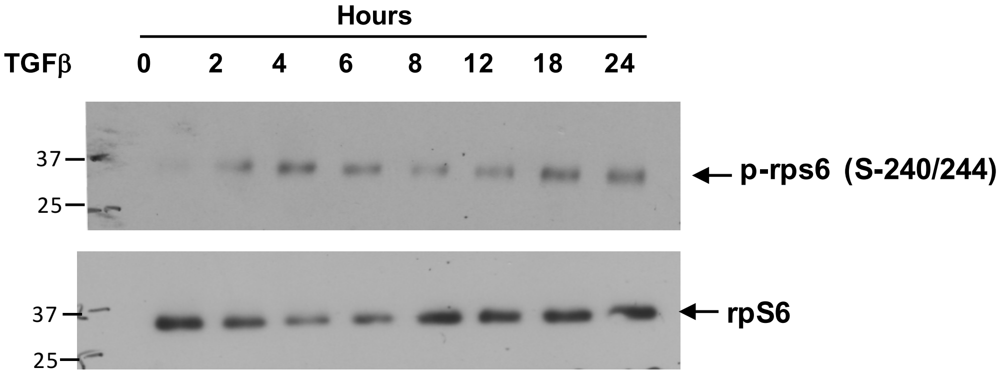

Fig. 1F

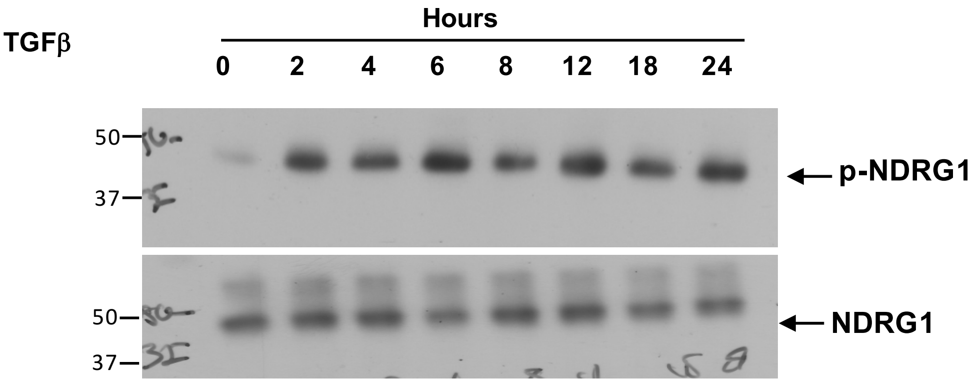

Fig. 2A

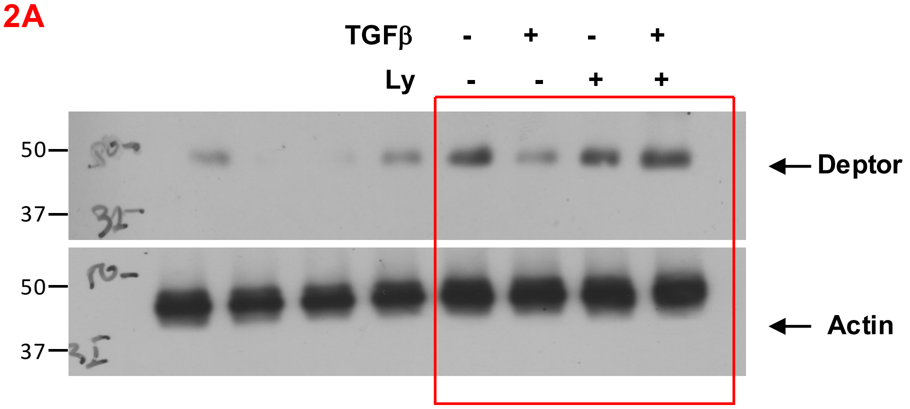

Fig. 2B

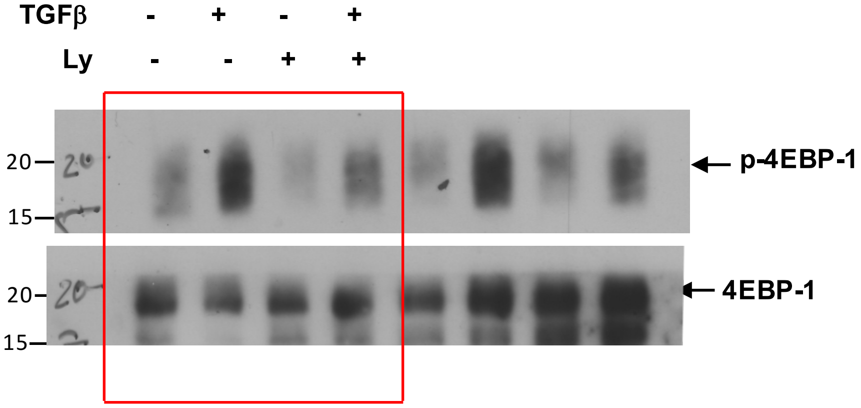

Fig. 2C

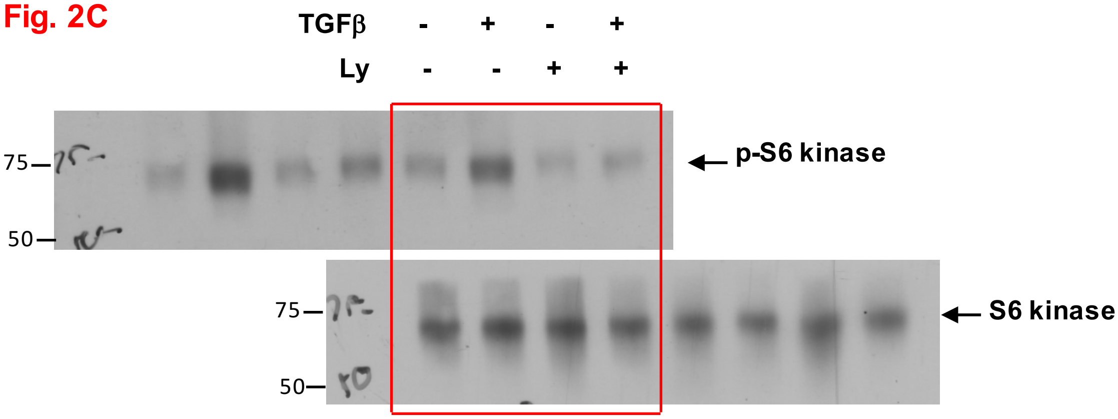

Fig. 2D

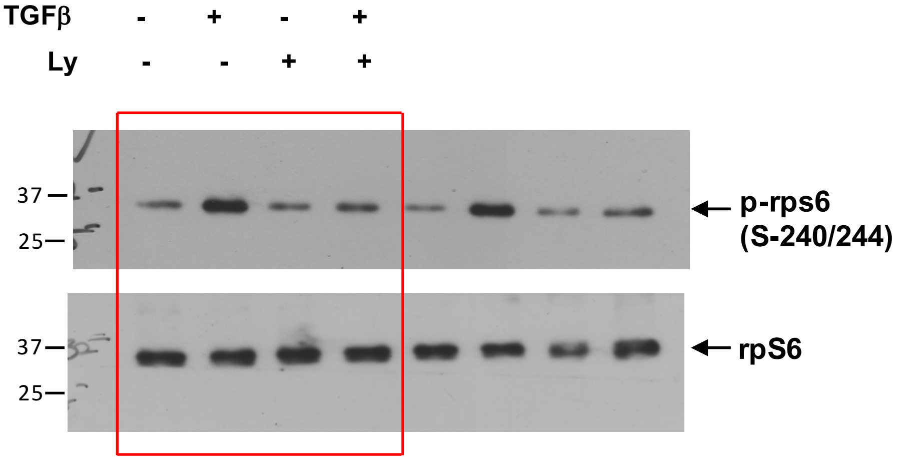

Fig. 2E

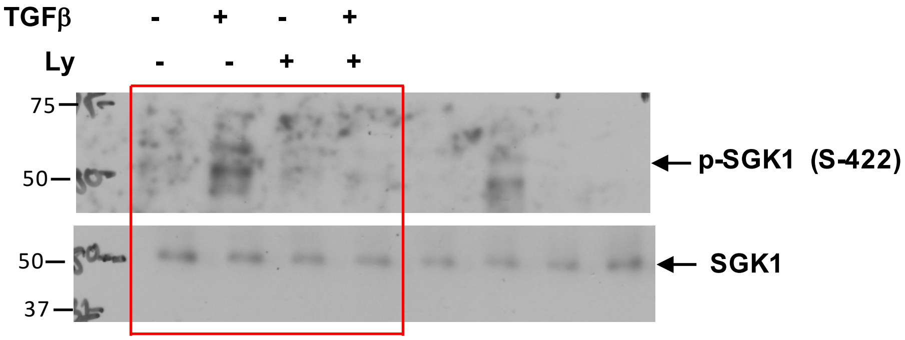

Fig. 2F

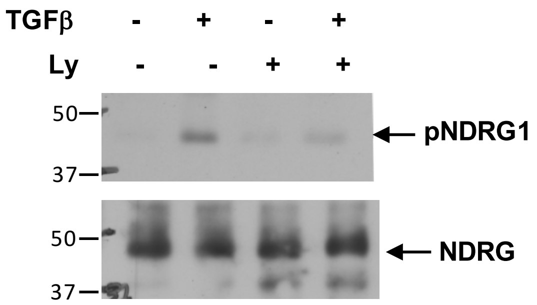

Fig. 2G

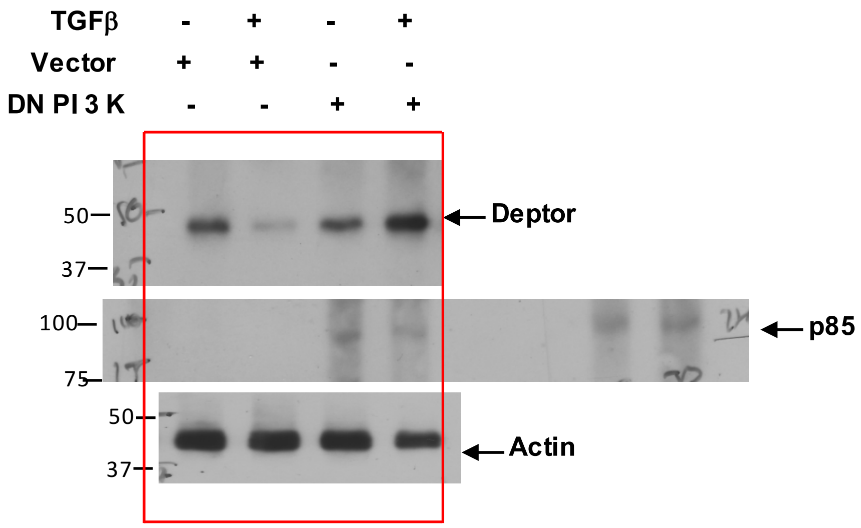

Fig. 2H

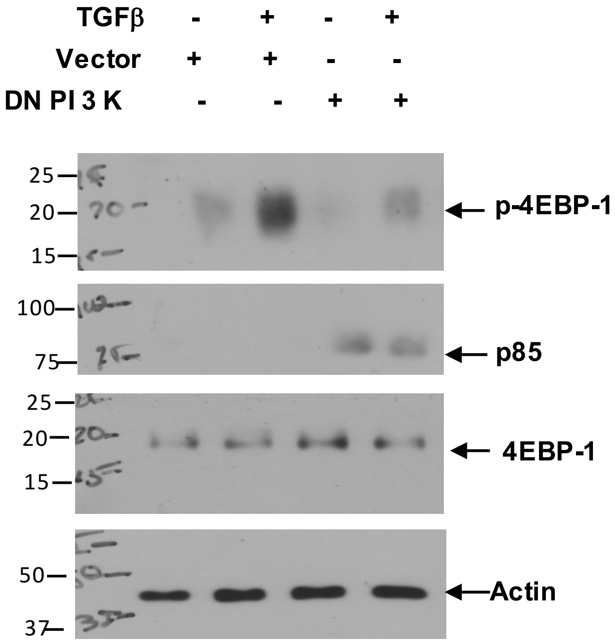

Fig. 2I

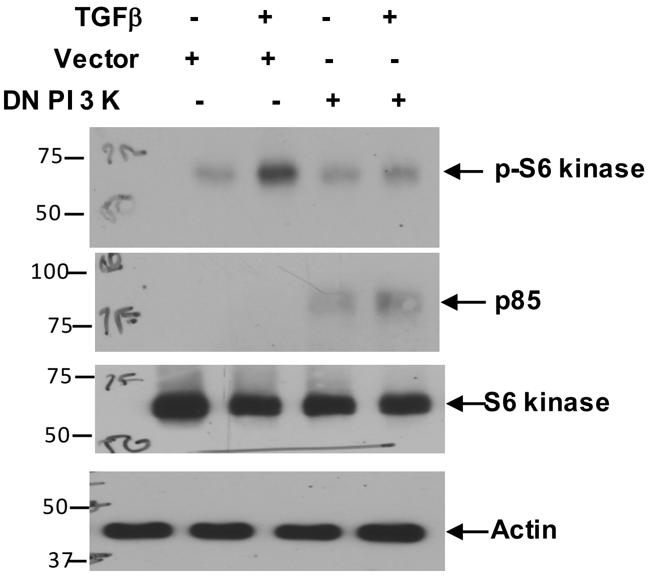

Fig. 2J

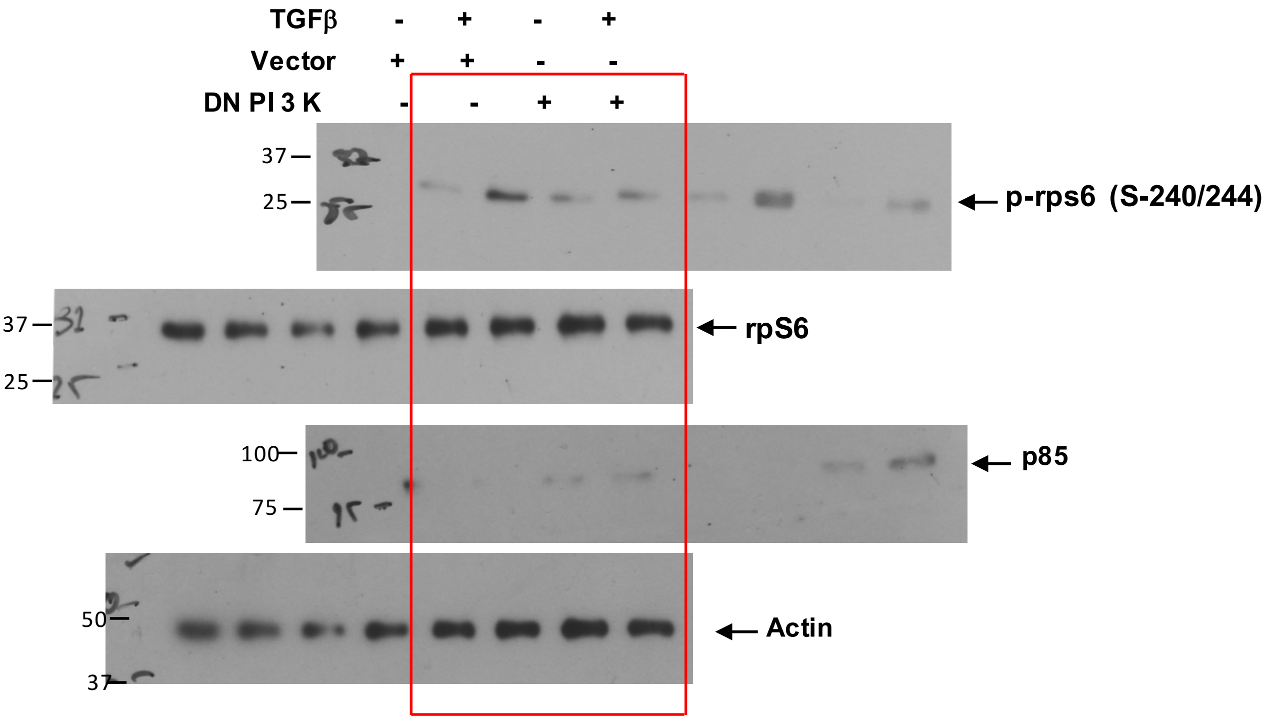

Fig. 2K

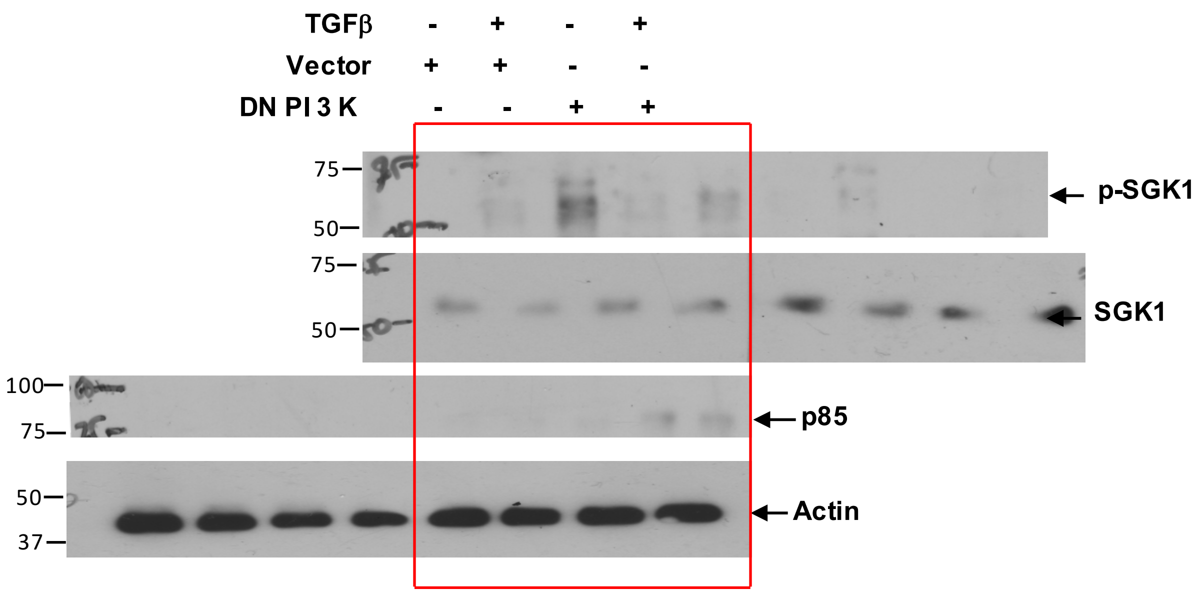
TGFβ - + - +
Vector + + - -
DN PI 3 K - - + +
75
50
p-SGK1
75
50
SGK1
100
75
p85
50
37
Actin

Fig. 2L

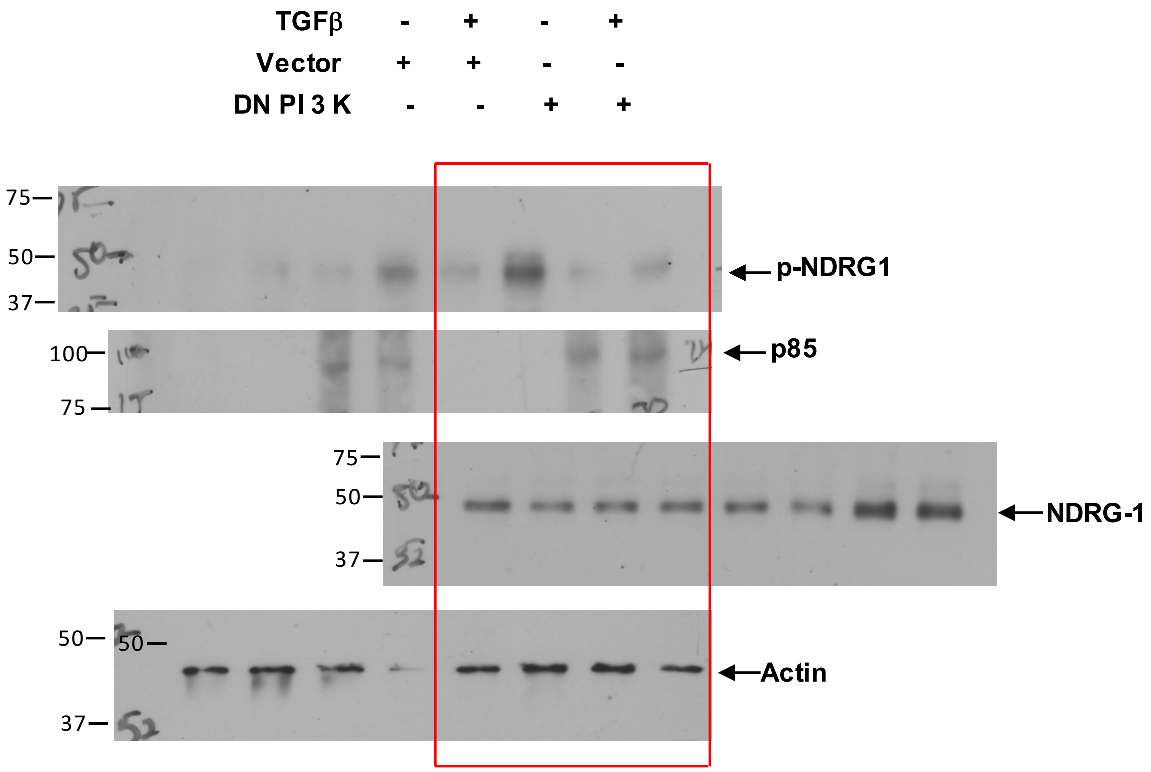
TGFβ - + - +
Vector + + - -
DN PI 3 K - - + +
75
50
37
p-NDRG1
100
75
p85
75
50
37
NDRG-1
50
50
37
Actin

Fig. 3A

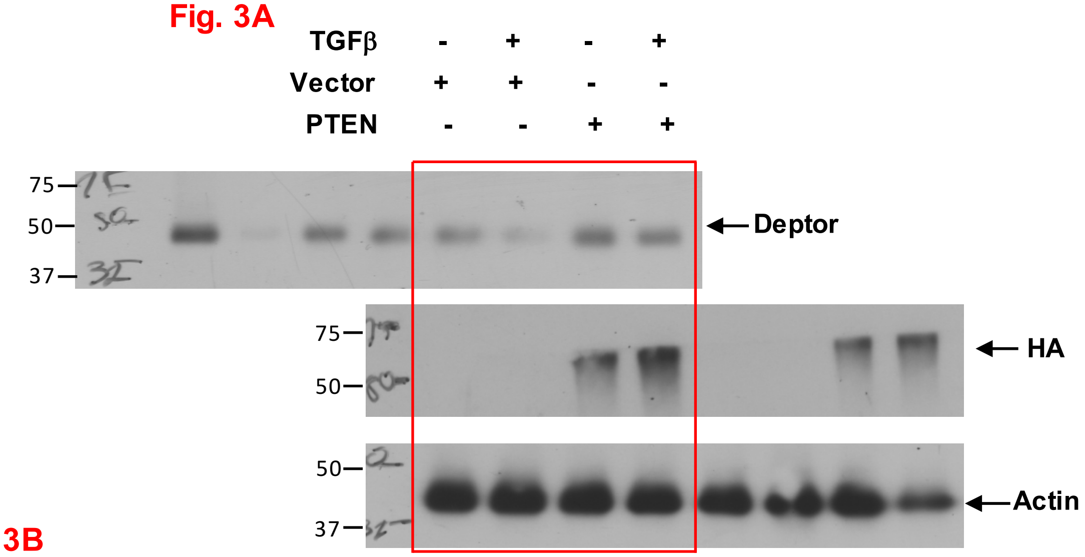

Fig. 3B

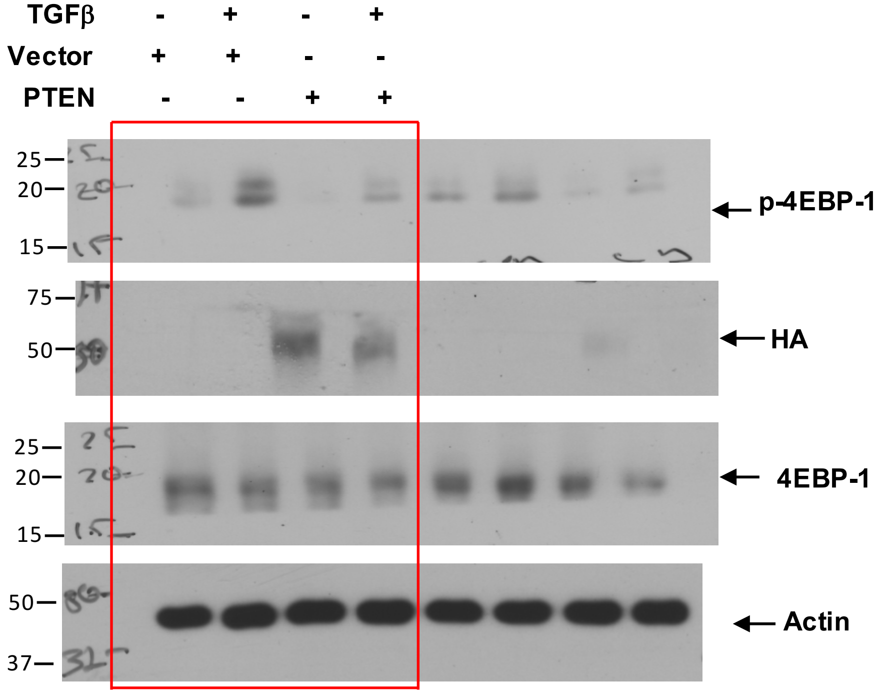

Fig. 3C

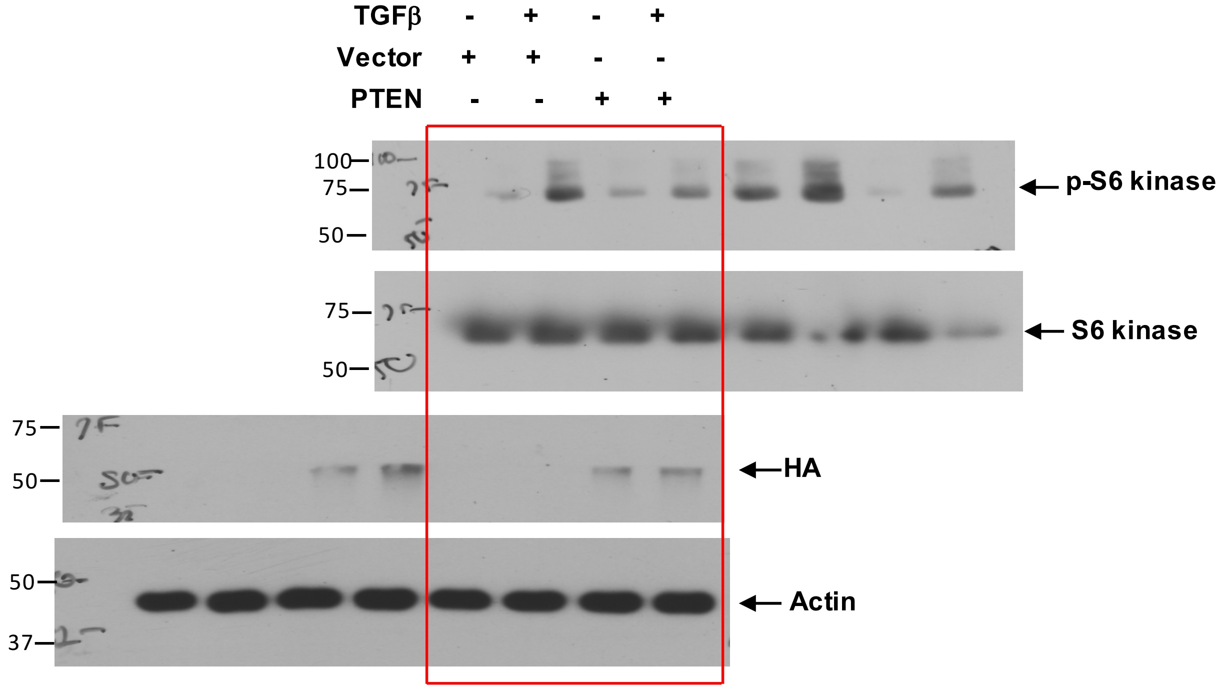

Fig. 3D

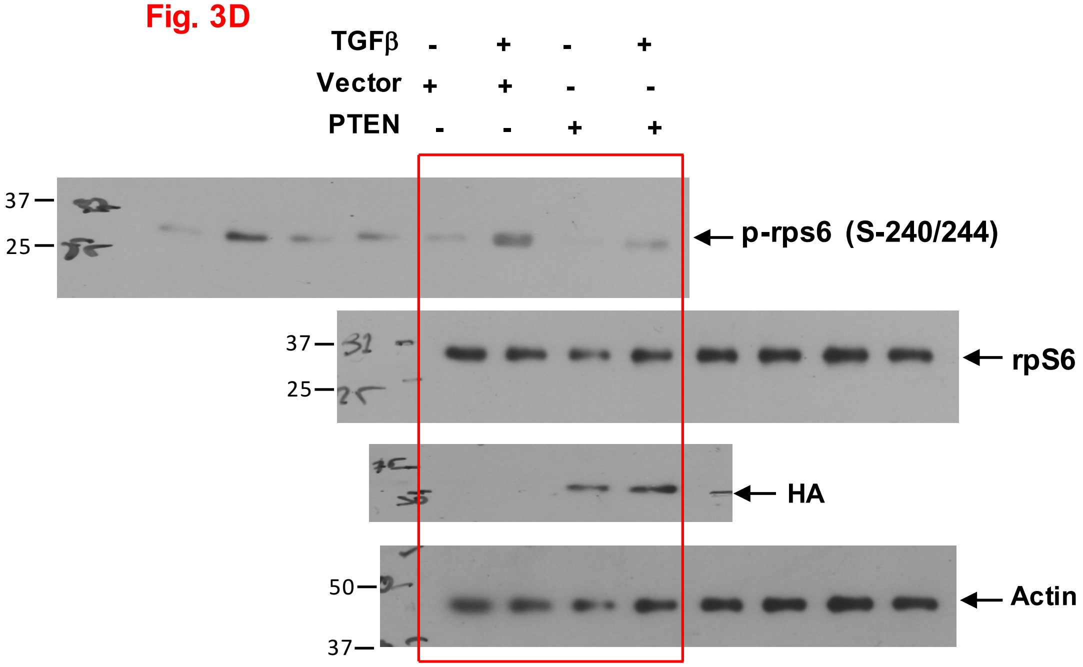

Fig. 3E

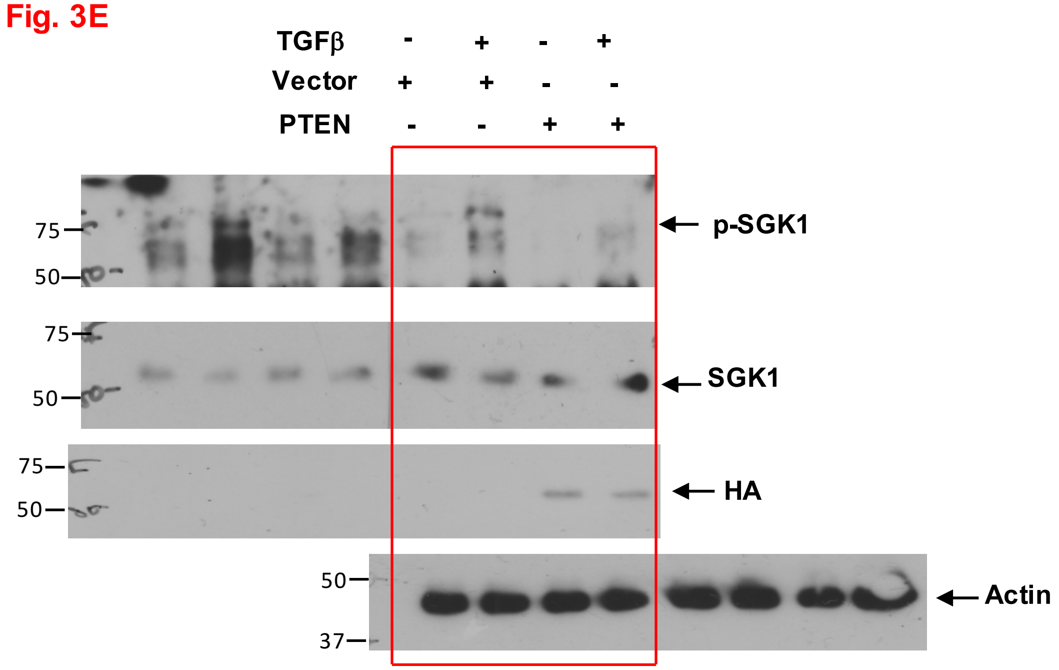

Fig. 3F

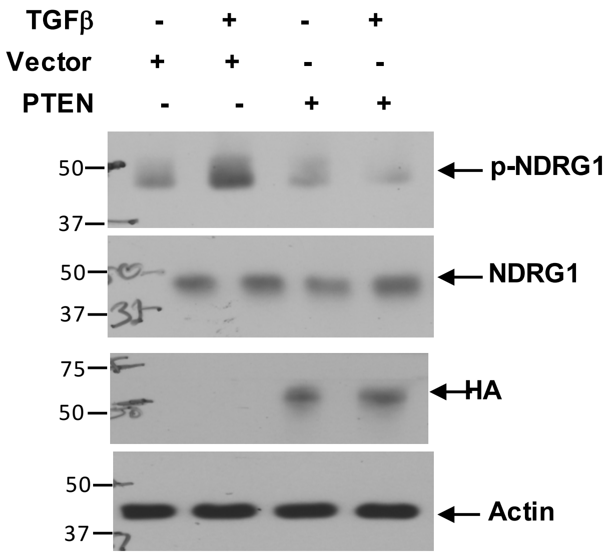

Fig. 4A

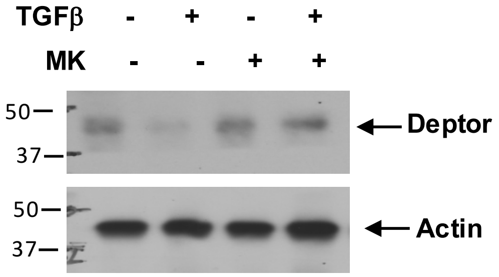

Fig. 4B

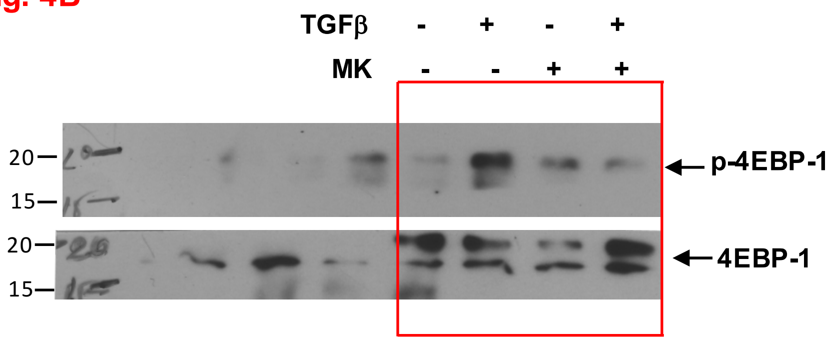

Fig. 4C

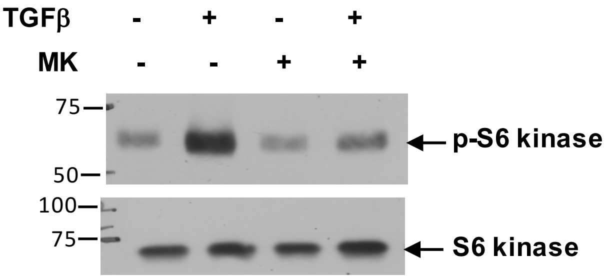

Fig. 4D

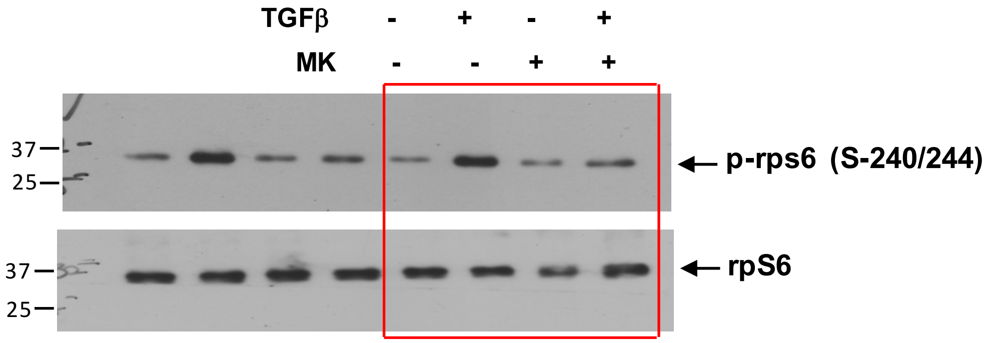

Fig. 4E

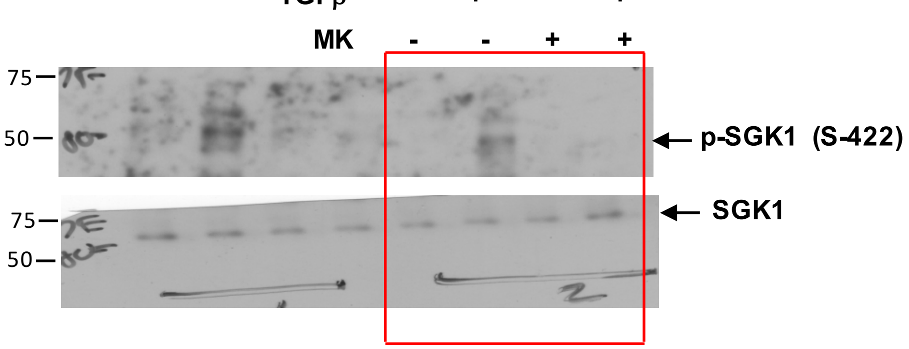

Fig. 4F

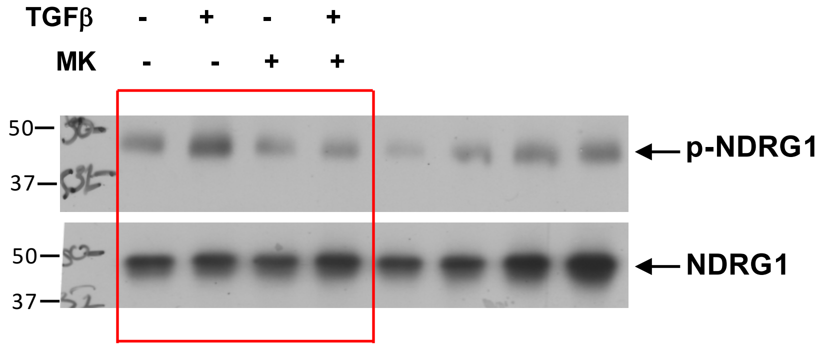

Fig. 5A

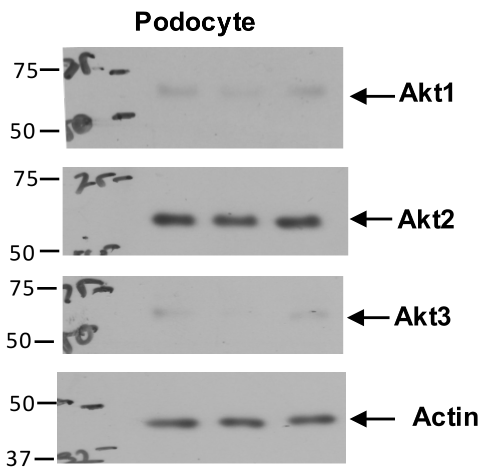

Fig. 5B

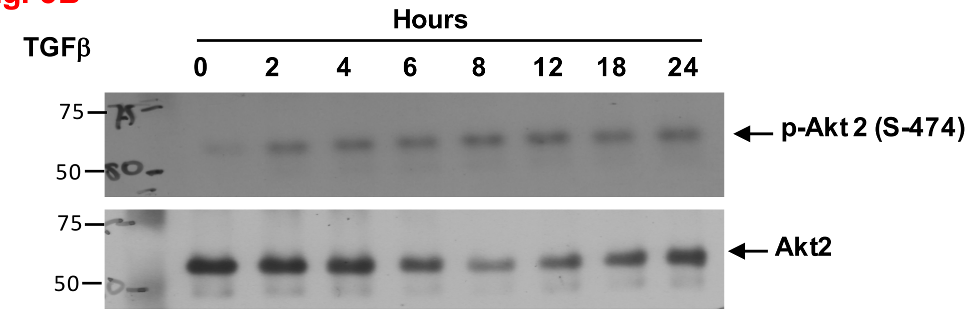

Fig. 5C

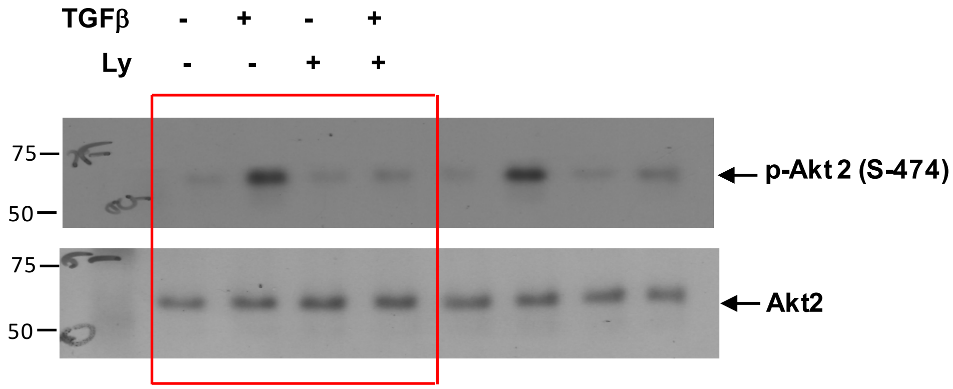

Fig. 5D

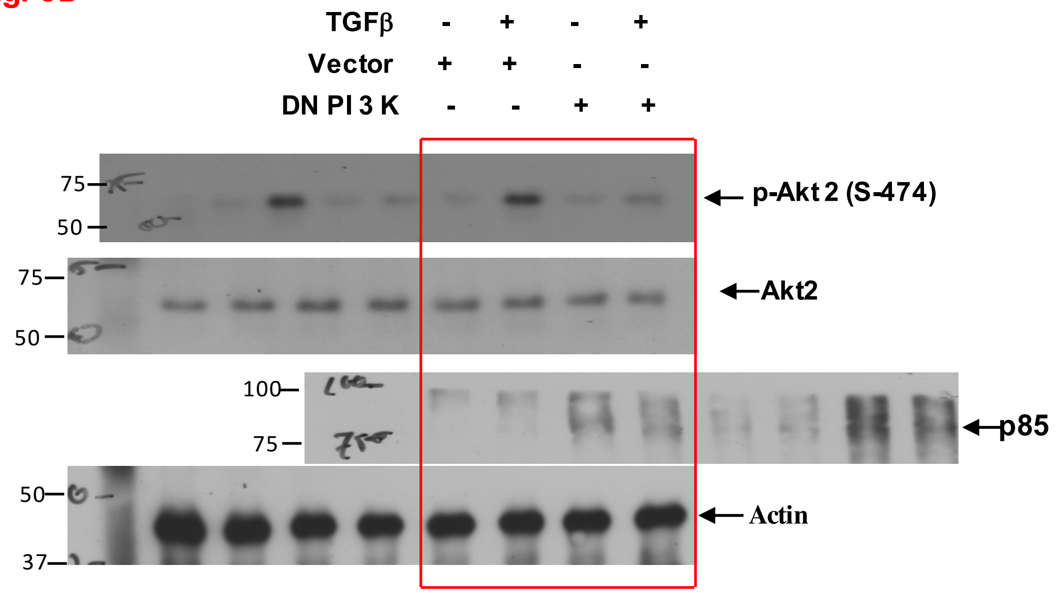

TGFβ - + - +
Vector + + - -
DN PI 3 K - - + +
75
50
p-Akt 2 (S-474)
75
50
Akt2
100
75
p85
50
37
Actin

Fig. 5E

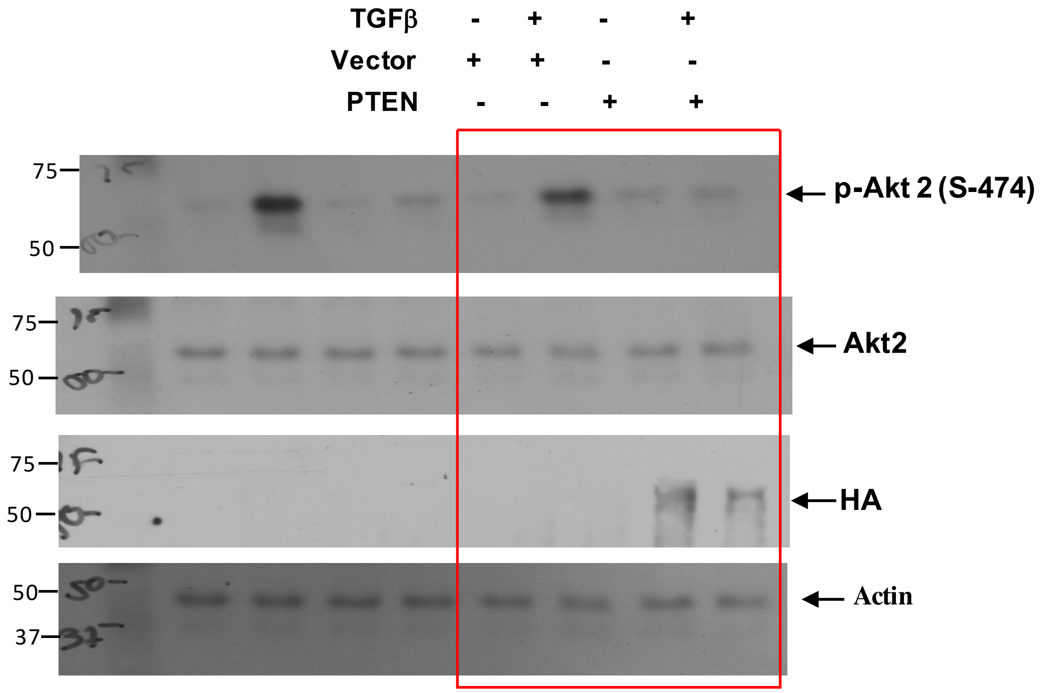

TGFβ - + - +
Vector + + - -
PTEN - - + +
75
50
p-Akt 2 (S-474)
75
50
Akt2
75
50
HA
50
37
Actin

Fig. 6A

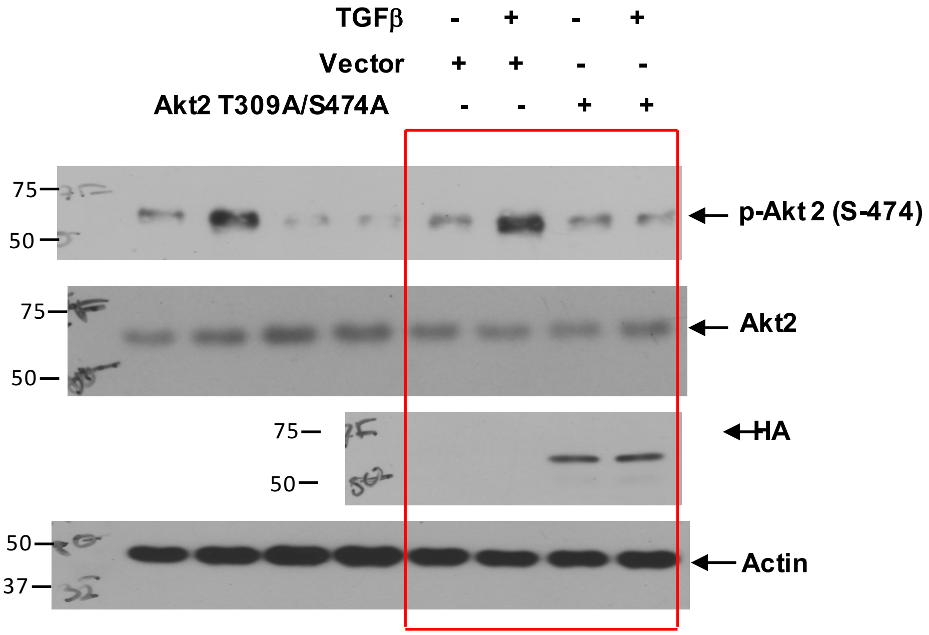

Fig. 6B

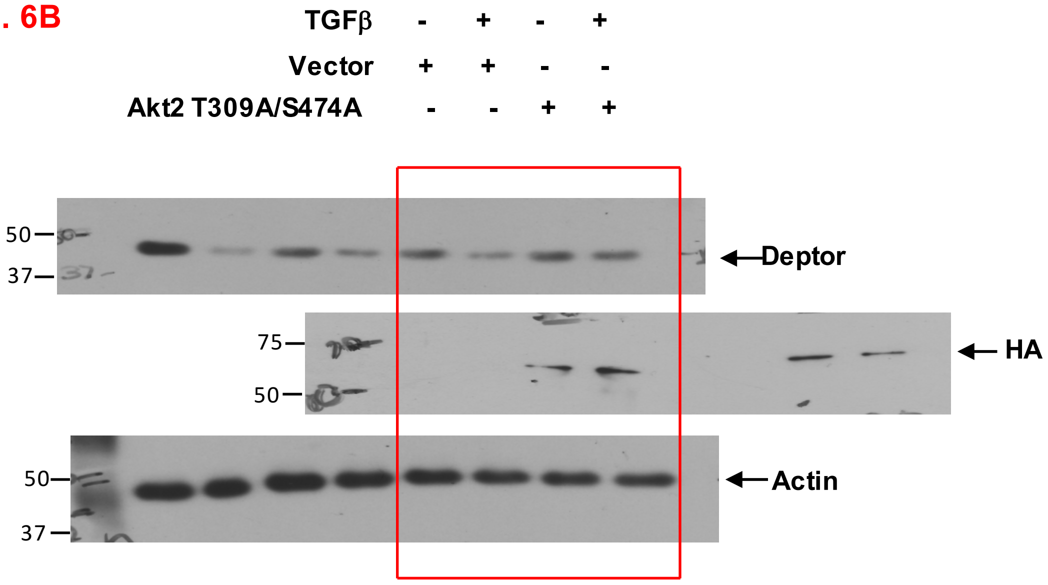

Fig. 6C

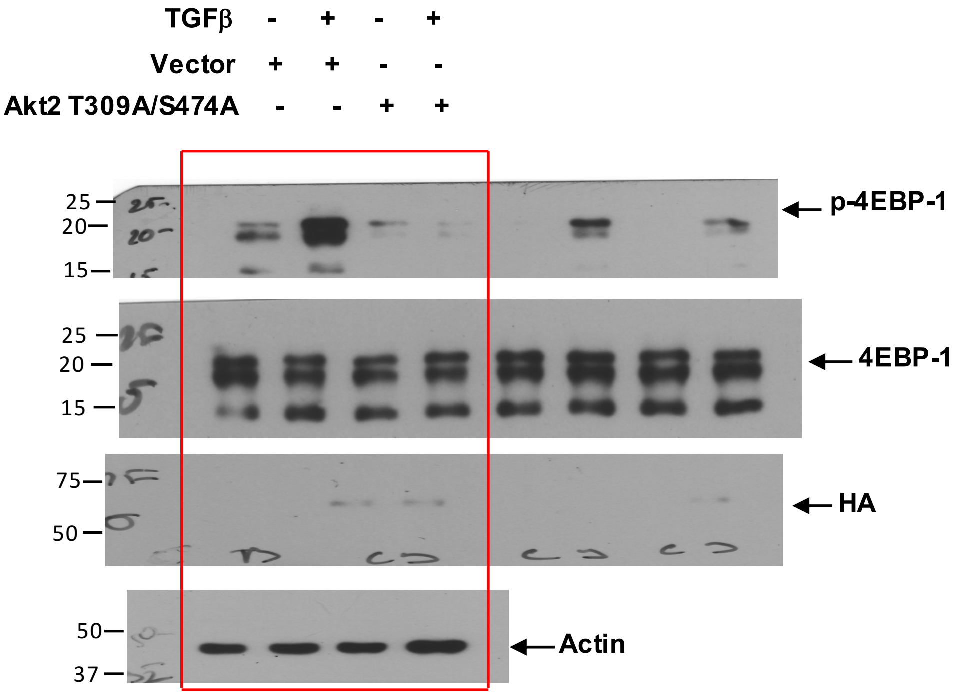

Fig. 6D

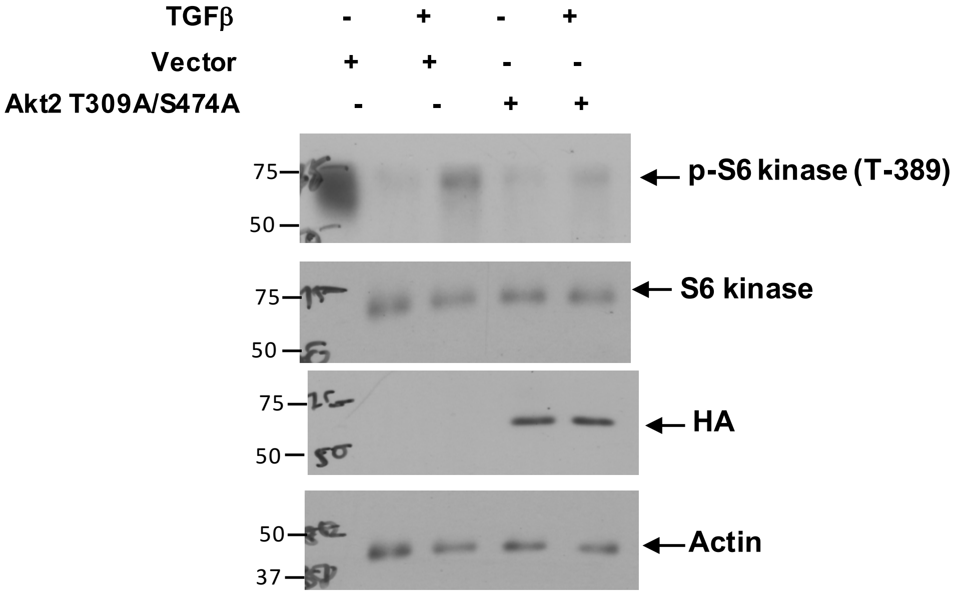

Fig. 6E

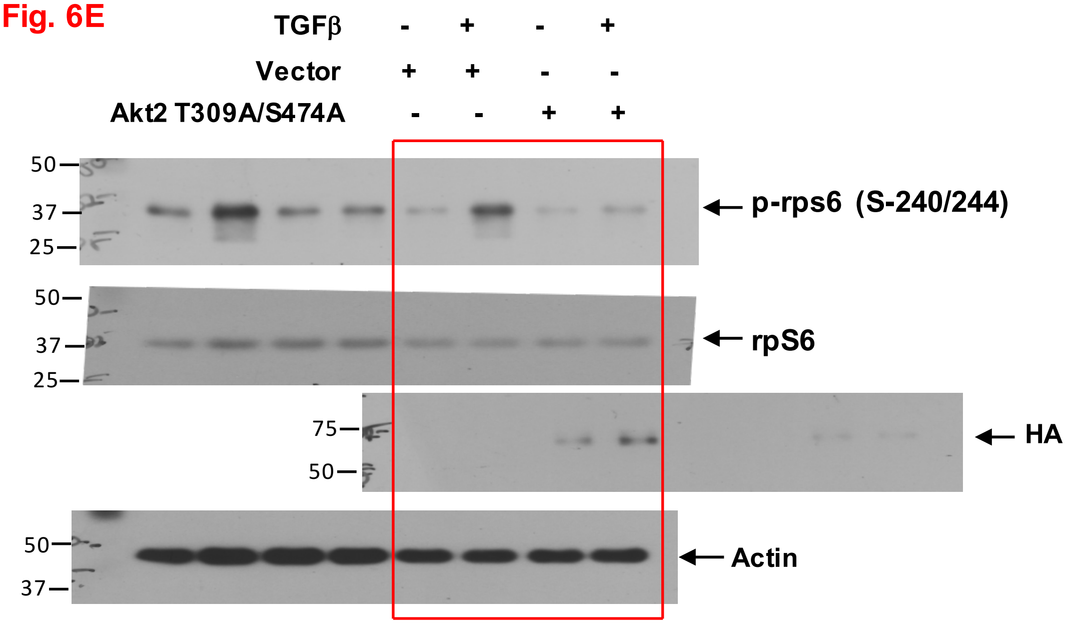

Fig. 6F

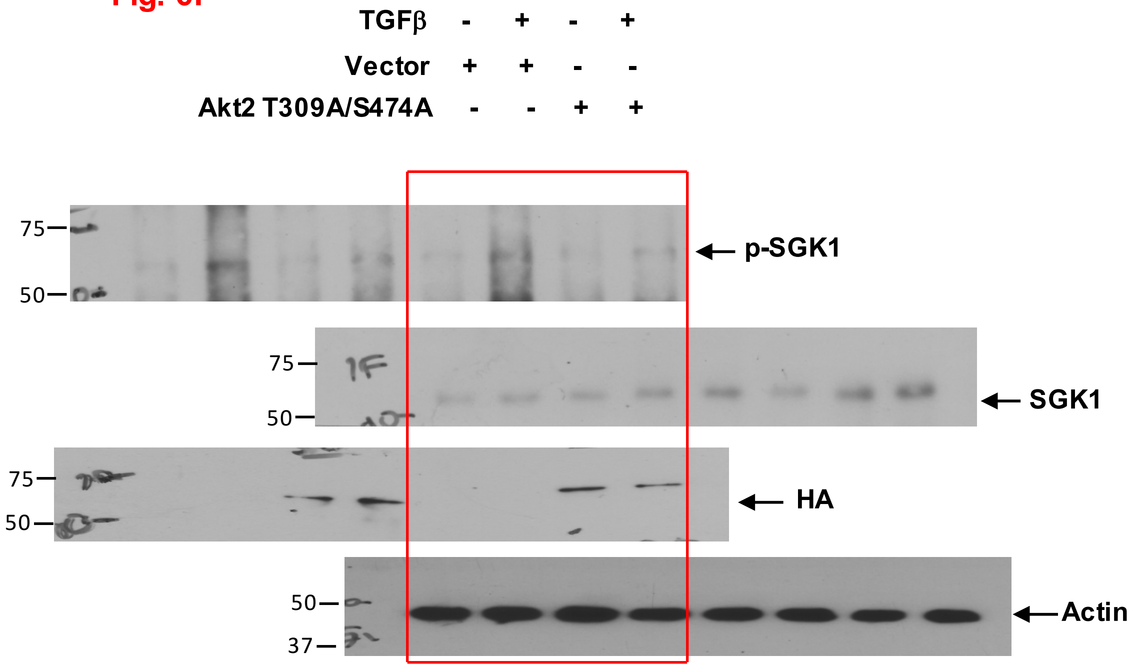

Fig. 6G

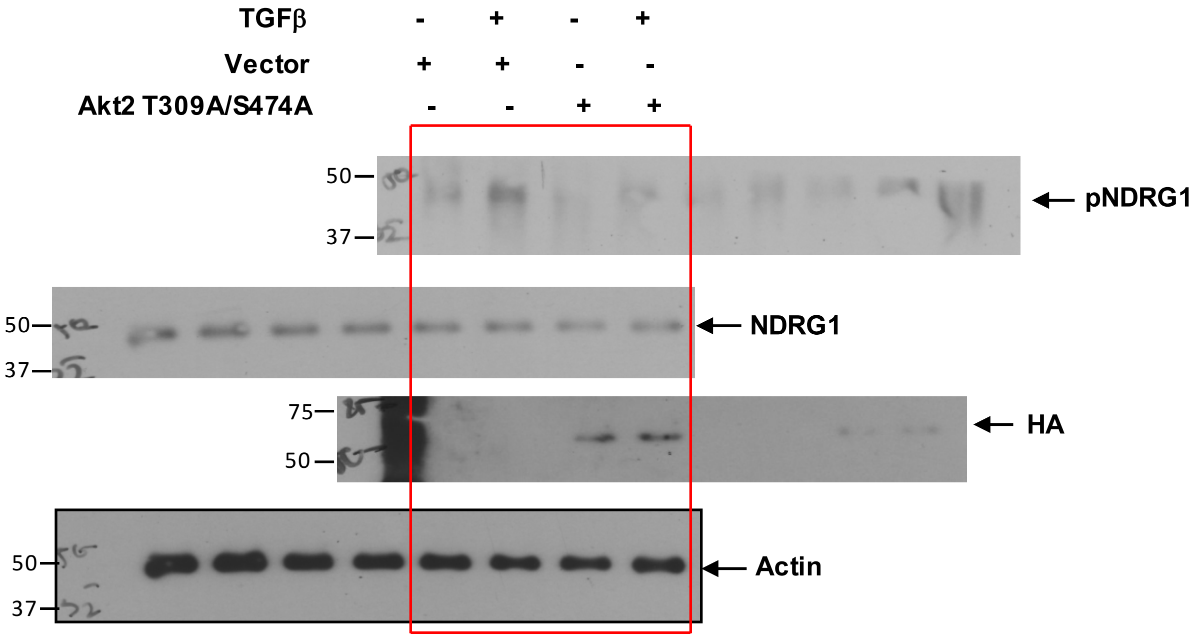
TGFβ - + - +
Vector + + - -
Akt2 T309A/S474A - - + +
50
37
pNDRG1
50
37
NDRG1
75
50
HA
50
37
Actin

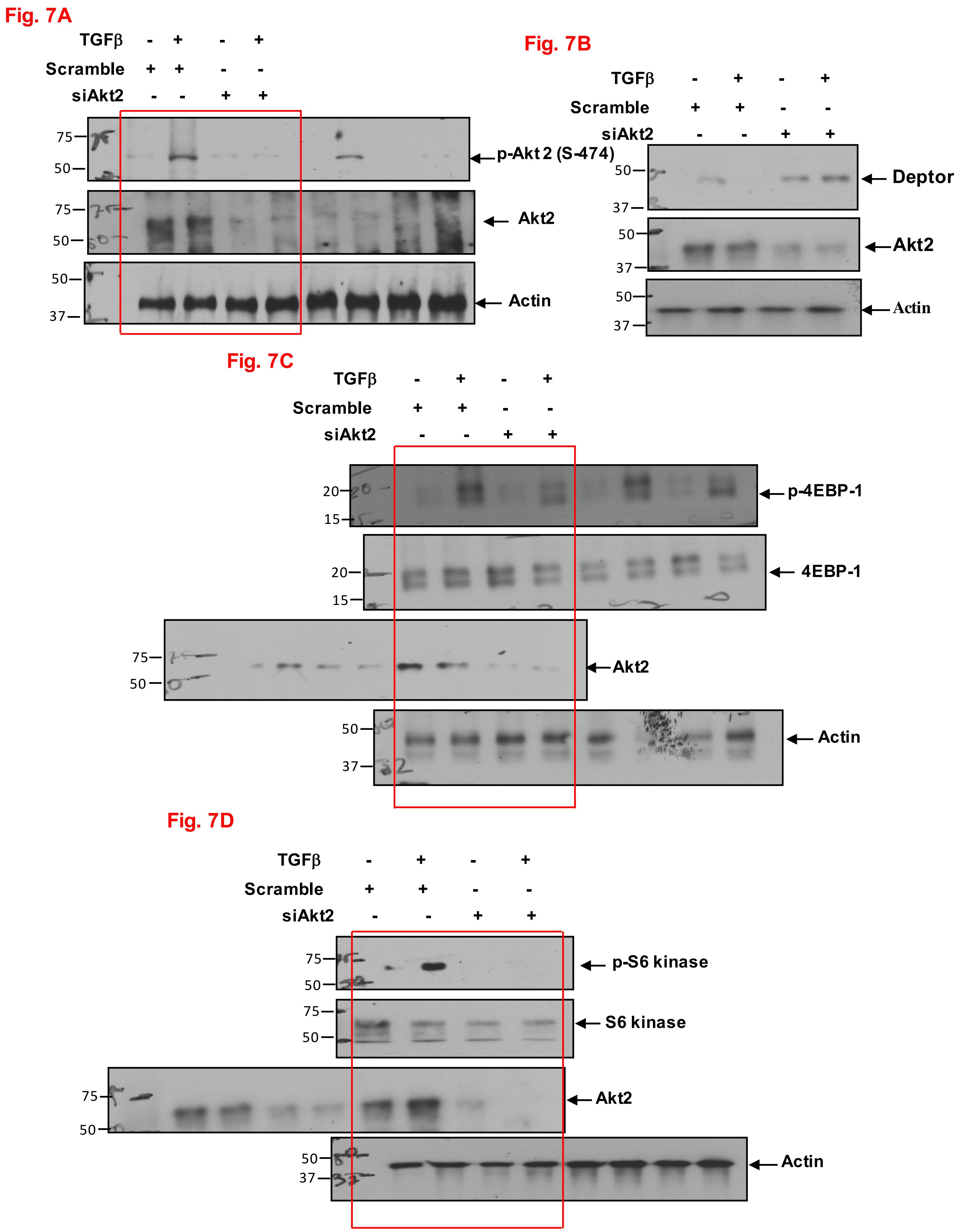
Fig. 7A
TGFβ - + - +
Scramble + + - -
siAkt2 - - + +
75
50
p-Akt 2 (S-474)
75
50
Akt2
50
37
Actin
Fig. 7B
TGFβ - + - +
Scramble + + - -
siAkt2 - - + +
50
37
Deptor
50
37
Akt2
50
37
Actin
Fig. 7C
TGFβ - + - +
Scramble + + - -
siAkt2 - - + +
20
15
p-4EBP-1
20
15
4EBP-1
75
50
Akt2
50
37
Actin
Fig. 7D
TGFβ - + - +
Scramble + + - -
siAkt2 - - + +
75
50
p-S6 kinase
75
50
S6 kinase
75
50
Akt2
50
37
Actin

Fig. 7E

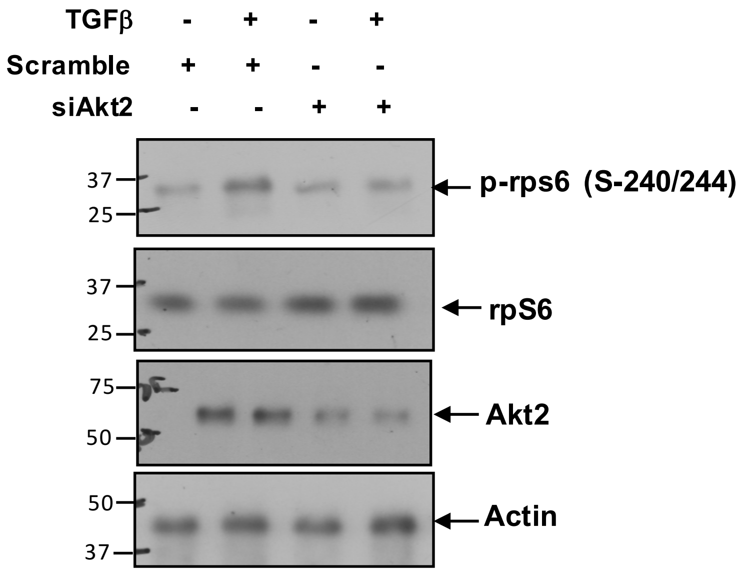

Fig. 7F

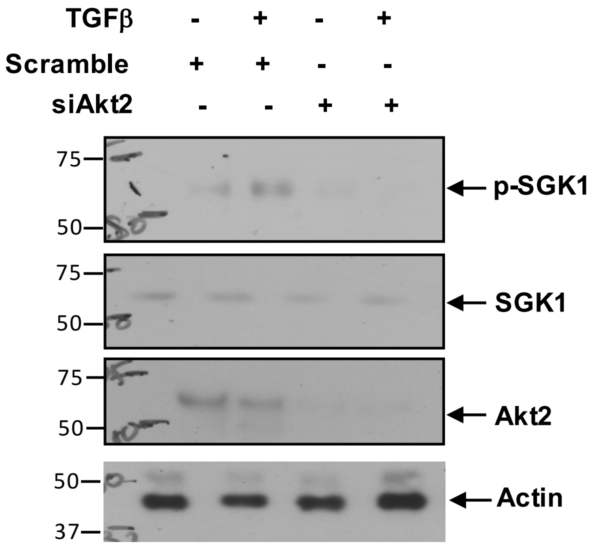

Fig. 7G

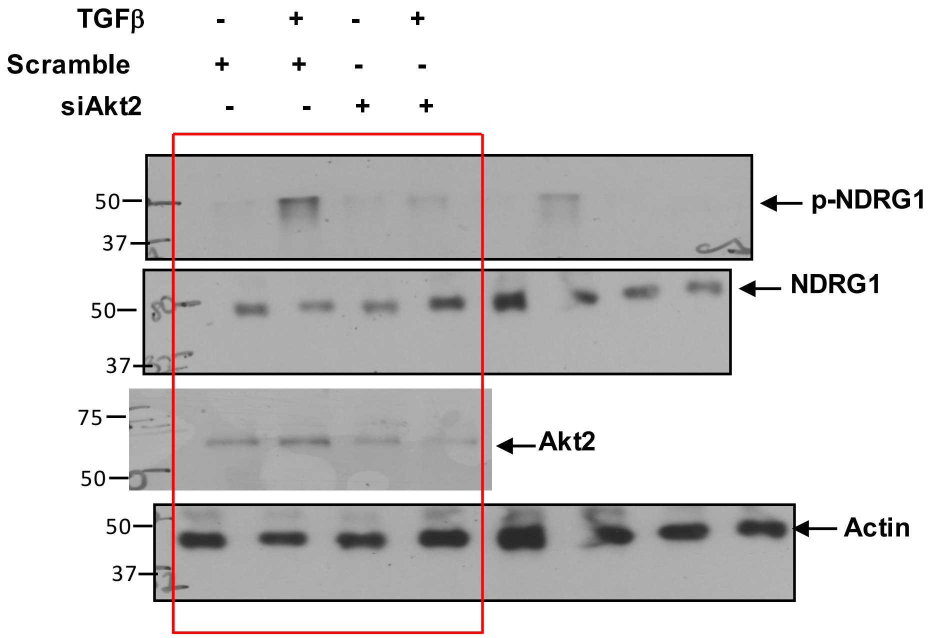

Fig. 8A

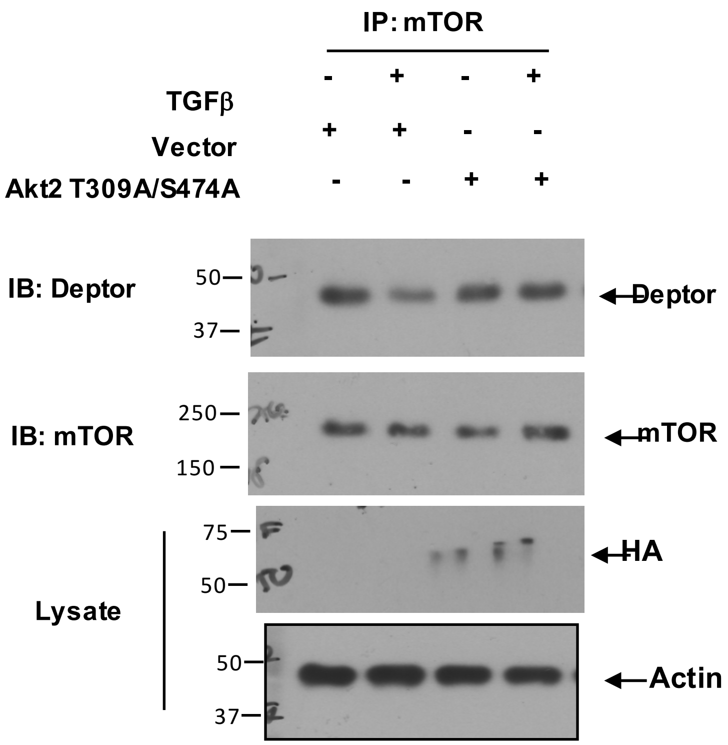
IP: mTOR
TGFβ - + - +
Vector + + - -
Akt2 T309A/S474A - - + +
IB: Deptor
50
37
Deptor
IB: mTOR
250
150
mTOR
Lysate
75
50
HA
50
37
Actin

Fig. 8B

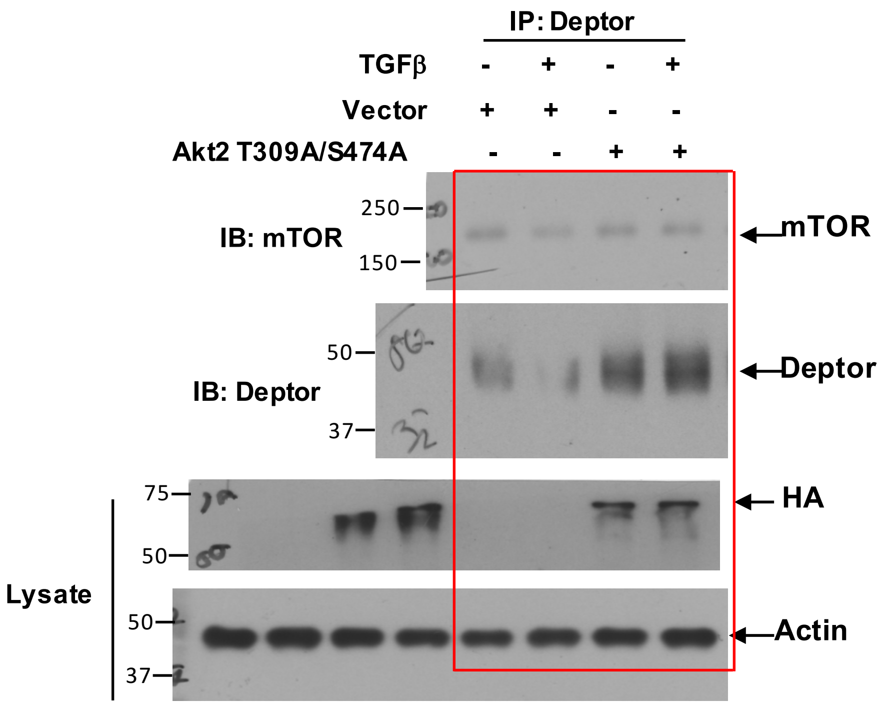
IP: Deptor
TGFβ - + - +
Vector + + - -
Akt2 T309A/S474A - - + +
IB: mTOR
250
150
mTOR
IB: Deptor
50
37
Deptor
Lysate
75
50
HA
50
37
Actin

Fig. 8C

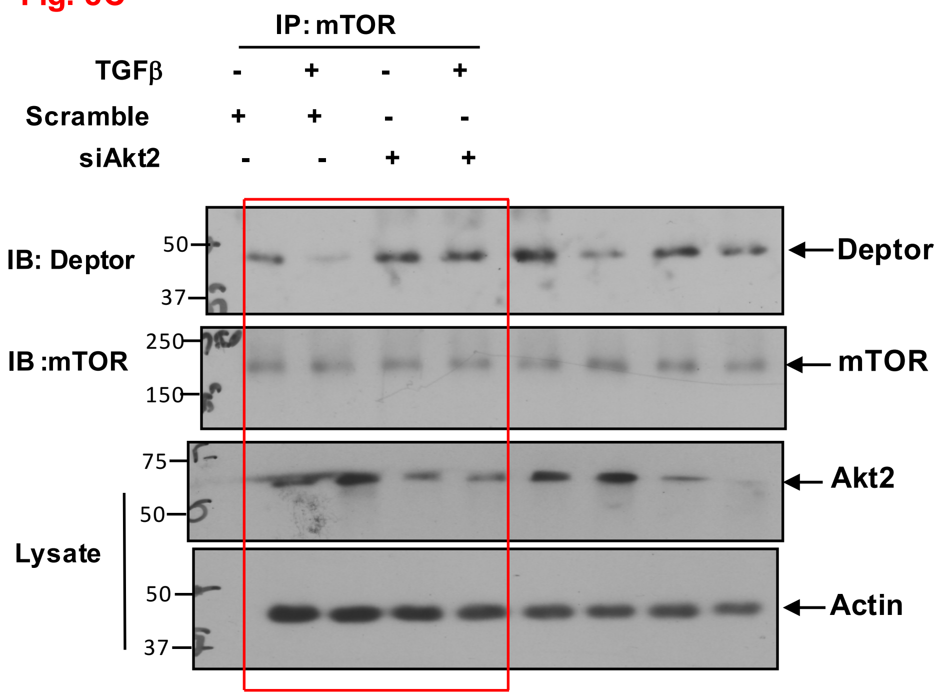

IP: mTOR
TGFβ - + - +
Scramble + + - -
siAkt2 - - + +
IB: Deptor
50
37
Deptor
IB :mTOR
250
150
mTOR
75
50
Akt2
Lysate
50
37
Actin

Fig. 8D

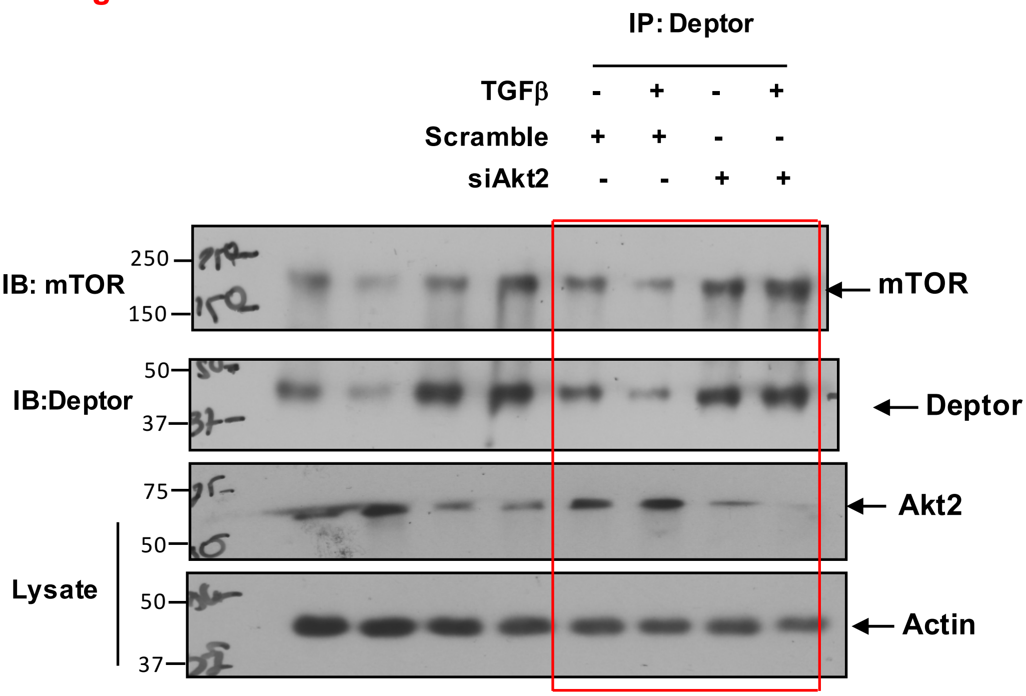

IP: Deptor
TGFβ - + - +
Scramble + + - -
siAkt2 - - + +
IB: mTOR
250
150
mTOR
IB:Deptor
50
37
Deptor
75
50
Akt2
Lysate
50
37
Actin

Fig. 9A bottom panel

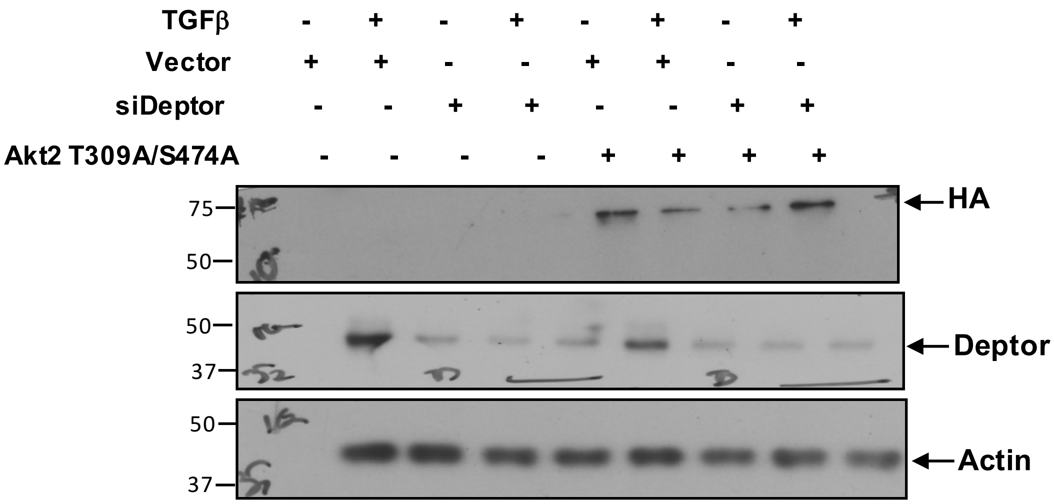

Fig. 9B bottom panel

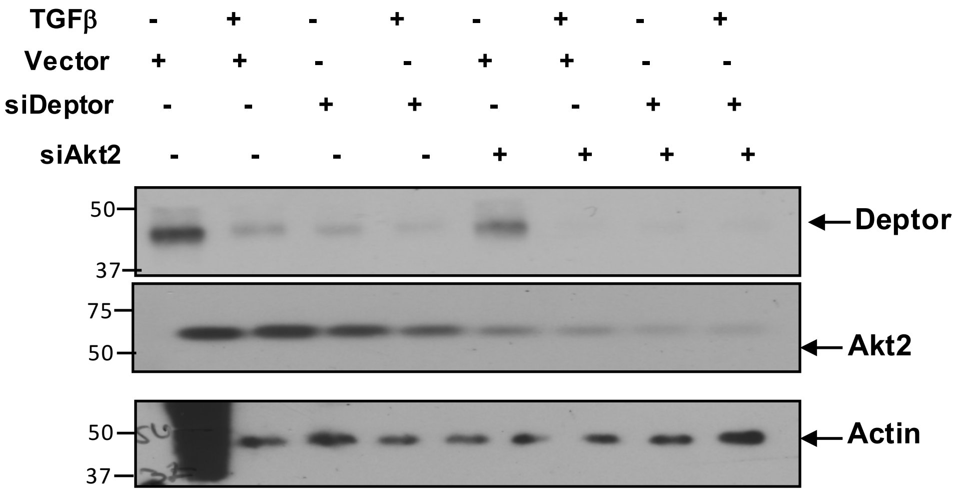

Fig. 9C

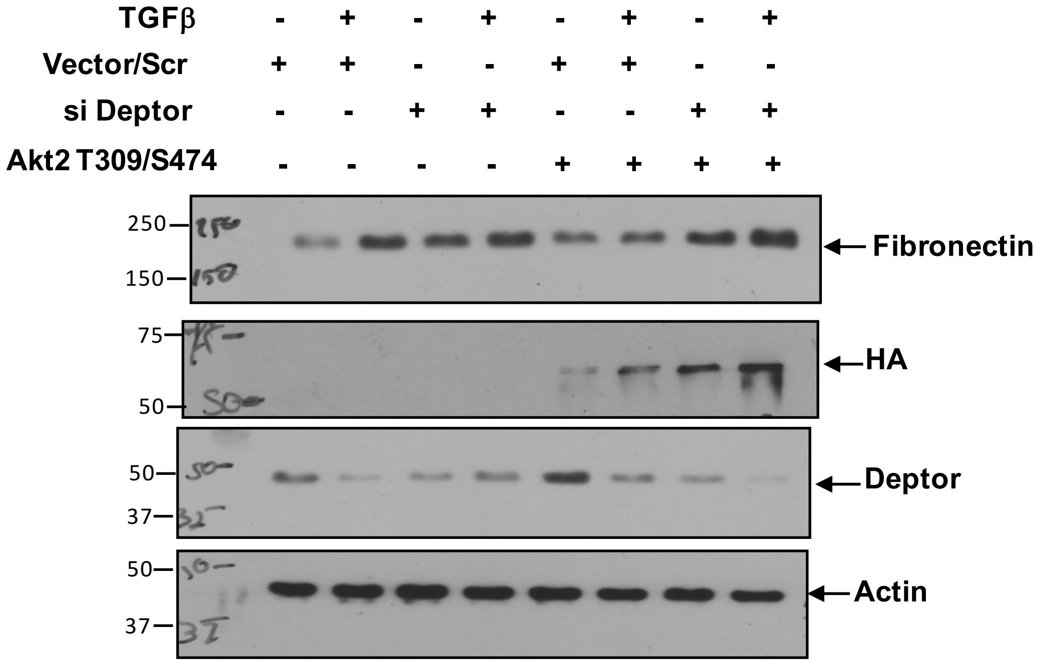
TGFβ - + - + - + - +
Vector/Scr + + - - + + - -
si Deptor - - + + - - + +
Akt2 T309/S474 - - - - + + + +
250
150
Fibronectin
75
50
HA
50
37
Deptor
50
37
Actin

Fig. 9D

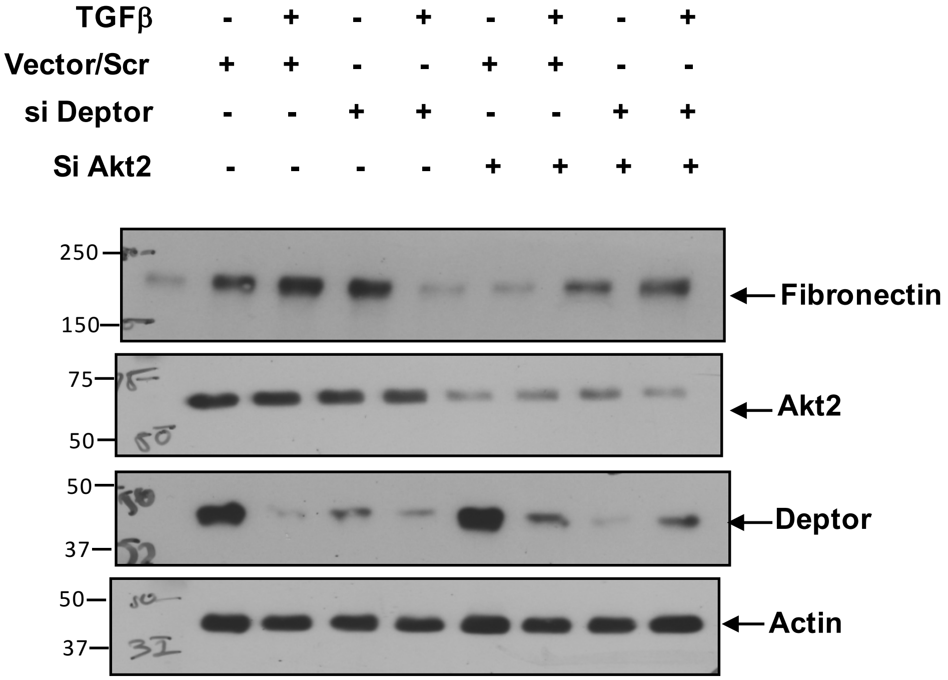
TGFβ - + - + - + - +
Vector/Scr + + - - + + - -
si Deptor - - + + - - + +
Si Akt2 - - - - + + + +
250
150
Fibronectin
75
50
Akt2
50
37
Deptor
50
37
Actin

Fig. 10A

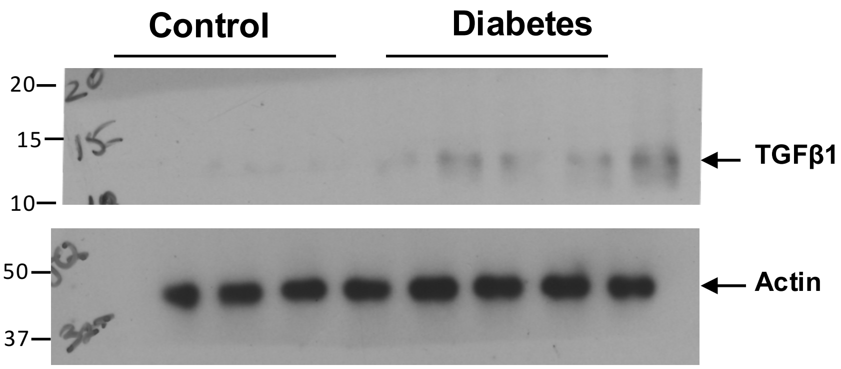

Fig. 10C

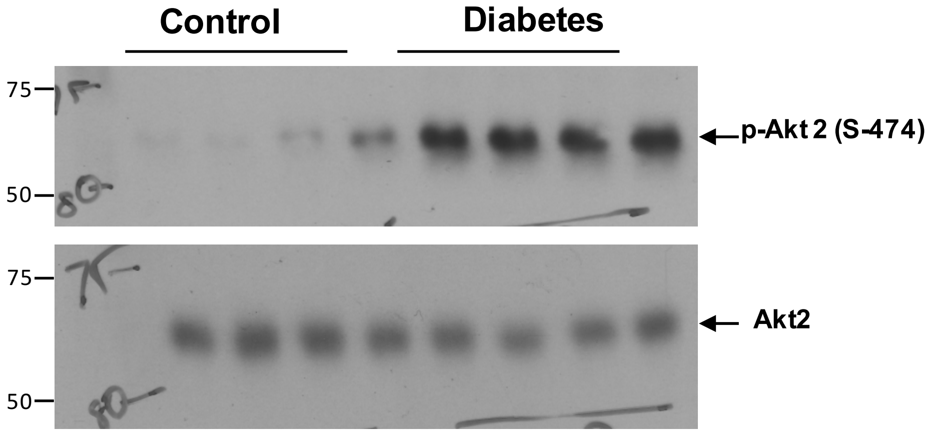

Fig. 10E

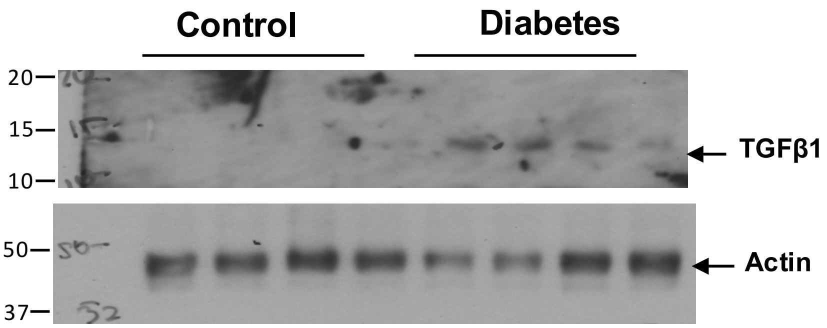

Fig. 10G

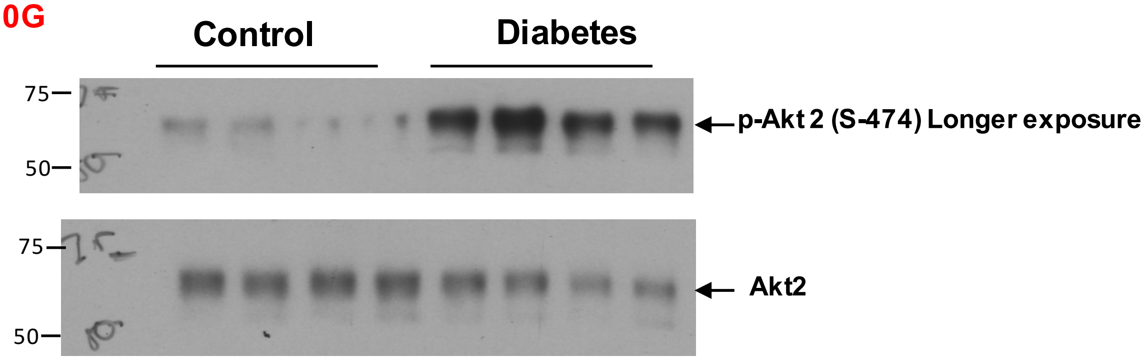

S1A Fig

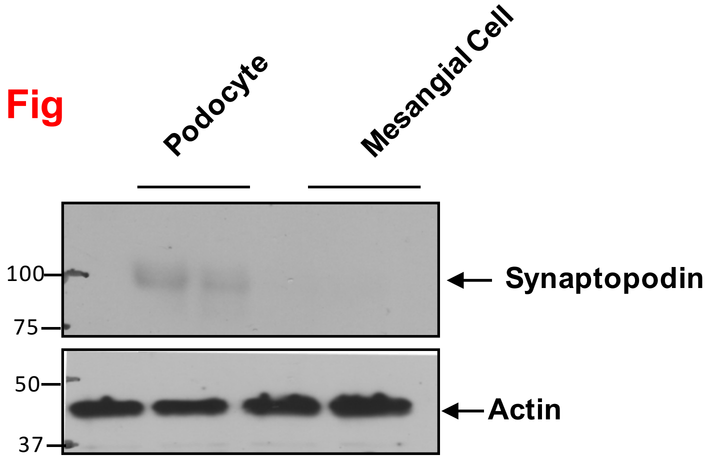

S1B Fig.

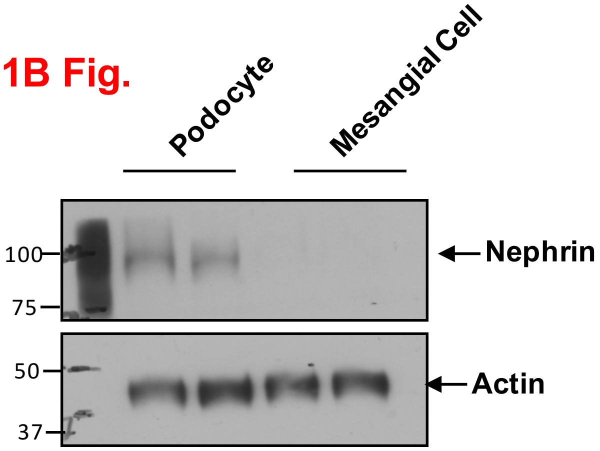

S6A Fig.

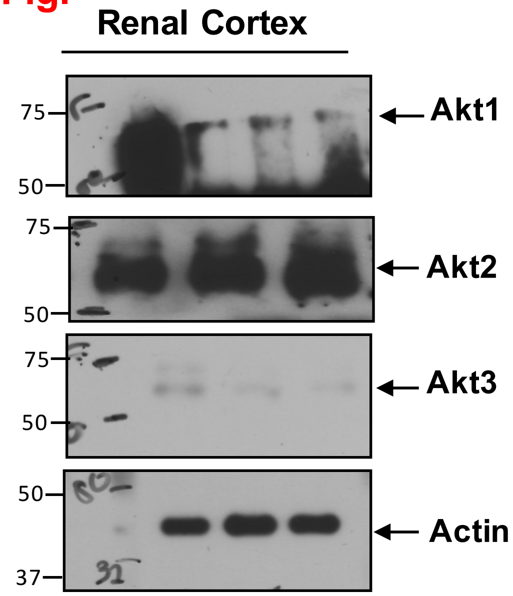

S6B Fig.

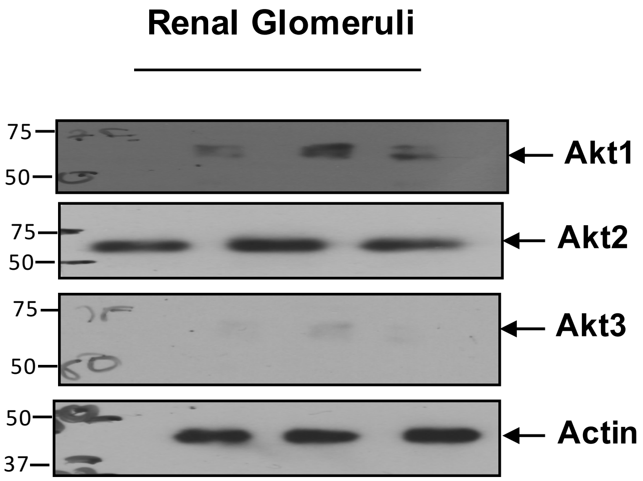

S8 Fig.

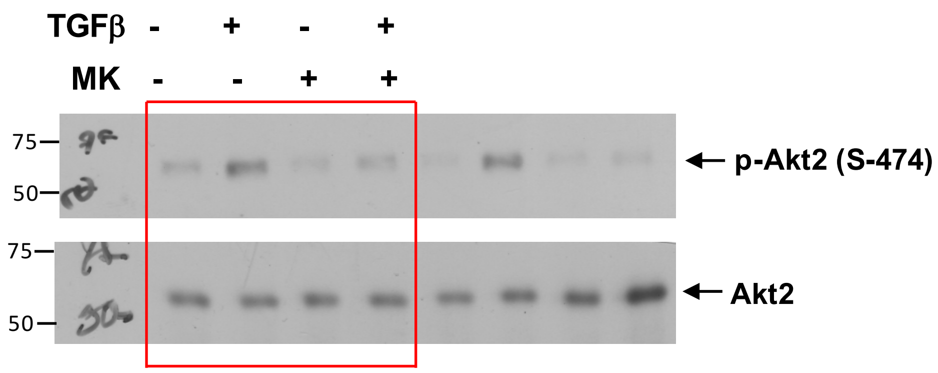

S13A Fig.

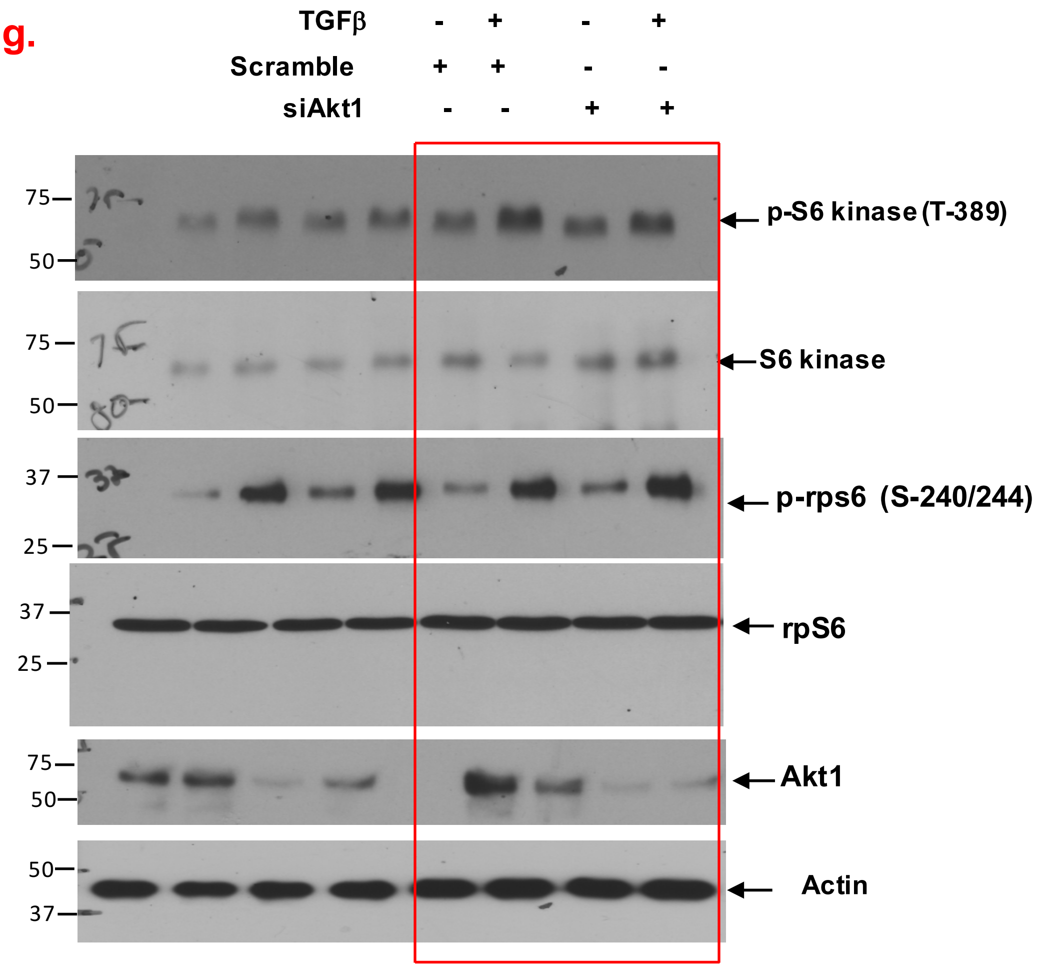

S13B Fig.

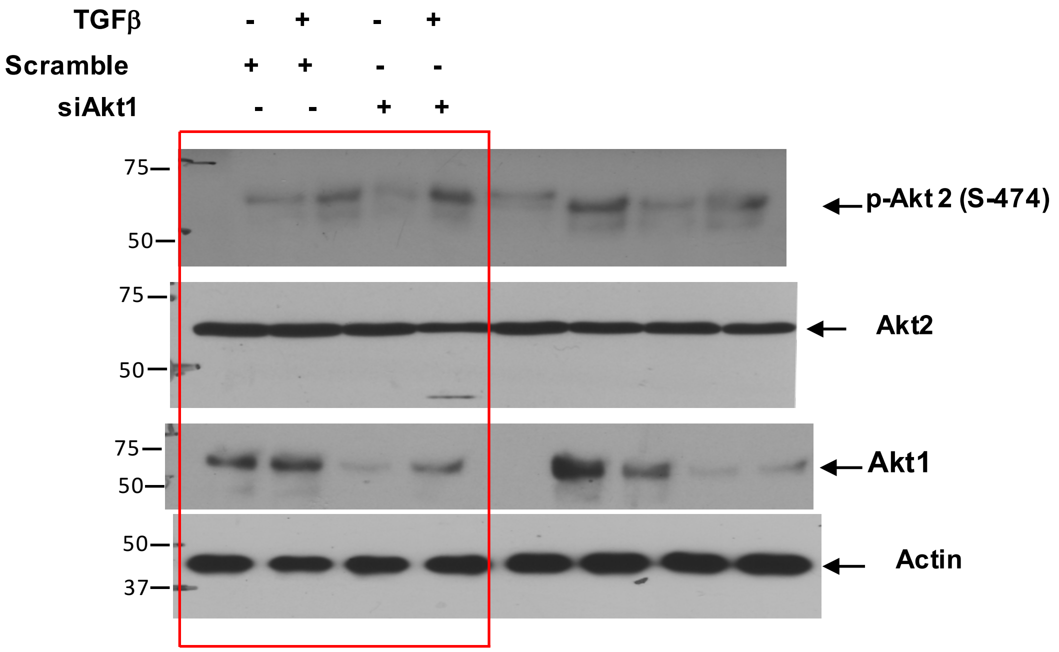

Supplement: S1 Data — (PDF) [file pone.0207285.s014.pdf]
